# Supplementary material for: Exploring Methods to Evaluate HPAI Transmission Risk in Iowa During Peak HPAI Incidence, February 2022–December 2023
Source: Int J Environ Res Public Health. 2025 Mar 10;22(3):400. doi: 10.3390/ijerph22030400 (PMC11942192; doi:10.3390/ijerph22030400)
Supplement: Supplementary file 1 [file ijerph-22-00400-s001.zip › File S1.pdf]

USDA

United States Department of Agriculture

Marketing and Regulatory Programs

Animal and Plant Health Inspection Service

Legislative and Public Affairs

Freedom of Information

4700 River Road
Unit 50
Riverdale, MD
20737-1232

March 8, 2024

Christopher Jimenez
SUNY Downstate Health Sciences University
450 Clarkson Avenue
Brooklyn, NY 11203
Email: christopher.jimenez@downstate.edu

Dear Mr. Jimenez:

This is the final response to your Freedom of Information Act (FOIA) request dated and received in this office on January 4, 2024, and assigned tracking number **2024-APHIS-01723-F.** You requested a copy of the following documents/data below:

> *•Location data (address or coordinates) for all World Organization for Animal Health (WOAH) poultry, and non-poultry, sites that work with APHIS.*
> *• Data describing which sites experienced an HPAI outbreak.*

Time period from Jan 1, 2022-December 31,2023.

Your request was forwarded to the Veterinary Services (VS) program office to search for responsive records. VS employees conducted a thorough search of their electronic files on January 11, 2024, and located the enclosed **108 pages** in responsive to your request. These records are being fully released to you.

Also, you can check on this website here: USDA APHIS | 2022-2024 Confirmations of Highly Pathogenic Avian Influenza in Commercial and Backyard Flocks

This completes APHIS's response to your request. If you have any questions or concerns, you may contact Sophana Lau-Lopez, the analyst who processed your request, by email, at sophana.n.lau_lopez@usda.gov, as well as Ms. Abbey Fretz, our FOIA Public Liaison, at (301) 851-4100. Additionally, you may contact the Office of Government Information Service (OGIS) at the National Archives and Records Administration to inquire about the FOIA mediation services they offer. The contact information for OGIS is as follows: Office of Government Information Services, National Archives and Records Administration, 8601 Adelphi Road-OGIS, College Park, Maryland 20740-6001, e-mail at ogis@nara.gov; telephone at 202-741-5770; toll free at 1-877-684-6448; or facsimile at 202-741-5769.

If you are not satisfied with this response, you may submit an administrative appeal. APHIS would prefer that you submit an administrative appeal by email to: FOIA.MRP.Appeals@usda.gov. In the alternative, if you can't email your appeal, you may submit your appeal in writing to: **Administrator, Animal and Plant Health Inspection Service, Ag Box 3401, Washington, DC 20250-3401.**

Your appeal must be postmarked or electronically transmitted within 90 days of the date of this response letter. If you choose to appeal, please refer to FOIA **#2024-APHIS-01723-F** in your appeal letter and add the words "**FOIA Appeal**" to the front of the envelope. To assist the Administrator in reviewing your appeal, please provide specific reasons why you believe modification of the determination is warranted.

Sincerely,

AVRA LEONARD Digitally signed by AVRA LEONARD Date: 2024.03.08 10:43:00 -05'00'

For:
Tonya G. Woods
Director
Freedom of Information & Privacy Act
Legislative and Public Affairs

Enclosures
