# Supplementary material for: Exploring Methods to Evaluate HPAI Transmission Risk in Iowa During Peak HPAI Incidence, February 2022–December 2023
Source: Int J Environ Res Public Health. 2025 Mar 10;22(3):400. doi: 10.3390/ijerph22030400 (PMC11942192; doi:10.3390/ijerph22030400)
Supplement: Supplementary file 1 [file ijerph-22-00400-s001.zip › File S2.pdf]

OA - MRP - HPAI 2022-23 Report

Data as of January 04, 2024 at 12:00 PM ET

To date, APHIS confirmed HPAI in **47** states, in **608** commercial[1], and **455** backyard/pet bird[2] flocks.
There are a total of **1063** infected premises and **838** Control Areas/Surveillance Zones[3] released.

NAHLN Detection	Confirmed by NVSL	State	Location	Type	Species	Count	Status	Approximate Indemnity [4]	Control Area/WOAH Surveillance Zone Release Date [5]	Notes [6]
1/3/2024		Wisconsin	Washburn 01	Commercial Turkey Meat Bird	Turkey	0				
12/29/2023		Kansas	Rice 04	Commercial Table Egg Pullets	Chicken	125,000				
12/30/2023	1/3/2024	California	Sonoma 12	Commercial Broiler Production	Chicken	77,862	Depopulation completed 1/02 (Foam)	$581,196		
12/28/2023	1/3/2024	California	Merced 09	Commercial Broiler Production	Chicken	534,777	Depopulation completed 12/31 (CO2 Cart/Container)	$2,655,535		
12/28/2023	12/30/2023	South Dakota	Edmunds 12	Commercial Upland Gamebird Producer	Pheasant	1,400	Depopulation completed (1/02 Cervical Dislocation)	$24,377		
12/28/2023	12/29/2023	North Dakota	McIntosh 03	Backyard	WOAH NP	110	Depopulation completed 12/29 (CO2 Cart/Container)			
12/29/2023	1/3/2024	California	Merced 08	Commercial Table Egg Pullets	Chicken	764,288		$9,427,683		Depopulation via CO2 Cart/Container starter 1/02
12/28/2023	12/29/2023	Texas	Carson 01	Backyard	WOAH NP	27	Depopulation completed 12/29 (CO2 Cart/Container)			
12/27/2023	12/28/2023	Montana	Missoula 03	Backyard	WOAH NP	6	No depopulation planned.			
12/26/2023	12/28/2023	Missouri	Audrain 01	Backyard	Poultry	334	Depopulation completed 12/28) Humane/Controlled Slaughter)	$0		

[1] Commercial = WOAH Poultry; This includes NPIP commercial, independent commercial, or backyard poultry.
[2] Backyard/Pet Bird = WOAH Non-Poultry (not commercial). There are a few premises designated as pet bird Non-Poultry.
[3] Surveillance zones were in place as a requirement for Non-Poultry premises prior to November 15.
[4] Indemnity estimates for animals do not include depopulation, disposal, virus elimination costs, or materials destroyed.
[5] Commercial and Backyard Poultry premises have control zones in place. Backyard Non-Poultry premises have surveillance zones.
[6] Dangerous contact designates a premises with likely exposure or strong epidemiological links to an infected flock. The flock is depopulated based on the potential exposure or link rather than waiting for testing.

12/26/2023	12/28/2023	California	Sonoma 11	Commercial Table Egg Layer	Chicken	37,266	Depopulation completed Depopulation completed 1/02 (CO2 Cart/Container)	$682,340		
12/26/2023	No Further Testing	Minnesota	Todd 11	Commercial Turkey Meat Bird	Turkey	48,500	Depopulation completed 12/27 (Foam)	$678,559		
12/24/2023	12/28/2023	California	Marin 01	Commercial Table Egg Pullets	Chicken	151,043	Depopulation completed 1/02 (Primary depopulation method used was foaming followed with secondary cervical dislocation if necessary)	$928,065		
12/26/2023	12/27/2023	Michigan	Muskegon 03	Commercial Turkey Meat Bird	Turkey	30,969	Depopulation completed 12/27 (VSD+ + See note)	$1,022,611		Approximately 170 birds were not deceased following VSD+, so a secondary AVMA approved method was used.
12/26/2023	12/27/2023	Oklahoma	Garfield 01	Backyard	WOAH NP	26	No depopulation occurred. All birds died.			
12/24/2023	12/27/2023	Ohio	Hardin 02	Commercial Table Egg Pullets	Chicken	1,363,864	Depopulation completed 12/29 (VSD+ and CO2)	$5,699,917		
12/24/2023	12/27/2023	Minnesota	Todd 10	Commercial Turkey Breeder Hens	Turkey	78,803	Depopulation completed 12/26 (Foam)	$1,313,340		
12/23/2023	12/28/2023	California	Sonoma 10	Commercial Table Egg Layer	Chicken	52,358	Depopulation completed 12/29 (CO2 Cart/Container)	$988,740		
12/22/2023	12/27/2023	Pennsylvania	Northumberland 02	Commercial Raised for Release Upland Game Bird	Pheasant	98,275	Depopulation completed 12/27 (Foam and long netting)	$1,687,211		Depopulation via foam/gunshot/longnetting started on 12/23.
12/21/2023	12/26/2023	California	Merced 07	Commercial Table Egg Layer	Chicken	708,985	Depopulation completed 12/29 (VSD+, CO2 carts, and cervical dislocation)	$9,713,946		Depopulation via VSD+ heat started on 12/26.
12/20/2023	12/21/2023	Nebraska	Dodge 01	Backyard	WOAH NP	41	Depopulation completed 12/21 (Gunshot)			
12/19/2023	12/21/2023	South Dakota	Edmunds 11	Commercial Upland Gamebird Producer	Pheasant	3,807		$66,051		Depopulation via cervical dislocation pending start.
12/19/2023	12/20/2023	Iowa	Mahaska 01	Backyard	WOAH NP	27	Depopulation completed 12/18 (Cervical dislocation)	$63		
12/18/2023	12/20/2023	South Dakota	Charles Mix 09	Commercial Upland Gamebird Producer	Pheasant	50	Depopulation completed 12/19 (Cervical dislocation)			

12/19/2023	12/20/2023	California	Merced 06	Commercial Turkey Meat Bird	Turkey	73,714	Depopulation completed 12/23 (CO2)	$1,988,361		
12/19/2023	12/20/2023	California	Merced 05	Commercial Table Egg Layer	Chicken	243,253		$1,908,907		Depopulation via VSD+ heat, CO2, and cervical dislocation started on 12/21.
12/18/2023	12/20/2023	Oregon	Benton 01	Backyard	WOAH NP	53	Depopulation completed 12/19 (CO2)	$350		
12/17/2023	12/19/2023	Kansas	McPherson 03	Backyard	Poultry	20,000	Depopulation completed 12/19 (CO2)	$377,149		
12/17/2023	12/19/2023	Kansas	Barton 02	Backyard	WOAH NP	73	No depopulation planned.			120-day Quarantine; no depopulation
12/18/2023	12/19/2023	Michigan	Muskegon 02	Commercial Turkey Meat Bird	Turkey	47,878	Depopulation completed 12/20 (VSD+ heat)	$1,191,043		There was a secondary method; need to figure out what it was.
12/16/2023	12/18/2023	Kansas	Rice 02	Backyard	WOAH NP	100	Depopulation completed 12/17 (CO2)	$179		
12/16/2023	12/19/2023	California	Lassen 01	Backyard	WOAH NP	25	No depopulation occurred. All birds died.			
12/16/2023	12/19/2023	California	Sonoma 09	Commercial Duck Meat Bird	Duck	3,565	Depopulation completed 12/18 (CO2)	$3,275		
12/15/2023	12/18/2023	California	Sonoma 08	Commercial Table Egg Layer	Chicken	494,238		$4,816,037		Depopulation via VSD+ heat and cervical dislocaiton started on 12/20.
12/15/2023	12/19/2023	Washington	King 09	Backyard	Poultry	85	Depopulation completed 12/18 (CO2)	$2,032		
12/14/2023	12/18/2023	South Dakota	Edmunds 10	Commercial Upland Gamebird Producer	Pheasant	1,018	Depopulation completed 12/16 (Cervical dislocation)			
12/15/2023	12/18/2023	California	Sonoma 07	Backyard	Poultry	38,129	Depopulation completed 12/16 (CO2)	$508,140		
12/13/2023	12/15/2023	Idaho	Latah 01	Backyard	Poultry	200	Depopulation completed 12/14 (CO2)	$3,640		
12/17/2023	12/18/2023	Kansas	Rice 03	Commercial Table Egg Layer	Chicken	800,000		$8,075,184		Depopulation via VSD+ heat started on 12/20.
12/13/2023	12/15/2023	Minnesota	Waseca 04	Backyard	WOAH NP	91	No depopulation planned.			120-day Quarantine; no depopulation
12/13/2023	12/14/2023	Arkansas	Scott 01	Backyard	WOAH NP	176	Depopulation completed 12/15 (CO2/cervical dislocation/gunshot)			
12/12/2023	12/14/2023	Oregon	Clatsop 01	Backyard	WOAH NP	30	Depopulation completed 12/13 (CO2)			
12/12/2023	12/13/2023	Colorado	Weld 10	Backyard	WOAH NP	26	Depopulation completed 12/13 (CO2)	$386		
12/12/2023	12/14/2023	Ohio	Darke 03	Commercial Table Egg Layer	Chicken	560,000	Depopulation completed 12/15 (VSD+ heat/cervical dislocation)	$7,481,186		

12/12/2023	12/12/2023	New York	Dutchess 02	Backyard	Poultry	4,178	Depopulation completed 12/15 (CO2/mechanically assisted cervical dislocation)	$76,642	1/3/2024	
12/11/2023	12/13/2023	California	Merced 04	Commercial Broiler Production	Chicken	183,110	Depopulation completed 12/19 (CO2)	$1,424,596		
12/11/2023	12/13/2023	California	Merced 03	Commercial Table Egg Layer	Chicken	1,358,570	Depopulation completed 12/22 (VSD+ heat)	$14,021,354		
12/11/2023	12/13/2023	Kansas	McPherson 02	Backyard	Poultry	20,000	Depopulation completed 12/14 (CO2)	$292,960		
12/11/2023	12/13/2023	Kansas	Rice 01	Commercial Table Egg Layer	Chicken	700,000	Depopulation completed 12/24 (VSD+ heat/CO2)	$7,026,543		
12/9/2023	12/12/2023	California	San Joaquin 04	Commercial Duck Breeder	Duck	5,952	Depopulation completed 12/14 (Cervical dislocation)	$34,054		
12/8/2023	12/12/2023	Missouri	Saint Louis 01	Backyard	WOAH NP	16	Depopulation completed 12/9 (CO2)			
12/8/2023	12/12/2023	Alabama	Etowah 01	Backyard	WOAH NP	82	Depopulation completed 12/9 (Mechanically assisted cervical dislocation)	$3,170		
12/8/2023	12/12/2023	South Dakota	Lake 03	Commercial Turkey Meat Bird	Turkey	76,022	Depopulation completed 12/10 (VSD+ heat/foam)	$1,502,222		This is a previously affected premises (Lake 01).
12/8/2023	12/8/2023	Kansas	Barton 01	Backyard	WOAH NP	31	No depopulation planned.			120-day Quarantine; no depopulation
12/7/2023	12/11/2023	Minnesota	Olmsted 01	Commercial Turkey Breeder Hens	Turkey	15,098	Depopulation completed 12/8 (Foam)	$706,189		
Sample sent directly to NVSL	12/7/2023	Vermont	Orleans 01	Backyard	WOAH NP	40	Depopulation completed 12/8 (CO2)	$651		
12/5/2023	12/7/2023	California	Stanislaus 05	Commercial Broiler Production	Chicken	258,552	Depopulation completed 12/8 (CO2)	$1,202,267	12/25/2023	
12/6/2023	12/8/2023	Wisconsin	Barron 09	Commercial Turkey Meat Bird	Turkey	70,020	Depopulation completed 12/7 (Foam)	$2,315,017		
12/6/2023	12/8/2023	South Dakota	Charles Mix 08	Commercial Turkey Meat Bird	Turkey	32,411	Depopulation completed 12/7 (Foam)	$848,124		
12/5/2023	12/7/2023	California	Sonoma 06	Commercial Duck Meat Bird	Duck	4,870	Depopulation completed 12/8 (CO2)	$5,460		
12/6/2023	12/7/2023	Michigan	Montmorency 01	Backyard	Poultry	103	Depopulation completed 12/8 (CO2)			

12/6/2023	12/8/2023	Iowa	Mills 01	Backyard	WOAH NP	11	Depopulation completed 12/6 (Cervical Dislocation)			
12/5/2023	12/7/2023	Arkansas	Carroll 02	Commercial Turkey Meat Bird	Turkey	33,296	Depopulation completed 12/6 (Foam)	$550,116	12/28/2023	
12/5/2023	12/7/2023	Ohio	Darke 02	Commercial Turkey Meat Bird	Turkey	15,187	Depopulation completed 12/6 (Foam)	$552,655	12/28/2023	
12/5/2023	12/7/2023	Ohio	Hardin 01	Commercial Table Egg Layer	Chicken	2,613,580	Depopulation completed 12/15 (VSD+ heat/CO2/cervical dislocation)	$14,057,754		
12/4/2023	12/5/2023	Kansas	Shawnee 02	Backyard	WOAH NP	66	Depopulation completed 12/7 (CO2)	$514		
12/5/2023	12/6/2023	Minnesota	Todd 09	Commercial Turkey Breeder Toms	Turkey	64,048	Depopulation completed 12/6 (Foam/mechanically assisted cervical dislocation)	$742,887	12/23/2023	
12/5/2023	12/6/2023	California	Sonoma 05	Commercial Table Egg Layer	Chicken	232,000	Depopulation completed 12/6 (CO2)	$1,997,531		
12/4/2023	12/6/2023	South Dakota	Hamlin 05	Commercial Turkey Meat Bird	Turkey	56,221	Depopulation completed 12/5 (VSD+ heat/foam)	$1,596,098	12/24/2023	
12/2/2023	12/6/2023	South Dakota	Bon Homme 03	Commercial Turkey Meat Bird	Turkey	47,577	Depopulation completed 12/4 (VSD+ heat/foam)	$1,204,781	12/26/2023	
12/3/2023	12/5/2023	Minnesota	Otter Tail 11	Commercial Turkey Meat Bird	Turkey	26,275	Depopulation completed 12/4 (Foam/mechanically assisted cervical dislocation)	$970,630	12/23/2023	
12/3/2023	12/5/2023	South Dakota	Spink 10	Commercial Turkey Meat Bird	Turkey	26,610	Depopulation completed 12/4 (Foam)	$554,688	12/24/2023	
12/3/2023	12/5/2023	California	Stanislaus 04	Commercial Broiler Production	Chicken	239,893	Depopulation completed 12/5 (CO2)	$1,115,502	12/26/2023	
12/2/2023	12/6/2023	Minnesota	Becker 07	Commercial Breeder Operation	Turkey	20,595	Depopulation completed 12/3 (Foam/mechnically assisted cervical dislocation)	$1,378,098	12/27/2023	
12/1/2023	12/5/2023	South Dakota	Kingsbury 06	Backyard	WOAH NP	370	Depopulation completed 12/2 (Cervical dislocation)	$6,522		
12/2/2023	12/5/2023	California	Stanislaus 03	Commercial Broiler Production	Chicken	254,360	Depopulation completed 12/2 (CO2)	$1,182,774	12/25/2023	
12/1/2023	12/1/2023	Idaho	Canyon 12	Backyard	WOAH NP	17	No depopulation planned.			120-day Quarantine; no depopulation
12/1/2023	12/4/2023	Kansas	Pottawatomie 01	Backyard	WOAH NP	34	No depopulation planned.	$0		120-day Quarantine; no depopulation

11/30/2023	12/4/2023	South Dakota	McPherson 10	Commercial Turkey Meat Bird	Turkey	40,755	Depopulation completed 12/1 (VSD+ heat/foam)	$782,089	12/20/2023	
11/30/2023	12/4/2023	Wisconsin	Barron 08	Commercial Turkey Meat Bird	Turkey	113,827	Depopulation completed 12/1 (Foam)	$2,895,954		
11/29/2023	12/1/2023	Michigan	Bay 01	Backyard	WOAH NP	60	Depopulation completed 11/30 (CO2)	$1,072		
11/29/2023	12/1/2023	Ohio	Darke 01	Commercial Turkey Meat Bird	Turkey	33,000	Depopulation completed 12/1 (Foam/mechanically assisted cervical dislocation)	$12,000,870	12/30/2023	
11/29/2023	11/30/2023	South Dakota	Minnehaha 01	Backyard	WOAH NP	80	Depopulation completed 11/30 (Cervical Dislocation)	$1,847		
11/29/2023	12/4/2023	Arkansas	Johnson 01	Commercial Broiler Production	Chicken	108,984	Depopulation completed 12/2 (Foam/cervical dislocation)	$719,280	12/28/2023	
11/29/2023	11/30/2023	Colorado	Bent 01	Backyard	Poultry	4,050	Depopulation completed 12/1 (Foam/CO2)	$24,520	12/15/2023	
11/28/2023	11/30/2023	California	San Benito 01	Commercial Duck Breeder	Duck	5,025	Depopulation completed 12/1 (CO2)		12/22/2023	
11/28/2023	11/30/2023	California	Sonoma 04	Commercial Table Egg Layer	Chicken	82,471	Depopulation completed 12/5 (CO2)	$755,434		
11/29/2023	12/1/2023	Iowa	Woodbury 02	Backyard	WOAH NP	39	Depopulation completed 11/30 (Cervical Dislocation)			
11/28/2023	11/29/2023	Iowa	Woodbury 01	Backyard	WOAH NP	10	No depopulation occurred. All birds died.			
11/28/2023	11/30/2023	Montana	Lewis and Clark 01	Backyard	WOAH NP	6	Depopulation completed 11/30 (CO2)	$121		
11/28/2023	11/29/2023	North Dakota	Dickey 05	Backyard	WOAH NP	128	Depopulation completed 11/29 (Cervical Dislocation)		1/3/2024	
11/28/2023	11/29/2023	Colorado	Morgan 01	Backyard	WOAH NP	59	No depopulation planned.	$0		120-day Quarantine; no depopulation
11/27/2023	11/29/2023	Arkansas	Carroll 01	Commercial Broiler Production	Chicken	81,222	Depopulation completed 11/28 (Foam)	$584,996	12/23/2023	
11/27/2023	11/29/2023	Minnesota	Becker 06	Commercial Turkey Breeder Hens	Turkey	23,165	Depopulation completed 11/28 (Foam/mechanically assisted cervical dislocation)	$3,055,957	12/27/2023	
11/27/2023	11/28/2023	North Dakota	Emmons 01	Backyard	WOAH NP	60	No depopulation planned.	$0		120-day Quarantine; no depopulation
11/27/2023	11/28/2023	South Dakota	Yankton 02	Commercial Turkey Meat Bird	Turkey	48,962	Depopulation completed 11/28 (Foam)	$1,167,730	12/22/2023	

11/25/2023	11/28/2023	Wisconsin	Trempealeau 03	Commercial Turkey Meat Bird	Turkey	48,692	Depopulation completed 11/27 (VSD+ heat)	$1,559,671		
11/24/2023	11/28/2023	North Dakota	Dickey 04	Commercial Turkey Meat Bird	Turkey	60,294	Depopulation completed 11/25 (Foam & VSD+)	$1,469,793	1/3/2024	
11/25/2023	11/27/2023	California	Sonoma 03	Commercial Duck Breeder	Duck	205,488	Depopulation completed 11/29 (Foam)	$215,660	12/29/2023	
11/24/2023	11/28/2023	South Dakota	McPherson 09	Commercial Turkey Meat Bird	Turkey	61,769	Depopulation completed 11/25 (Foam)	$1,008,615	12/20/2023	
11/24/2023	11/29/2023	Minnesota	Otter Tail 10	Commercial Turkey Meat Bird	Turkey	18,806	Depopulation completed 11/25 (Foam & KEDS)	$687,771	12/18/2023	
11/24/2023	11/29/2023	Minnesota	Douglas 01	Commercial Turkey Meat Bird	Turkey	13,526	Depopulation completed 11/25 (Foam & KEDS)	$428,093	12/20/2023	
11/23/2023	11/27/2023	Minnesota	Brown 05	Commercial Turkey Breeder Hens	Turkey	7,793	Depopulation completed 11/24 (Foam)	$582,972	12/14/2023	
11/22/2023	11/28/2023	Nebraska	Colfax 01	Backyard	WOAH NP	19	Depopulation completed 11/23 (Firearm)	$0		
11/16/2023	11/17/2023	South Dakota	Walworth 01	Backyard	WOAH NP		Depopulated by producer	$0		
11/22/2023	11/27/2023	Iowa	Sioux 01	Commercial Table Egg Layer	Poultry	1,616,259	Depopulation completed 11/25 (VSD+ & Cervial Disloaction)	$24,167,323	1/2/2024	
11/22/2023	11/28/2023	Washington	Pierce 07	Backyard	WOAH NP	538	Depopulation completed 11/27 (CO2 Container)	$16,777		
11/22/2023	11/28/2023	Wisconsin	Trempealeau 02	Commercial Turkey Meat Bird	Turkey	23,497	Depopulation completed 11/22 (Foam)	$885,056		
11/22/2023	11/24/2023	Minnesota	Freeborn 02	Backyard	WOAH NP	8	Depopulation completed 11/24 (KEDS)	$132		
11/21/2023	11/24/2023	Oregon	Clackamas 02	Backyard	WOAH NP	11	Depopulation completed 11/22 (Firearm)	$153		
11/21/2023	11/24/2023	Minnesota	Otter Tail 09	Commercial Turkey Meat Bird	Turkey	19,417	Depopulation completed 11/22 (Foam & TEDS)	$1,573,677	12/13/2023	
11/21/2023	11/24/2023	Minnesota	Kandiyohi 13	Commercial Turkey Breeder Hens	Turkey	7,271	Depopulation completed 11/22 (Foam)	$441,875	12/22/2023	
11/16/2023	11/20/2023	South Dakota	Day 02	Backyard	Poultry	60	Depopulation completed 11/17 (Foam)			
11/20/2023	11/21/2023	Maryland	Caroline 01	Commercial Broiler Production	Chicken	192,927	Depopulation completed 11/21 (Foam & VSD+)	$1,541,607	12/17/2023	
11/20/2023	11/22/2023	Wisconsin	Trempealeau 01	Commercial Turkey Meat Bird	Turkey	51,256	Depopulation completed 11/21 (VSD+)	$1,389,425		

11/20/2023	11/22/2023	South Dakota	Brule 04	Commercial Turkey Meat Bird	Turkey	45,063	Depopulation completed 11/22 (Foam & VSD+)	$1,139,682	12/15/2023	
11/20/2023	11/22/2023	Minnesota	Stearns 16	Commercial Turkey Meat Bird	Turkey	34,207	Depopulation completed 11/21 (Foam)	$1,244,538	12/7/2023	
11/20/2023	11/21/2023	Georgia	Sumter 01	Commercial Raised for Release Waterfowl	Duck	30,000	Depopulation completed 11/25 (Foam & CO2 Container)	$27,300	12/14/2023	Designation changed from backyard poultry to commercial raised for release waterfowl. No change in count.
11/20/2023	11/21/2023	South Dakota	Clark 11	Commercial Turkey Meat Bird	Turkey	57,413	Depopulation completed 11/21 (Foam & VSD+)	$1,127,318	12/9/2023	
11/20/2023	11/22/2023	Minnesota	Otter Tail 08	Commercial Turkey Meat Bird	Turkey	27,998	Depopulation completed 11/20 (VSD+)	$953,236	12/13/2023	
11/19/2023	11/21/2023	South Dakota	Kingsbury 05	Commercial Turkey Meat Bird	Turkey	28,794	Depopulation completed 11/21 (Foam & VSD+)	$587,073	12/12/2023	
11/19/2023	11/21/2023	Minnesota	Brown 04	Commercial Turkey Breeder Hens	Turkey	6,379	Depopulation completed 11/20 (Foam)	$637,696	12/13/2023	
11/18/2023	11/21/2023	Ohio	Union 01	Commercial Table Egg Layer	Chicken	1,348,857	Depopulation completed 11/23 (CO2 Container & VSD+)	$11,743,614		
11/18/2023	11/21/2023	South Dakota	Clark 10	Commercial Turkey Meat Bird	Turkey	23,621	Depopulation completed 11/20 (Foam & VSD+)	$641,538	12/7/2023	
11/18/2023	11/21/2023	South Dakota	Beadle 12	Commercial Turkey Meat Bird	Turkey	78,168	Depopulation completed 11/21 (Foam & VSD+)	$1,706,072	12/13/2023	
11/17/2023	11/21/2023	Texas	Grimes 01	Backyard	WOAH NP	56	Depopulation completed 11/20 (CO2 Container)			
11/17/2023	11/21/2023	Florida	Broward 06	Backyard	WOAH NP	5	Depopulation completed 11/20 (CO2 Container)	$0		
11/17/2023	11/20/2023	South Dakota	Edmunds 09	Commercial Turkey Meat Bird	Turkey	33,392	Depopulation completed 11/20 (VSD+ & Foam)	$848,465	12/7/2023	
11/17/2023	11/21/2023	Minnesota	Big Stone 02	Commercial Turkey Meat Bird	Turkey	78,486	Depopulation completed 11/18 (Foam & KEDS)	$2,225,340	12/15/2023	
11/17/2023	11/20/2023	New Mexico	San Juan 01	Backyard	WOAH NP	17	No depopulation planned.	$0		
11/16/2023	11/20/2023	Wisconsin	Taylor 01	Backyard	WOAH NP	8	Depopulated by producer 11/17	$0		
11/17/2023	11/20/2023	California	Fresno 08	Commercial Duck Breeder	Duck	23,378	Depopulation completed 11/21 (Foam)	$133,722	12/29/2023	
11/17/2023	11/21/2023	Minnesota	Kandiyohi 12	Commercial Turkey Meat Bird	Turkey	8,447	Depopulation completed 11/17 (Foam & KEDS)	$561,653	12/23/2023	

11/16/2023	11/20/2023	South Dakota	Edmunds 08	Commercial Turkey Breeder Hens	Turkey	19,209	Depopulation completed 11/17 (VSD+)	$444,304	12/14/2023	
11/15/2023	11/20/2023	South Dakota	Edmunds 07	Commercial Turkey Breeder Replacement Hens	Turkey	30,104	Depopulation completed 11/16 (VSD+)	$2,485,817	12/8/2023	
11/15/2023	11/16/2023	Florida	Duval 03	Backyard	WOAH NP	52	Depopulation completed 11/17 (CO2 Container)	$3,185		
11/15/2023	11/17/2023	South Dakota	Fall River 01	Backyard	WOAH NP	78	Depopulation completed 11/16 (Cervical Dislocation)	$1,649		
11/15/2023	11/20/2023	Minnesota	Swift 07	Commercial Turkey Meat Bird	Turkey	134,349	Depopulation completed 11/16 (Foam and KEDS)	$488,960	12/12/2023	
11/15/2023	11/16/2023	Oregon	Marion 02	Commercial Broiler Production	Chicken	123,494	Depopulation completed 11/17 (Cervical Dislocation & VSD+)	$574,247	12/8/2023	
11/14/2023	11/15/2023	Michigan	Cass 01	Backyard	WOAH NP	15	Depopulation completed 11/15 (Injectable)	$1,850		
11/14/2023	11/16/2023	Iowa	Benton 01	Backyard	WOAH NP	86	Depopulation completed 11/16 (CO2 Container and Firearm)	$0		
11/14/2023	11/15/2023	North Dakota	McIntosh 02	Backyard	Poultry	96	No depopulation occurred. All birds died.	$0	12/21/2023	120-day Quarantine; no depopulation
11/14/2023	11/16/2023	Minnesota	Stearns 15	Commercial Turkey Meat Bird	Turkey	43,132	Depopulation completed 11/15 (Foam)	$1,084,885	12/2/2023	
11/13/2023	11/16/2023	Oregon	Linn 04	Commercial Broiler Production	Chicken	630,737	Depopulation completed 11/18 (Foam & VSD+)	$2,885,195	12/29/2023	
11/13/2023	11/15/2023	South Dakota	Edmunds 06	Commercial Turkey Breeder Replacement Hens	Turkey	24,521	Depopulation completed 11/15 (VSD+)	$2,443,272	12/8/2023	
11/13/2023	11/15/2023	North Dakota	LaMoure 04	Commercial Turkey Meat Bird	Turkey	25,107	Depopulation completed 11/15 (VSD+ & Foam)	$562,432	12/22/2023	
Sample sent directly to NVSL	11/13/2023	Idaho	Bonneville 02	Backyard	WOAH NP	50	No depopulation planned.	$0		120-day Quarantine; no depopulation
11/9/2023	11/14/2023	Oregon	Marion 01	Backyard	WOAH NP	5	No depopulation occurred. All birds died.	$0		

11/12/2023	11/14/2023	Minnesota	Redwood 05	Commercial Turkey Meat Bird	Turkey	18,731	Depopulation completed 11/13 (Foam)	$557,622	12/2/2023	
11/12/2023	11/14/2023	Minnesota	Kandiyohi 11	Commercial Turkey Meat Bird	Turkey	35,638	Depopulation completed 11/14 (Foam)	$1,431,248	12/7/2023	
11/10/2023	11/14/2023	Texas	Ellis 01	Backyard	WOAH NP	46	No depopulation planned.	$0		120-day Quarantine; no depopulation
11/10/2023	11/14/2023	Missouri	Jasper 03	Commercial Turkey Meat Bird	Turkey	29,318	Depopulation completed 11/11 (Foam)	$993,447	12/6/2023	
11/10/2023	11/13/2023	Colorado	Montrose 02	Backyard	WOAH NP	54	Depopulation completed 11/11 (CO2 Cart/ Container)			
11/10/2023	11/14/2023	Iowa	Cerro Gordo 01	Backyard	Poultry	75	Depopulation completed 11/11 (Cervical Dislocation)	$927		
11/10/2023	11/14/2023	Iowa	Kossuth 03	Backyard	Poultry	13,000	Depopulation completed 11/11 (VSD+)	$404,610	12/1/2023	
11/11/2023	11/14/2023	Iowa	Jones 01	Backyard	WOAH NP	23	Depopulation completed 11/10 (Cervical Dislocation)	$0		
11/9/2023	11/14/2023	Iowa	Taylor 02	Commercial Table Egg Layer	Chicken	1,121,096	Depopulation completed 11/12 (VSD+)	$2,836,072	1/2/2024	
11/9/2023	11/14/2023	Montana	Missoula 02	Backyard	WOAH NP	4	No depopulation planned.	$0		
11/9/2023	11/13/2023	North Dakota	McIntosh 01	Backyard	WOAH NP	50	Depopulation completed 11/11 (CO2 Cart/ Container)	$643		
11/8/2023	11/14/2023	Oregon	Deschutes 08	Backyard	WOAH NP	33	Depopulation completed 11/6 (Firearm & CO2 Container)	$454		
11/6/2023	11/8/2023	South Dakota	Clark 09	Commercial Upland Gamebird Producer	Pheasant	8,469	Depopulation completed 11/13 (Foam)	$319,361	12/7/2023	
11/7/2023	11/8/2023	Utah	Utah 03	Backyard	WOAH NP	56	No depopulation planned.	$0		120-day Quarantine; no depopulation
Sample sent directly to NVSL	11/8/2023	Alaska	Matanuska-Susitna 08	Backyard	WOAH NP	42	No depopulation planned.	$0		120-day Quarantine; no depopulation
11/7/2023	11/8/2023	Minnesota	Steele 01	Commercial Turkey Meat Bird	Turkey	46,925	Depopulation completed 11/9 (VSD+ & Foam)	$1,748,794	12/6/2023	
11/7/2023	11/9/2023	Montana	Wheatland 01	Backyard	Poultry	4,471	Depopulation completed 11/8 (Cervical Dislocation)	$73,408	12/21/2023	
11/7/2023	11/9/2023	Montana	Sweet Grass 01	Backyard	WOAH NP	3	No depopulation planned.	$0		120-day Quarantine; no depopulation
11/7/2023	11/9/2023	South Dakota	Roberts 02	Commercial Turkey Meat Bird	Turkey	51,223	Depopulation completed 11/8 (VSD+ & Foam)	$1,319,113	12/1/2023	

11/7/2023	11/9/2023	South Dakota	McPherson 08	Commercial Turkey Meat Bird	Turkey	58,358	Depopulation completed 11/9 (VSD+ & Foam)	$1,478,832	11/28/2023	
11/7/2023	11/9/2023	Minnesota	Redwood 04	Commercial Turkey Meat Bird	Turkey	18,451	Depopulation completed 11/8 (Foam & KEDS)	$669,576	11/30/2023	
11/6/2023	11/8/2023	South Dakota	Charles Mix 07	Commercial Turkey Meat Bird	Turkey	74,101	Depopulation completed 11/8 (VSD+ & Foam)	$1,878,460	11/29/2023	
11/6/2023	11/8/2023	Iowa	Kossuth 02	Commercial Upland Gamebird Producer	Pheasant	8,165	Depopulation completed 11/9 (Foam & Firearm)	$231,321	11/28/2023	
11/5/2023	11/7/2023	Minnesota	Stearns 14	Commercial Turkey Meat Bird	Turkey	70,874	Depopulation completed 11/6 (Foam & KEDS)	$1,642,230	11/24/2023	
11/2/2023	11/6/2023	Missouri	Benton 01	Commercial Broiler Breeder	Chicken	16,600	Depopulation completed 11/3 (Foam)	$129,148	12/14/2023	
11/2/2023	11/6/2023	Iowa	Clay 02	Backyard	Poultry	1,700	Depopulation completed 11/5 (Cervical Dislocation)	$8,580	12/2/2023	
11/2/2023	11/6/2023	Iowa	Hamilton 03	Backyard	Poultry	15,000	Depopulation completed 11/3 (VSD+)	$124,480	11/30/2023	This was updated to reflect NPIP designation from commercial broiler to backyard poultry due to small inventory count; does not change overall count.
11/2/2023	11/3/2023	South Dakota	McPherson 07	Commercial Turkey Breeder Hens	Turkey	26,804	Depopulation completed 11/5 (VSD+ & Foam)	$2,255,422	11/29/2023	
11/1/2023	11/3/2023	Iowa	Clay 03	Backyard	Poultry	8,270	Depopulation completed 11/7 (VSD+ & Cervical Dislocation)	$45,708	11/29/2023	
11/1/2023	11/3/2023	Iowa	Clay 01	Backyard	Poultry	7,660	Depopulation completed 11/7 (VSD+ & Cervical Dislocation)	$7,511	12/1/2023	
11/1/2023	11/2/2023	Alabama	Marshall 01	Commercial Broiler Breeder Pullets	Chicken	47,874	Depopulation completed 11/2 (Foam)	$222,614	12/5/2023	
11/1/2023	11/2/2023	Oklahoma	Grady 01	Backyard	WOAH NP	115	No depopulation planned.	$0		120-day Quarantine; no depopulation
10/31/2023	11/3/2023	Minnesota	Wright 01	Commercial Table Egg Layer	Chicken	940,017	Depopulation completed 11/7 (VSD+ & CO2 Cart)	$9,091,381	11/30/2023	
10/30/2023	10/31/2023	Arkansas	Madison 02	Commercial Broiler Breeder Pullets	Chicken	31,600	Depopulation completed 10/31 (Foam & Cervical Dislocation)	$245,848	11/24/2023	

10/30/2023	10/31/2023	Tennessee	Lincoln 01	Backyard	WOAH NP	100	No depopulation occurred. All birds died.	$0		120-day Quarantine; no depopulation
10/30/2023	11/1/2023	Iowa	Buena Vista 09	Commercial Turkey Meat Bird	Turkey	30,013	Depopulation completed 10/31 (Foam & KEDs)	$893,487	11/21/2023	
	No Testing - Dangerous Contact	Mississippi	Winston 01	Backyard	Poultry	464	Depopulation completed 10/31 (CO2/Container)	$3,725		
	No Testing - Dangerous Contact	Mississippi	Rankin 01	Backyard	Poultry	125	Depopulation completed 10/30 (CO2/Container)	$894		
	No Testing - Dangerous Contact	Mississippi	Lamar 01	Backyard	Poultry	1,128	Depopulation completed 10/28 (Cervical Dislocation)	$9,016		
	No Testing - Dangerous Contact	Mississippi	Clay 01	Backyard	Poultry	2,350	Depopulation completed 10/27 (Foam)	$23,072		
10/30/2023	10/31/2023	Oklahoma	Wagoner 01	Backyard	WOAH NP	50	No depopulation occurred. All birds died.	$0		
10/28/2023	10/31/2023	South Dakota	Beadle 11	Commercial Turkey Meat Bird	Turkey	29,668	Depopulation completed 10/30 (VSD+ & Foam)	$1,020,945	11/22/2023	
Sample sent directly to NVSL	10/28/2023	Alaska	Matanuska-Susitna 07	Backyard	WOAH NP	49	Depopulation completed 10/28 (CO2/Container)	$218		
Sample sent directly to NVSL	10/28/2023	Alaska	Matanuska-Susitna 06	Backyard	WOAH NP	30	Depopulation completed 10/28 (CO2/Container)	$0		
10/27/2023	10/31/2023	Minnesota	Meeker 12	Commercial Turkey Meat Bird	Turkey	32,073	Depopulation completed 10/28 (VSD+)	$1,167,136	11/18/2023	
10/27/2023	10/27/2023	Alabama	Chilton 01	Commercial Upland Gamebird Producer	Poultry	306,491	Depopulation completed 10/29 (Foam, CO2, KEDs, VSD+heat)	$3,655,857	12/1/2023	
	No Testing - Dangerous Contact	Louisiana	Tangipahoa 01	Backyard	Poultry	1,738	Depopulation completed 10/27 (Cervical Dislocation)	$17,351		
	No Testing - Dangerous Contact	Minnesota	Redwood 03	Commercial Turkey Meat Bird	Turkey	12,979	Depopulation completed 10/26 (Foam)	$150,248		
10/25/2023	10/25/2023	Oregon	Union 02	Backyard	WOAH NP	33	Depopulation completed 10/20 (CO2 Cart Container and Firearm)	$295		

10/25/2023	10/27/2023	Washington	KITTITAS 01	Backyard	Poultry	70	Depopulation completed 10/26 (CO2 Cart/Container)	$703	11/15/2023	
10/25/2023	10/26/2023	California	Merced 02	Commercial Turkey Meat Bird	Turkey	31,551	Depopulation completed 10/27 (CO2 Cart/Container)	$548,155	11/11/2023	
10/23/2023	10/24/2023	Iowa	Guthrie 02	Backyard	WOAH NP	47	Depopulation completed 10/23 (CO2 Cart/Container)	$0		
10/24/2023	10/25/2023	South Dakota	Hanson 06	Commercial Turkey Meat Bird	Turkey	13,431	Depopulation completed 10/26 (VSD+ & Foam)	$316,090	11/15/2023	
10/23/2023	10/25/2023	South Dakota	Beadle 10	Commercial Turkey Meat Bird	Turkey	51,078	Depopulation completed 10/24 (VSD+ & Foam)	$1,291,028	11/21/2023	
10/23/2023	10/25/2023	Minnesota	Brown 03	Backyard	WOAH NP	16	Depopulation completed 10/24 (KEDS)	$124		
10/23/2023	10/25/2023	South Dakota	Clark 08	Commercial Turkey Meat Bird	Turkey	31,100	Depopulation completed 10/25 (VSD+ & Foam)	$902,144	12/15/2023	
10/23/2023	10/25/2023	Minnesota	Meeker 11	Commercial Turkey Meat Bird	Turkey	34,443	Depopulation completed 10/25 (VSD+ & TEDS)	$1,253,380.77	11/18/2023	
10/22/2023	10/24/2023	Iowa	Pocahontas 01	Commercial Turkey Meat Bird	Turkey	50,012	Depopulation completed 10/24 (VSD+)	$1,819,936.68	11/16/2023	
10/20/2023	10/25/2023	Minnesota	Pine 01	Backyard	WOAH NP	32	Depopulation completed 10/22 (KEDS)	$636.14		
10/20/2023	10/23/2023	Iowa	Buena Vista 08	Commercial Turkey Meat Bird	Turkey	49,072	Depopulation completed 10/20 (VSD+)	$1,785,730.08	11/12/2023	
10/19/2023	10/24/2023	Minnesota	Kandiyohi 10	Commercial Turkey Meat Bird	Turkey	53,714	Depopulation completed 10/20 (Foam & KEDS)	$1,242,404.82	11/10/2023	
10/19/2023	10/24/2023	Minnesota	Blue Earth 04	Commercial Turkey Meat Bird	Turkey	11,862	Depopulation completed 10/20 (Foam & TEDS)	$431,003.16	11/17/2023	
10/18/2023	10/20/2023	Oregon	Union 01	Backyard	Poultry	100	No depopulation occurred. All birds died.	$0.00	11/13/2023	
10/18/2023	10/19/2023	Montana	Park 01	Backyard	WOAH NP	3	No depopulation planned.	$0.00		
10/17/2023	10/19/2023	South Dakota	Spink 09	Commercial Turkey Meat Bird	Turkey	34,007	Depopulation completed 10/19 (VSD+&Foam)	$855,626.51	11/27/2023	
10/17/2023	10/19/2023	Minnesota	Becker 05	Commercial Breeder Operation	Turkey	20,415	Depopulation completed 10/18 (Foam & KEDS)	$1,335,821.34	11/18/2023	
10/16/2023	10/18/2023	Minnesota	Redwood 02	Commercial Poultry Slaughter	Turkey	8,853	Depopulation completed 10/17 (Foam)	$324,271.29	11/4/2023	
10/16/2023	10/18/2023	Minnesota	Blue Earth 03	Commercial Turkey Meat Bird	Turkey	12,261	Depopulation completed 10/17 (Foam & KEDS)	$446,177.79	11/18/2023	
10/16/2023	10/17/2023	Washington	King 08	Backyard	WOAH NP	14	Depopulation completed 10/17 (CO2)	$123.26		

10/12/2023	10/16/2023	Oklahoma	Carter 01	Backyard	WOAH NP	76	Depopulation completed 10/13 (CO2 Cart/Container)	$2,500.00		
10/14/2023	10/17/2023	South Dakota	McPherson 06	Commercial Turkey Meat Bird	Turkey	65,002	Depopulation completed 10/16 (VSD+ & Foam)	$1,645,994.08	11/7/2023	
10/15/2023	10/17/2023	Minnesota	Becker 04	Commercial Turkey Breeder Toms	Turkey	1,595	Depopulation completed 10/15 (Foam/mechanically assisted cervical dislocation)	$354,869.58	11/10/2023	
10/13/2023	10/17/2023	South Dakota	Faulk 03	Commercial Turkey Meat Bird	Turkey	49,651	Depopulation completed 10/14 (VSD+ & Foam)	$1,290,059.95	11/7/2023	
10/13/2023	10/13/2023	North Dakota	Golden Valley 01	Backyard	WOAH NP	92	No depopulation planned.	$0.00		
10/11/2023	10/12/2023	North Dakota	Williams 01	Backyard	WOAH NP	10	No depopulation occurred. All birds died.	$0.00		
10/11/2023	10/13/2023	Montana	Flathead 03	Backyard	WOAH NP	8	Depopulation completed 10/13 (CO2)	$113.54		
10/11/2023	10/12/2023	Colorado	Weld 09	Backyard	WOAH NP	31	Depopulation completed 10/13 (CO2)	$369.14		
10/10/2023	10/13/2023	Minnesota	Meeker 10	Commercial Turkey Meat Bird	Turkey	71,021	Depopulation completed 10/11 (Foam)	$821,712.97	11/3/2023	
10/8/2023	10/11/2023	Minnesota	Meeker 09	Commercial Turkey Meat Bird	Turkey	114,981	Depopulation completed 10/9 (VSD+ & Foam)	$4,274,333.01	11/7/2023	
10/8/2023	10/10/2023	Montana	Glacier 03	Backyard	Poultry	77,069	Depopulation starts 10/10 (CO2 Whole House)	$712,783.01	11/23/2023	
10/5/2023	10/6/2023	Utah	Sanpete 20	Commercial Turkey Meat Bird	Turkey	7,559	Depopulation completed 10/7 (VSD+)	$275,072.01		
10/5/2023	10/6/2023	Utah	Sanpete 19	Commercial Turkey Meat Bird	Turkey	134,154	Depopulation completed 10/7 (VSD+)	$3,184,578.38		
Sample sent directly to NVSL	10/3/2023	Idaho	Canyon 11	Backyard	WOAH NP	70	No depopulation, 120-day quarantine of remaining birds and products.	$0.00		
10/2/2023	10/4/2023	South Dakota	Jerauld 02	Commercial Turkey Meat Bird	Turkey	47,283	Depopulation to completed 10/4 (VSD+ Foam)	$1,216,692	10/28/2023	This is a previously affected premises (Jerauld 01).
9/14/2023	9/15/2023	New Jersey	Union 01	Backyard/Live Bird Market	Multiple Poultry Species	522	Depopulation completed 9/16 (Cervical Dislocation)	$1,680		
8/2/2023	8/3/2023	New York	Kings 05	Backyard/Live Bird Market	Multiple Poultry Species	804	Depopulation completed on 8/5 (CO2 & Exsanguination)	$8,655		

7/26/2023	7/28/2023	New York	Kings 04	Backyard/Live Bird Market	Multiple Poultry Species	486	Depopulation completed 7/30 (Exsanguination)	$4,480		
7/22/2023	7/25/2023	New York	Kings 03	Backyard/Live Bird Market	Multiple Poultry Species	569	Depopulation completed 7/27 (CO_2 & Exsanguination)	$5,471		
5/16/2023	5/18/2023	North Carolina	Rowan 02	Backyard	WOAH NP	25	Depopulation completed 5/17 (Cervical dislocation)	$580		
5/16/2023	5/18/2023	Missouri	Phelps 01	Backyard	WOAH NP	82	Depopulation completed 5/17 (CO_2)	$0		This producer is not pursuing indemnity.
5/9/2023	5/11/2023	Minnesota	Nobles 01	Backyard	WOAH NP	96	Depopulation completed 5/10 (Mechanically assisted cervical dislocation)	$0		This producer is not pursuing indemnity.
5/3/2023	5/5/2023	Indiana	Posey 01	Backyard	WOAH NP	23	No depopulation occurred. All birds died.	$160		
4/21/2023	4/24/2023	Colorado	Routt 01	Backyard	WOAH NP	19	No depopulation planned.	$0		This producer is not pursuing indemnity.
Sample sent directly to NVSL	4/17/2023	Idaho	Bonneville 01	Backyard	WOAH NP	36	Depopulation completed 4/19 (CO_2)	$653		
4/17/2023	4/19/2023	North Dakota	Dickey 03	Commercial Turkey Meat Bird	Turkey	58,450	Depopulation completed 4/19 (VSD+ heat/foam)	$1,506,599	6/1/2023	This is a previously affected premises (Dickey 02).
Sample sent directly to NVSL	4/17/2023	New York	Putnam 02	Backyard	Poultry	111	Depopulation completed 4/20 (CO_2)	$0	5/4/2023	The producer is not pursuing indemnity.
4/17/2023	4/19/2023	South Dakota	Beadle 09	Commercial Turkey Meat Bird	Turkey	75,847	Depopulation completed 4/19 (VSD+ heat/foam)	$1,976,060	5/13/2023	This is a previously affected premises (Beadle 04 and Beadle 05). This is the second reinfection.
4/14/2023	4/18/2023	New York	Kings 02	Backyard/Live Bird Market	Poultry	1,357	Depopulation completed 4/19 (Humane/controlled slaughter)	$10,926		
4/14/2023	4/17/2023	Colorado	Moffat 02	Backyard	WOAH NP	80	Depopulation completed 4/18 (CO_2)	$1,035		
4/13/2023	4/14/2023	Nebraska	Sheridan 01	Backyard	WOAH NP	10	Depopulation completed 4/14 (Injectable)	$581		
Sample sent directly to NVSL	4/12/2023	California	Modoc 01	Backyard	WOAH NP	62	No depopulation planned.	$0		

Sample sent directly to NVSL	4/12/2023	New York	Putnum 01	Backyard	Poultry	200	Depopulation completed 4/14 (CO2)	$3,223	4/28/2023	
4/12/2023	4/14/2023	Montana	Rosebud 01	Backyard	WOAH NP	0	No depopulation occurred. All birds died.	$0		
4/10/2023	4/12/2023	Colorado	Pueblo 01	Backyard	WOAH NP	25	Depopulation completed 4/11 (Cervical dislocation)	$0		The producer is not pursuing indemnity.
4/7/2023	4/10/2023	Kansas	Mitchell 04	Backyard	WOAH NP	26	Depopulation completed 4/8 (CO2)	$641		
4/3/2023	4/5/2023	New York	Queens 03	Backyard/Live Bird Market	Poultry	1,255	Depopulation completed 4/6 (Humane euthanasia/CO2)	$3,657		
3/31/2023	4/3/2023	Minnesota	Le Sueur 04	Backyard	WOAH NP	97	Depopulation completed 4/2 (CO2)	$1,119		
3/28/2023	3/30/2023	Oregon	Umatilla 02	Backyard	WOAH NP	48	Depopulation completed 3/24 (CO2)	$220		
3/24/2023	3/28/2023	Colorado	Yuma 01	Backyard	Poultry	314	Depopulation completed 3/28 (CO2)	$6,256	4/11/2023	
3/23/2023	3/24/2023	Colorado	Arapahoe 01	Backyard	WOAH NP	10	Depopulation completed 3/25 (CO2)	$0		The producer is not pursuing indemnity.
3/22/2023	3/24/2023	Kansas	Ellsworth 01	Backyard	WOAH NP	47	Depopulation completed 3/23 (Cervical dislocation)	$921		
3/21/2023	3/22/2023	New York	Tompkins 01	Backyard	Poultry	6,481	Depopulation completed 3/24 (CO2)	$0	4/7/2023	The producer is not pursuing indemnity.
3/17/2023	3/22/2023	New York	Queens 02	Backyard/Live Bird Market	Poultry	139	Depopulation completed 3/23 (Humane controlled slaughter)	$871		
3/22/2023	3/23/2023	Michigan	Lapeer 05	Backyard	Poultry	947	Depopulation completed 3/23 (CO2)	$16,610	4/27/2023	This is a previously affected premises (Lapeer 03).
3/21/2023	3/22/2023	Texas	Hale 01	Backyard	WOAH NP	20	No depopulation occurred. All birds died.	$0		
Sample sent directly to NVSL	3/21/2023	New York	Washington 01	Backyard	WOAH NP	46	Depopulation completed 3/24 (CO2)	$1,160		
3/21/2023	3/22/2023	Oregon	Klamath 01	Backyard	WOAH NP	25	Depopulation completed 3/21 (CO2)	$121		
3/20/2023	3/22/2023	South Dakota	Spink 08	Commercial Upland Gamebird Producer	Pheasant	569	Depopulation completed 3/21 (Cervical dislocation)	$9,980	4/6/2023	
3/17/2023	3/21/2023	Florida	Hillsborough 04	Backyard/Live Bird Market	Chicken	340	Depopulation completed 3/20 (CO2)	$9,731	4/10/2023	
3/15/2023	3/17/2023	Pennsylvania	Lancaster 32	Backyard	Poultry	2,509	Depopulation completed 3/17 (Foam/cervical dislocation)	$450	4/19/2023	

3/15/2023	3/16/2023	Mississippi	Monroe 01	Backyard	WOAH NP	33	Depopulation completed 3/16 (Cervical dislocation)	$0		
3/14/2023	3/16/2023	Pennsylvania	Lancaster 31	Backyard	Poultry	2,359	Depopulation completed 3/15 (Foam)	$0	4/7/2023	The producer is not pursuing indemnity.
3/14/2023	3/16/2023	Pennsylvania	Lancaster 30	Backyard	Poultry	1,600	Depopulation completed 3/14 (Owner depopulation)	$0	4/8/2023	The producer is not pursuing indemnity.
3/14/2023	3/16/2023	Missouri	Maries 01	Backyard	WOAH NP	14	Depopulation completed 3/15 (CO2)	$292		
3/14/2023	3/15/2023	Pennsylvania	Mifflin 01	Backyard	WOAH NP	59	Depopulation completed 3/15 (Mechanically assisted cervical dislocation)	$335	3/31/2023	
3/13/2023	3/15/2023	Iowa	Chickasaw 01	Backyard	WOAH NP	46	Depopulation completed 3/14 (CO2)	$448		
3/13/2023	3/16/2023	Pennsylvania	Chester 04	Commercial Turkey Meat Bird	Turkey	11,800	Depopulation completed 3/15 (Foam/mechanically assisted cervical dislocation)	$157,610	4/18/2023	
3/11/2023	3/14/2023	Pennsylvania	Lancaster 29	Backyard	Poultry	3,000	Depopulation completed 3/13 (Foam)	$5,210	4/9/2023	
3/11/2023	3/14/2023	Pennsylvania	Lancaster 28	Backyard	Poultry	2,410	Depopulation completed 3/13 (Foam)	$13,496	4/12/2023	
3/11/2023	3/14/2023	Pennsylvania	Lancaster 27	Backyard	Poultry	2,957	Depopulation completed 3/13 (Foam)	$8,676	4/18/2023	
3/11/2023	3/14/2023	Pennsylvania	Lancaster 26	Backyard	Poultry	3,930	Depopulation completed 3/13 (Foam)	$3,840	4/18/2023	
3/10/2023	3/14/2023	Pennsylvania	Lancaster 25	Backyard	Poultry	2,400	Depopulation completed 3/13 (Foam)	$0	4/13/2023	The producer is not pursuing indemnity.
3/10/2023	3/14/2023	Michigan	Eaton 01	Backyard	WOAH NP	15	No depopulation occurred. All birds died.	$101		
3/9/2023	3/13/2023	Pennsylvania	Lancaster 24	Backyard	Poultry	6,020	Depopulation completed 3/10 (Foam/cervical dislocation)	$0	4/6/2023	The producer is not pursuing indemnity.
Sample sent directly to NVSL	3/6/2023	Idaho	Bingham 01	Backyard	WOAH NP	14	No depopulation occurred. All birds died.	$120		
3/6/2023	3/7/2023	Virginia	Rockingham 03	Backyard	WOAH NP	250	Depopulation completed 3/6 (CO2)	$3,955		
Sample sent directly to NVSL	3/3/2023	Nevada	Douglas 01	Backyard	WOAH NP	19	Depopulation completed 3/2 (Cervical dislocation)	$828		
3/3/2023	3/6/2023	Pennsylvania	Bucks 01	Backyard	Poultry	1,708	Depopulation completed 3/6 (Owner depopulation)	$9,570	3/31/2023	
3/2/2023	3/6/2023	Pennsylvania	Lancaster 23	Backyard	Poultry	312	Depopulation completed 3/6 (Mechanically assisted cervical dislocation)	$12,669	4/3/2023	
3/2/2023	3/6/2023	Pennsylvania	Lancaster 22	Backyard	Poultry	1,820	Depopulation completed 3/10 (Owner depopulation)	$104	4/13/2023	

3/2/2023	3/6/2023	Pennsylvania	Lancaster 21	Backyard	Poultry	6,000	Depopulation completed 3/6 (Foam)	$3,350	4/11/2023	
3/2/2023	3/3/2023	Pennsylvania	Northumberland 01	Backyard	Poultry	679	Depopulation completed 3/4 (Mechanically assisted cervical dislocation/gunshot)	$3,975	4/4/2023	
3/2/2023	3/3/2023	Pennsylvania	Lancaster 20	Commercial Duck Breeder	Duck	11,271	Depopulation completed 3/8 (Foam)	$52,578	4/16/2023	
3/2/2023	3/3/2023	Pennsylvania	Chester 03	Backyard	Poultry	5,200	Depopulation completed 3/5 (Foam)	$13,140	4/9/2023	
3/2/2023	3/3/2023	Pennsylvania	Chester 02	Backyard	Poultry	6,900	Depopulation completed 3/5 (Foam)	$32,720	4/11/2023	
3/1/2023	3/3/2023	Pennsylvania	Lancaster 19	Commercial Turkey Meat Bird	Turkey	26,601	Depopulation completed 3/4 (Foam)	$388,297	4/18/2023	
3/1/2023	3/3/2023	Pennsylvania	Lancaster 18	Commercial Turkey Meat Bird	Turkey	19,568	Depopulation completed 3/3 (Foam)	$314,934	4/7/2023	
2/24/2023	2/27/2023	Pennsylvania	Lancaster 17	Backyard	Poultry	4,371	Depopulation completed 2/25 (Foam)	$50,765	4/18/2023	
2/24/2023	2/28/2023	Florida	Miami Dade 01	Backyard/Live Bird Market	Poultry	505	Depopulation completed 2/27 (CO2)	$10,731	3/17/2023	
2/22/2023	2/23/2023	Colorado	Moffat 01	Backyard	WOAH NP	41	Depopulation completed 2/25 (CO2/cervical dislocation)	$1,485		
2/23/2023	2/24/2023	Pennsylvania	Chester 01	Backyard	Poultry	3,370	Depopulation completed 2/25 (Foam/cervical dislocation)	$13,909	4/13/2023	
2/22/2023	2/24/2023	Illinois	Wayne 01	Commercial Turkey Meat Bird	Turkey	18,200	Depopulation completed 2/23 (Foam)	$541,800	3/22/2023	
2/22/2023	2/23/2023	Virginia	Alexandria 01	Backyard/Live Bird Market	Poultry	800	Depopulation completed 2/23 (CO2)	$4,123		
2/22/2023	2/24/2023	Pennsylvania	Lancaster 16	Backyard	Poultry	4,006	Depopulation completed 2/23 (Foam)	$3,617	3/22/2023	
2/21/2023	2/23/2023	Pennsylvania	Lancaster 15	Backyard	Poultry	2,002	Depopulation completed 2/22 (Foam)	$0	4/14/2023	The producer is not pursuing indemnity.
2/21/2023	2/22/2023	Pennsylvania	Lancaster 14	Backyard	Poultry	4,200	Depopulation completed 2/21 (Foam/mechanically assisted cervical dislocation)	$11,630	3/26/2023	
2/19/2023	2/21/2023	Pennsylvania	Lancaster 13	Commercial Broiler Production	Chicken	97,700	Depopulation completed 2/20 (Foam)	$386,892	3/31/2023	
2/18/2023	2/21/2023	Nebraska	Lincoln 01	Backyard	Poultry	417	Depopulation completed 2/19 (Injectable)	$5,700	3/9/2023	
2/16/2023	2/17/2023	Mississippi	Copiah 01	Backyard	Poultry	52	Depopulation completed 2/21 (CO2)	$0	3/8/2023	The producer is not pursuing indemnity.
2/15/2023	2/17/2023	California	San Joaquin 03	Backyard	WOAH NP	164	No depopulation planned.	$0		Owner is not pursuing indemnity.

2/15/2023	2/16/2023	New York	Columbia 01	Backyard	Poultry	250	No depopulation occurred. All birds died.	$0	3/3/2023	The producer is not pursuing indemnity.
2/14/2023	2/17/2023	Pennsylvania	Tioga 01	Backyard	WOAH NP	53	Depopulation completed 2/16 (CO2)	$275	3/4/2023	
2/9/2023	2/13/2023	Kansas	Doniphan 01	Backyard	WOAH NP	30	No depopulation planned.	$0		Owner is not pursuing indemnity.
2/13/2023	2/14/2023	Florida	Hillsborough 03	Backyard	WOAH NP	10	Depopulation completed 2/17 (CO2)	$1,055		
2/10/2023	2/13/2023	Missouri	Carroll 01	Backyard	WOAH NP	9	Depopulation completed 2/11 (Cervical dislocation)	$200		
2/10/2023	2/14/2023	Washington	Benton 01	Backyard	WOAH NP	59	Depopulation completed 2/10 (CO2)	$823		
2/7/2023	2/8/2023	Kansas	Mitchell 03	Commercial Upland Gamebird Producer	Pheasant	16,950	Depopulation completed 2/10 (CO2)	$798,589	3/1/2023	
2/7/2023	2/8/2023	Pennsylvania	Lancaster 12	Backyard	Poultry	20,265	Depopulation completed 2/8 (Foam/cervical dislocation)	$63,170	3/4/2023	
	No Testing - Dangerous Contact	Mississippi	Leake 02	Commercial Broiler Production	Chicken	65,080	Depopulation completed 2/8 (Foam/CO2)	$136,017		
Sample sent directly to NVSL	2/6/2023	Wyoming	Park 03	Backyard	WOAH NP	12	No depopulation occurred. All birds died.	$0		The producer is not pursuing indemnity.
2/6/2023	2/7/2023	Mississippi	Leake 01	Commercial Broiler Production	Chicken	89,799	Depopulation completed 2/7 (Foam/CO2)	$187,680	3/2/2023	
	No Testing - Dangerous Contact	New York	Kings 01	Backyard/Live Bird Market	Poultry		No depopulation planned.	$0		Owner is not pursuing indemnity.
2/2/2023	2/6/2023	Maine	Kennebec 01	Backyard	WOAH NP	38	Depopulation completed 2/3 (CO2)	$925		
2/1/2023	2/6/2023	Maine	Hancock 02	Backyard	WOAH NP	33	Depopulation completed 2/6 (CO2)	$686		
1/31/2023	2/1/2023	Pennsylvania	Lancaster 11	Backyard	Poultry	2,700	Depopulation completed 2/1 (Foam)	$2,710	3/5/2023	
1/30/2023	2/1/2023	Pennsylvania	Lancaster 10	Commercial Duck Meat Bird	Duck	32,818	Depopulation completed 1/31 (VSD+ heat)	$29,536	3/4/2023	
1/30/2023	2/7/2023	Pennsylvania	Allegheny 02	Backyard	WOAH NP	17	No depopulation occurred. All birds died.	$0	2/22/2023	The producer is not pursuing indemnity.
1/27/2023	2/6/2023	California	San Joaquin 02	Backyard	WOAH NP	8	No depopulation planned.	$0		Owner is not pursuing indemnity.
1/27/2023	2/6/2023	California	Placer 01	Backyard	WOAH NP	25	No depopulation planned.	$0		Owner is not pursuing indemnity.

1/28/2023	2/6/2023	California	Merced 01	Commercial Duck Breeder	Duck	29,050	Depopulation completed 2/5 (CO2)	$157,433	2/23/2023	
1/26/2023	1/27/2023	Maine	Hancock 01	Backyard	WOAH NP	38	Depopulation completed 1/27 (CO2)	$1,035		
1/23/2023	1/25/2023	North Carolina	Rowan 01	Backyard	WOAH NP	2	Depopulation completed 1/25 (Injectable)	$218		
1/24/2023	1/25/2023	New York	Suffolk 04	Backyard	WOAH NP	14	Depopulation completed 1/27 (Humane Euthanasia)	$0		The producer is not pursuing indemnity.
1/24/2023	1/25/2023	Virginia	Rockingham 02	Commercial Turkey Meat Bird	Turkey	11,112	Depopulation completed 1/25 (Foam)	$321,167	2/16/2023	
1/24/2023	1/25/2023	Iowa	Buena Vista 07	Commercial Turkey Meat Bird	Turkey	27,735	Depopulation completed 1/23 (CO2)	$434,753	2/18/2023	
1/20/2023	1/25/2023	Oregon	Polk 04	Backyard	WOAH NP	18	Depopulation completed 1/23 (Cervical dislocation)	$340		
1/20/2023	1/23/2023	New Hampshire	Belknap 01	Backyard	WOAH NP	39	Depopulation completed 1/23 (Cervical dislocation)	$1,142		
1/10/2023	1/11/2023	Oregon	Douglas 02	Backyard	WOAH NP	0	No depopulation planned.	$0		Owner is not pursuing indemnity.
1/19/2023	1/20/2023	Colorado	Larimer 03	Backyard	WOAH NP	71	Depopulation completed 1/20 (CO2)	$1,830		
1/19/2023	1/20/2023	Tennessee	Weakley 05	Commercial Broiler Production	Chicken	267,753	Depopulation completed 1/20 (VSD+ heat)	$835,820	2/12/2023	
1/18/2023	1/19/2023	California	El Dorado 02	Backyard	WOAH NP	16	No depopulation planned.	$4,596		
1/18/2023	1/19/2023	Washington	Snohomish 09	Backyard	WOAH NP	15	No depopulation occurred. All birds died.	$0		No depopulation planned. The producer is not pursuing indemnity.
1/17/2023	1/18/2023	Texas	Lampasas 01	Backyard	Poultry	66	Depopulation completed 1/18 (CO2)	$1,875	2/2/2023	
1/18/2023	1/19/2023	Virginia	Rockingham 01	Commercial Turkey Meat Bird	Turkey	25,264	Depopulation completed 1/20 (Foam)	$760,445	2/16/2023	
1/16/2023	1/17/2023	Kansas	Mitchell 02	Commercial Upland Gamebird Producer	Pheasant	6,878	Depopulation completed 1/20 (CO2)	$120,640	3/1/2023	
1/13/2023	1/18/2023	Ohio	Licking 01	Backyard	WOAH NP	30	Depopulation completed 1/14 (CO2)	$250		
1/11/2023	1/12/2023	California	Tehama 01	Backyard	Poultry	23,730	Depopulation completed 1/13 (CO2)	$82,921	2/13/2023	

Sample sent directly to NVSL	1/9/2023	California	Sonoma 02	Backyard	WOAH NP	6	No depopulation planned.	$0		Owner is not pursuing indemnity.
1/6/2023	1/10/2023	Montana	Cascade 02	Backyard	WOAH NP	12	Depopulation completed 1/6 (Gunshot)	$240		
1/6/2023	1/9/2023	Nebraska	Scotts Bluff 02	Backyard	WOAH NP	7	Depopulation completed 1/7 (Injectable/ barbiturate/ cervical dislocation	$90		
1/5/2023	1/6/2023	Colorado	Weld 08	Backyard	WOAH NP	35	Depopulation completed 1/6 (CO_2)	$835		
1/4/2023	1/6/2023	Washington	Thurston 02	Backyard	WOAH NP	116	Depopulation completed 1/5 (CO_2)	$3,100		
1/4/2023	1/9/2023	California	Sonoma 01	Backyard	WOAH NP	15	No depopulation occurred. All birds died.	$0		
1/4/2023	1/9/2023	California	San Joaquin 01	Backyard	WOAH NP	11	No depopulation planned.	$0		Owner is not pursuing indemnity.
1/4/2023	1/4/2023	Missouri	Johnson 01	Backyard	WOAH NP	25	Depopulation completed 1/5 (CO_2/mechanically assisted cervical dislocation)	$990		
1/3/2023	1/4/2023	Kansas	Anderson 01	Commercial Upland Gamebird Producer	Pheasant	8,936	Depopulation completed 1/5 (Foam)	$146,740	1/24/2023	
12/30/2022	1/5/2023	South Dakota	Codington 02	Backyard	Poultry	140	No depopulation occurred. All birds died.	$285	1/18/2023	
12/30/2022	1/4/2023	Oregon	Josephine 01	Backyard	WOAH NP	30	Depopulation completed 12/30 (CO_2)	$860		
12/28/2022	12/30/2022	Washington	Snohomish 08	Backyard	Poultry	178	Depopulation completed 12/29 (CO_2)	$4,650	1/31/2023	
12/28/2022	12/28/2022	Kansas	Elk 01	Backyard	WOAH NP	25	Depopulation completed 12/28 (Cervical dislocation)	$0		Producer is not pursuing indemnity.
12/20/2022	12/21/2022	Illinois	St. Clair 01	Pet Bird	WOAH NP	58	No depopulation planned.	$0		Owner is not pursuing indemnity.

12/26/2022	12/28/2022	Tennessee	Weakley 03	Commercial Broiler Breeder	Chicken	43,635	Depopulation completed 12/28 (Foam)	$227,340	2/1/2023	
12/26/2022	12/28/2022	Tennessee	Weakley 04	Commercial Broiler Breeder	Chicken	18,980	Depopulation completed 12/28 (Foam)	$98,890	2/1/2023	
12/22/2022	12/30/2022	Michigan	Sanilac 01	Backyard	WOAH NP	32	Depopulation completed 12/26 (Injectable)	$975	1/10/2023	
12/20/2022	12/22/2022	California	Glenn 01	Commercial Upland Gamebird Producer	Pheasant	32,000	Depopulation completed 1/5 (CO2)	$389,378	2/13/2023	
12/20/2022	12/29/2022	Washington	Pierce 06	Backyard	WOAH NP	35	Depopulation completed 12/21 (CO2)	$0		Owner is not pursuing indemnity.
12/19/2022	12/21/2022	Missouri	Bates 02	Backyard	WOAH NP	28	Depopulation completed 12/21 (CO2)	$785		
12/19/2022	12/20/2022	Idaho	Gem 01	Backyard	WOAH NP	83	No depopulation planned.	$0		Owner is not pursuing indemnity.
12/19/2022	12/21/2022	Oregon	Umatilla 01	Backyard	WOAH NP	14	Depopulation completed 12/20 (CO2)	$220		
12/20/2022	12/21/2022	Tennessee	Weakley 02	Backyard	Poultry	28,766	Depopulation completed 12/22 (CO2/cervical dislocation)	$41,425	2/1/2023	
12/17/2022	12/20/2022	Colorado	Weld 07	Commercial Table Egg Layer	Chicken	239,657	Depopulation completed 12/31 (CO2)	$1,053,941	1/28/2023	
12/16/2022	12/19/2022	California	Mendocino 01	Backyard	WOAH NP	20	No depopulation planned.	$0		Owner is not pursuing indemnity.
12/14/2022	12/16/2022	California	Butte 02	Backyard	WOAH NP	23	Depopulation completed 12/17 (Cervical dislocation)	$0		
12/15/2022	12/16/2022	Montana	Flathead 02	Backyard	WOAH NP	0	No depopulation occurred. All birds died.	$0		Owner is not pursuing indemnity.
12/14/2022	12/20/2022	South Dakota	Hanson 05	Commercial Turkey Meat Bird	Turkey	31,800	Depopulation completed 12/9 (VSD+ heat/foam)	$855,543	1/20/2023	
12/15/2022	12/16/2022	Florida	Duval 02	Backyard	WOAH NP	11	Depopulation completed 12/15 (CO2)	$585		
12/14/2022	12/16/2022	Colorado	Weld 06	Commercial Table Egg Layer	Chicken	1,290,966	Depopulation completed 1/2 (CO2)	$7,374,801	1/23/2023	This is a previously affected premises (Weld 02).

	No Testing - Dangerous Contact	Indiana	Martin 01	Commercial Turkey Meat Bird	Turkey	27,083	Depopulation completed 12/12 (Foam)	$457,058		
12/13/2022	12/15/2022	Oregon	Polk 03	Backyard	WOAH NP	24	Depopulation completed 12/14 (CO2)	$200		
12/13/2022	12/14/2022	Nebraska	Knox 03	Backyard	Poultry	156	Depopulation completed 12/16 (Cervical dislocation/gunshot)	$3,008	1/13/2023	
12/11/2022	12/13/2022	Indiana	Daviess 01	Commercial Turkey Meat Bird	Turkey	11,394	Depopulation completed 12/11 (Foam)	$342,959	1/5/2023	
12/12/2022	12/14/2022	Tennessee	Weakley 01	Backyard	Poultry	37,874	Depopulation completed 12/16 (Foam/cervical dislocation)	$217,397	2/1/2023	
12/11/2022	12/12/2022	Iowa	Ida 01	Commercial Turkey Meat Bird	Turkey	90,000	Depopulation completed 12/13 (VSD+ heat)	$2,709,000	1/26/2023	
12/10/2022	12/12/2022	Iowa	Cherokee 03	Commercial Turkey Meat Bird	Turkey	50,000	Depopulation completed 12/13 (VSD+ heat)	$1,505,000	1/14/2023	
12/10/2022	12/12/2022	Iowa	Buena Vista 06	Commercial Turkey Meat Bird	Turkey	51,200	Depopulation completed 12/12 (VSD+ heat)	$1,231,000	1/17/2023	
12/9/2022	12/14/2022	Minnesota	Redwood 01	Backyard	WOAH NP	41	Depopulation completed 12/11 (Mechanically cervical dislocation)	$1,237		
12/10/2022	12/14/2022	South Dakota	Moody 01	Commercial Table Egg Layer	Chicken	1,332,077	Depopulation completed 12/15 (VSD+ heat/CO2)	$4,149,035	1/19/2023	
12/9/2022	12/14/2022	South Dakota	Spink 07	Backyard	Poultry	280	Depopulation completed 12/10 (Cervical dislocation)	$5,559	1/5/2023	
12/9/2022	12/13/2022	Oregon	Linn 03	Backyard	WOAH NP	15	Depopulation completed 12/12 (CO2)	$260		
12/9/2022	12/14/2022	Washington	Franklin 01	Commercial Table Egg Layer	Chicken	1,015,476	Depopulation completed 12/19 (CO2/cervical dislocation)	$2,436,385	1/31/2023	
12/9/2022	12/9/2022	Colorado	Prowers 01	Commercial Upland Gamebird Producer	Pheasant	11,043	Depopulation completed 12/12 (CO2)	$100,470	12/28/2023	
12/8/2022	12/12/2022	Iowa	Sac 03	Commercial Turkey Meat Bird	Turkey	50,000	Depopulation completed 12/10 (VSD+ heat)	$1,505,000	1/15/2023	

12/8/2022	12/13/2022	Oregon	Columbia 02	Backyard	WOAH NP	103	Depopulation completed 12/9 (CO2)	$1,135		
12/8/2022	12/12/2022	South Dakota	Hanson 04	Commercial Turkey Meat Bird	Turkey	23,910	Depopulation completed 12/9 (VSD+ heat/foam)	$515,050	1/3/2023	
12/8/2022	12/9/2022	North Carolina	Onslow 01	Backyard	WOAH NP	55	Depopulation completed 12/9 (Cervical dislocation)	$1,140		
12/7/2022	12/9/2022	Oregon	Polk 02	Backyard	WOAH NP	71	Depopulation completed 12/8 (CO2/firearm)	$2,288		
12/7/2022	12/9/2022	Missouri	Osage 01	Commercial Turkey Meat Bird	Turkey	19,695	Depopulation completed 12/9 (Foam)	$312,700	1/3/2023	
12/7/2022	12/9/2022	Oregon	Clackamas 01	Backyard	WOAH NP	57	Depopulation completed 12/8 (CO2)	$2,800		
12/7/2022	12/12/2022	Minnesota	Wadena 01	Commercial Turkey Meat Bird	Turkey	298,137	(Depopulation completed 12/8 (Foam)	$3,185,943	12/29/2022	
12/7/2022	12/8/2022	Missouri	Dade 02	Backyard	WOAH NP	32	Depopulation completed 12/7 (Owner)	$0		Owner is not pursuing indemnity.
12/6/2022	12/8/2022	Minnesota	Dodge 02	Commercial Turkey Meat Bird	Turkey	9,978	Depopulation completed 12/7 (Foam)	$239,208	12/24/2022	
12/6/2022	12/8/2022	South Dakota	Hamlin 04	Commercial Turkey Meat Bird	Turkey	58,137	Depopulation completed 12/7 (VSD+ heat/foam)	$1,276,240	12/30/2022	
Sample sent directly to NVSL	12/6/2022	Vermont	Lamoille 01	Backyard	WOAH NP	53	Depopulation completed 12/8 (CO2)	$480		
12/5/2022	12/7/2022	South Dakota	Kingsbury 04	Backyard	WOAH NP	69	Depopulation completed 12/6 (Humane depopulation)	$2,240		
12/5/2022	12/7/2022	Iowa	Sac 02	Commercial Turkey Meat Bird	Turkey	40,000	Depopulation completed 12/6 (VSD+ heat)	$1,204,000	1/5/2023	
12/5/2022	12/7/2022	Iowa	Cherokee 02	Commercial Turkey Meat Bird	Turkey	105,000	Depopulation completed 12/7 (VSD+ heat)	$2,585,100	1/14/2023	This is a previously affected premises (Cherokee 01).
12/1/2022	12/6/2022	Colorado	Larimer 02	Backyard	WOAH NP	2	No depopulation occurred. All birds died.	$0		Owner is not pursuing indemnity.
12/2/2022	12/6/2022	Oklahoma	Pawnee 01	Backyard	WOAH NP	25	Depopulation completed 12/5 (CO2)	$795		

12/3/2022	12/6/2022	Missouri	Harrison 01	Backyard	WOAH NP	32	Depopulation completed 12/4 (Cervical dislocation)	$240		
12/2/2022	12/6/2022	Texas	Denton 01	Backyard	WOAH NP	38	No depopulation occurred. All birds died.	$2,510		
12/1/2022	12/5/2022	Alabama	Lawrence 01	Backyard	WOAH NP	463	Depopulation completed 12/3 (Cervical dislocation)	$5,834		
12/1/2022	12/5/2022	South Dakota	Clark 07	Commercial Turkey Meat Bird	Turkey	24,301	Depopulation completed 12/2 (VSD+ heat/foam)	$438,066	12/27/2022	This is a previously affected premises (Clark 03).
12/1/2022	12/5/2022	South Dakota	Charles Mix 06	Commercial Turkey Meat Bird	Turkey	34,100	Depopulation completed 12/3 (VSD+ heat/foam)	$734,051	12/20/2022	This is a previously affected premises (Charles Mix 01).
12/1/2022	12/2/2022	North Carolina	Union 02	Backyard	WOAH NP	9	Depopulation completed 12/2 (Cervical dislocation)	$280		
12/1/2022	12/5/2022	Iowa	Buena Vista 05	Commercial Turkey Meat Bird	Turkey	40,681	Depopulation completed 12/2 (VSD+ heat)	$3,000,519	12/27/2022	
11/30/2022	12/2/2022	South Dakota	Clark 06	Backyard	WOAH NP	39	Depopulation completed 12/1 (Cervical dislocation)	$670		
11/30/2022	12/1/2022	South Dakota	Brookings 01	Backyard	WOAH NP	205	Depopulation completed 12/1 (Cervical dislocation)	$110		
11/30/2022	12/2/2022	Nebraska	Knox 02	Backyard	WOAH NP	12	Depopulation completed 11/30 (Depopulated by Owner)	$0		Owner is not pursuing indemnity.
11/30/2022	12/1/2022	Arkansas	Arkansas 01	Backyard	WOAH NP	24	No depopulation occurred. All birds died.	$0		Owner is not pursuing indemnity.
11/29/2022	12/1/2022	Missouri	Jackson 02	Backyard	WOAH NP	25	Depopulation completed 11/30 (Cervical dislocation)	$0		Owner is not pursuing indemnity.
11/30/2022	12/1/2022	South Dakota	Lake 02	Commercial Turkey Meat Bird	Turkey	78,000	Depopulation completed 12/1 (VSD+ heat/foam)	$1,825,200	12/27/2022	This is a previously affected premises (Lake 01).
11/29/2022	11/30/2022	Illinois	Grundy 01	Backyard	Poultry	8,825	Depopulation completed 12/1 (Cervical dislocation)	$154,532	12/30/2022	
11/28/2022	11/30/2022	South Dakota	Hamlin 03	Commercial Turkey Meat Bird	Turkey	49,987	Depopulation completed 11/30 (VSD+ heat/foam)	$1,160,336	12/19/2022	

11/28/2022	11/30/2022	South Dakota	Charles Mix 05	Commercial Turkey Meat Bird	Turkey	75,089	Depopulation completed 11/30 (VSD+ heat/foam)	$1,597,272	12/19/2022	This is a previously affected premises (Charles Mix 02).
11/27/2022	11/29/2022	Maryland	Washington 01	Commercial Table Egg Breeder	Chicken	24,600	Depopulation completed 11/29 (VSD + heat)	$66,010	12/22/2022	
Sample sent directly to NVSL	11/25/2022	Nebraska	Dixon 02	Commercial Table Egg Layer	Chicken	1,700,000	Depopulation completed 12/4 (VSD+ heat/CO2)	$6,943,175	12/30/2022	
11/25/2022	11/29/2022	South Dakota	Hanson 03	Commercial Turkey Meat Bird	Turkey	71,550	Depopulation completed 11/28 (VSD+ heat/foam)	$1,561,761	12/20/2022	This is a previously affected premises (Hanson 02).
Sample sent directly to NVSL	11/25/2022	Alaska	Matanuska-Susitna 05	Backyard	WOAH NP	35	No depopulation planned.	$485		
11/23/2022	11/29/2022	South Dakota	Spink 06	Commercial Turkey Meat Bird	Turkey	32,900	Depopulation completed 11/25 (VSD+ heat/foam)	$747,651	12/19/2022	This is a previously affected premises (Spink 01).
11/23/2022	11/28/2022	Utah	Iron 01	Backyard	Poultry	3,000	Depopulation completed 11/25 (Cervical dislocation)	$58,798	12/10/2022	
11/24/2022	11/28/2022	Missouri	Webster 02	Commercial Turkey Meat Bird	Turkey	15,065	Depopulation completed 11/25 (Foam)	$454,329	12/16/2022	
11/23/2022	11/25/2022	South Dakota	Beadle 08	Commercial Turkey Meat Bird	Turkey	67,935	Depopulation completed 11/27 (VSD+ heat/foam)	$1,171,108	12/19/2022	This is a previously affected premises (Beadle 03).
11/23/2022	11/25/2022	North Carolina	Durham 01	Backyard	WOAH NP	26	Depopulation completed 11/23 (Cervical dislocation)	$545		
11/22/2022	11/25/2022	South Dakota	Spink 05	Commercial Turkey Meat Bird	Turkey	55,500	Depopulation completed 11/25 (VSD+ heat/foam)	$1,008,915	12/14/2022	This is a previously affected premises (Spink 02).
11/22/2022	11/25/2022	South Dakota	Turner 01	Backyard	WOAH NP	211	Depopulation completed 11/28 (Cervical dislocation)	$4,160		

11/22/2022	11/25/2022	South Dakota	Beadle 07	Commercial Turkey Meat Bird	Turkey	50,700	Depopulation completed 11/23 (VSD+ heat/foam)	$1,117,397	12/12/2022	This is a previously affected premises (Beadle 02).
11/22/2022	11/23/2022	Tennessee	Davidson 01	Backyard	WOAH NP	14	Depopulation completed 11/23 (Mechanically assisted cervical dislocation)	$393		
11/21/2022	11/23/2022	Oregon	Columbia 01	Backyard	WOAH NP	25	Depopulation completed 11/23 (CO2)	$390		
11/21/2022	11/23/2022	South Dakota	Brule 03	Backyard	WOAH NP	30	Depopulation completed 11/22 (Cervical dislocation)	$9,173		
11/21/2022	11/23/2022	South Dakota	Faulk 02	Commercial Turkey Meat Bird	Turkey	49,850	Depopulation completed 11/25 (VSD+ heat/foam)	$1,058,252	12/23/2023	This is a previously affected premises (Faulk 01).
11/21/2022	11/23/2022	Florida	Broward 05	Backyard	WOAH NP	11	Depopulation completed 11/22 (CO2)	$327		
11/20/2022	11/23/2022	Minnesota	Todd 08	Commercial Turkey Meat Bird	Turkey	44,500	Depopulation completed 11/21 (Foam/cervical dislocation)	$616,913	12/13/2022	
11/19/2022	11/22/2022	Missouri	Webster 01	Backyard	Poultry	9,147	Depopulation completed 11/22 (CO2)	$27,552	12/16/2022	
11/19/2022	11/22/2022	South Dakota	Beadle 06	Commercial Turkey Meat Bird	Turkey	29,200	Depopulation completed 11/21 (VSD+ heat/foam)	$626,333	12/9/2022	This is a previously affected premises (Beadle 01).
11/17/2022	11/18/2022	North Carolina	Union 01	Backyard	WOAH NP	53	Depopulation completed 11/18 (Cervical dislocation)	$1,075		
11/17/2022	11/21/2022	South Dakota	Edmunds 05	Commercial Turkey Meat Bird	Turkey	34,400	Depopulation completed 11/19 (VSD+ heat/foam)	$417,656	12/5/2022	This is a previously affected premises (Edmunds 04).
11/17/2022	11/18/2022	Utah	Utah 02	Petting Zoo/Exhibition Farm	WOAH NP	270	Depopulation completed 11/18 (Cervical dislocation/long netting)	$5,965		
11/17/2022	11/21/2022	Maine	Washington 02	Backyard	WOAH NP	91	Depopulation completed 11/18 (CO2)	$853		

11/16/2022	11/17/2022	Florida	Broward 04	Backyard	WOAH NP	16	No depopulation occurred. All birds died.	$0		Owner is not pursuing indemnity.
11/15/2022	11/16/2022	California	Fresno 07	Commercial Duck Breeder	Duck	34,734	Depopulation completed 11/19 (Foam)	$1,221,680	1/4/2023	
11/15/2022	11/17/2022	Pennsylvania	Berks 12	Backyard	WOAH NP	26	Depopulation completed 11/17 (Mechanically assisted cervical dislocation)	$449	12/3/2022	
11/14/2022	11/16/2022	Florida	Seminole 02	Backyard	Poultry	99	Depopulation completed 11/16 (CO2/mechanically assisted cervical dislocation)	$1,700	12/7/2022	
11/14/2022	11/16/2022	Florida	Hillsborough 02	Backyard	WOAH NP	100	Depopulation completed 11/15 (CO2)	$1,470	12/5/2022	
11/13/2022	11/15/2022	Minnesota	Otter Tail 07	Commercial Turkey Meat Bird	Turkey	28,244	Depopulation completed 11/14 (Foam)	$1,053,170	12/7/2022	This is a previously affected premises (Otter Tail 04).
11/11/2022	11/12/2022	New York	Queens 01	Backyard	Poultry	169	Depopulation completed 11/13 (Humane/controlled slaughter)	$287		
11/12/2022	11/15/2022	Tennessee	Bledsoe 01	Commercial Broiler Breeder	Chicken	20,892	Depopulation completed 11/15 (VSD+ heat/cervical dislocation)	$114,620	12/7/2022	
11/9/2022	11/10/2022	Pennsylvania	Lehigh 06	Commercial Turkey Breeder Toms	Turkey	155	Depopulation completed 11/10 (Mechanically assisted cervical dislocation)	$2,710	12/3/2022	
11/9/2022	11/10/2022	North Dakota	Mountrail 01	Backyard	WOAH NP	38	Depopulation completed 11/12 (Cervical dislocation)	$650	12/6/2022	
	No Testing - Dangerous Contact	Michigan	Lapeer 04	Backyard	Poultry	1,113	Depopulation completed 11/11 (CO2)	$15,030		
11/8/2022	11/14/2022	Mississippi	Lowndes 01	Pet Bird	WOAH NP	8	No depopulation planned.	$0	11/24/2022	Owner is not pursuing indemnity.
11/9/2022	11/10/2022	Michigan	Lapeer 03	Backyard	Poultry	990	Depopulation completed 11/10 (CO2)	$6,965	11/25/2022	

11/9/2022	11/10/2022	Pennsylvania	Lehigh 05	Commercial Turkey Meat Bird	Turkey	18,500	Depopulation completed 11/10 (Foam)	$2,038,856	12/3/2022	
11/8/2022	11/10/2022	North Dakota	Ward 02	Backyard	Poultry	22	Depopulation completed 11/9 (CO2)	$210	12/6/2022	
11/8/2022	11/15/2022	Florida	Duval 01	Backyard	WOAH NP	26	Depopulation completed 11/14 (CO2)	$540	11/28/2022	
11/7/2022	11/8/2022	Pennsylvania	Lehigh 04	Commercial Turkey Meat Bird	Turkey	17,400	Depopulation completed 11/8 (Foam)	$0	12/3/2022	Owner is not pursuing indemnity.
11/4/2022	11/6/2022	Iowa	Wright 02	Commercial Table Egg Layer	Chicken	1,022,773	Depopulation completed 11/8 (VSD+ heat)	$5,191,935	12/27/2022	
11/6/2022	11/8/2022	Pennsylvania	Lehigh 03	Commercial Turkey Breeder Hens	Turkey	7,500	Depopulation completed 11/7 (Mechanically assisted cervical dislocation)	$0	12/3/2022	Owner is not pursuing indemnity.
11/4/2022	11/7/2022	Virginia	Gloucester 01	Backyard	WOAH NP	76	Depopulation completed 11/5 (CO2)	$1,880	11/19/2022	
11/4/2022	11/8/2022	Iowa	Louisa 01	Backyard	WOAH NP	17	No depopulation occurred. All birds died.	$0	11/11/2022	Owner is not pursuing indemnity.
11/4/2022	11/7/2022	Wyoming	Converse 01	Backyard	WOAH NP	24	Depopulation completed 11/5 (CO2)	$590	11/28/2022	
11/4/2022	11/8/2022	Washington	Snohomish 07	Backyard	WOAH NP	148	Depopulation completed 11/5 (CO2)	$3,620	11/22/2022	
Sample sent directly to NVSL	11/3/2022	Arizona	Yavapai 02	Backyard	WOAH NP	30	Depopulation completed 11/4 (CO2)	$196	11/21/2022	
11/4/2022	11/7/2022	Wisconsin	Jefferson 02	Commercial Upland Gamebird Producer	Pheasant	187,900	Depopulation completed 11/15 (Cervical dislocation/ CO2/foam)	$2,816,220	12/16/2022	
11/3/2022	11/4/2022	New York	Sullivan 01	Backyard	WOAH NP	142	Depopulation completed 11/6 (CO2)	$3,130	11/22/2022	
11/3/2022	11/7/2022	Ohio	Butler 01	Backyard	Poultry	150	Depopulation completed 11/9 (CO2)	$1,065	11/24/2022	
11/3/2022	11/4/2022	Mississippi	Lawrence 01	Commercial Broiler Breeder	Chicken	34,386	Depopulation completed 11/4 (CO2/foam)	$179,150	12/1/2023	
11/3/2022	11/4/2022	Pennsylvania	Lehigh 02	Commercial Turkey Meat Bird	Turkey	14,500	Depopulation completed 11/5 (Foam)	$0	12/3/2022	Owner is not pursuing indemnity.

11/2/2022	11/3/2022	South Carolina	Beaufort 01	Backyard	WOAH NP	172	Depopulation completed 11/5 (CO2)	$2,285	11/20/2023	
11/1/2022	11/2/2022	Wisconsin	Waukesha 01	Backyard	WOAH NP	12	Depopulation completed 11/3 (CO2)	$180	11/17/2022	
11/1/2022	11/3/2022	Wisconsin	Marathon 01	Backyard	WOAH NP	30	Depopulation completed 11/3 (CO2)	$290	11/17/2022	
11/2/2022	11/4/2022	Pennsylvania	Dauphin 01	Backyard	WOAH NP	125	Depopulation completed 11/4 (CO2)	$2,030	12/27/2022	
11/2/2022	11/3/2022	California	San Diego 01	Backyard	Poultry	145	Depopulation completed 11/3 (CO2)	$2,670	11/19/2022	
Sample sent directly to NVSL	11/2/2022	Arizona	Yavapai 01	Backyard	WOAH NP	52	Depopulation completed 11/3 (CO2)	$1,110	11/21/2022	
11/2/2022	11/3/2022	North Dakota	Bottineau 01	Backyard	Poultry	130	Depopulation completed 11/3 (Cervical dislocation)	$1,495	12/5/2022	
11/1/2022	11/3/2022	Pennsylvania	Lehigh 01	Commercial Turkey Meat Bird	Turkey	28,500	Depopulation completed 11/2 (Foam)	$0	12/3/2022	Owner is not pursuing indemnity.
11/2/2022	11/2/2022	New Jersey	Bergen 01	Backyard	WOAH NP	55	Depopulation completed 11/3 (CO2)	$1,890	11/22/2022	
11/1/2022	11/3/2022	Oregon	Yamhill 01	Backyard	Poultry	150	Depopulation completed 11/3 (CO2)	$4,405	11/22/2022	
11/2/2022	11/3/2022	Florida	Broward 03	Backyard	WOAH NP	70	Depopulation completed 11/7 (CO2)	$1,222	11/22/2022	
11/1/2022	11/3/2022	Massachusetts	Middlesex 01	Backyard	WOAH NP	20	Depopulation completed 11/3 (CO2)	$305	11/16/2022	
11/1/2022	11/2/2022	Pennsylvania	Allegheny 01	Backyard	WOAH NP	20	Depopulation completed 11/2 (Cervical dislocation/gunshot)	$550	11/18/2022	
10/27/2022	11/1/2022	New York	Wayne 01	Backyard	WOAH NP	11	Depopulation completed 11/4 (Cervical dislocation)	$60	11/21/2022	
10/30/2022	11/1/2022	Minnesota	Stearns 13	Commercial Turkey Meat Bird	Turkey	8,491	Depopulation completed 10/31 (Foam/mechanically assisted cervical dislocation)	$610,625	11/19/2022	
10/30/2022	10/31/2022	Iowa	Wright 01	Commercial Table Egg Layer	Chicken	1,100,000	Depopulation completed 11/3 (VSD+ heat)	$2,872,060	12/2/2022	

10/28/2022	10/31/2022	Tennessee	Tipton 01	Backyard	WOAH NP	90	Depopulation completed 10/31 (CO2/mechanically assisted cervical dislocation)	$3,575	11/15/2022	
10/29/2022	11/1/2022	Oregon	Deschutes 07	Backyard	WOAH NP	8	Depopulation completed 10/31 (CO2)	$220	11/22/2022	
10/28/2022	11/1/2022	South Dakota	Gregory 02	Commercial Upland Gamebird Producer	Pheasant	3,000	Depopulation completed 11/3 (CO2/cervical dislocation)	$19,500	11/22/2022	
10/27/2022	10/31/2022	Minnesota	Swift 06	Commercial Turkey Meat Bird	Turkey	45,575	Depopulation completed 10/28 (Foam/cervical dislocation)	$512,480	11/21/2022	
10/27/2022	11/1/2022	Montana	Granite 01	Backyard	Poultry	43	Depopulation completed 10/28 (CO2)	$865	11/13/2022	
10/26/2022	10/28/2022	Florida	Hillsborough 01	Backyard	WOAH NP	43	Depopulation completed 10/31 (CO2)	$1,055	11/16/2022	
10/26/2022	10/27/2022	Minnesota	Stearns 12	Commercial Turkey Meat Bird	Turkey	75,000	Depopulation completed 10/27 (Foam/mechanically assisted cervical dislocation)	$813,650	11/17/2022	
10/25/2022	10/27/2022	Minnesota	Le Sueur 03	Commercial Turkey Meat Bird	Turkey	20,058	Depopulation completed 10/25 (Foam)	$600,800	11/29/2022	
10/24/2022	10/26/2022	Florida	Pasco 01	Backyard	WOAH NP	40	Depopulation completed 10/26 (CO2)	$370	11/11/2022	
10/24/2022	10/26/2022	Colorado	La Plata 03	Backyard	WOAH NP	20	No depopulation planned.	$330	11/17/2022	Owner refused to allow federal/state personnel on premises.
10/21/2022	10/25/2022	South Dakota	Grant 01	Backyard	WOAH NP	48	Depopulation completed 10/26 (Cervical Dislocation)	$685	11/22/2022	
10/19/2022	10/20/2022	Iowa	Dallas 01	Backyard	WOAH NP	48	Depopulation completed 10/20 (CO2/mechanically assisted cervical dislocation)	$1,255	11/4/2022	
10/23/2022	10/25/2022	Minnesota	Swift 05	Commercial Turkey Meat Bird	Turkey	33,763	Depopulation completed 10/24 (Foam)	$1,018,345	11/19/2022	
10/23/2022	10/25/2022	Utah	Sanpete 18	Commercial Turkey Meat Bird	Turkey	3,880	Depopulation completed 10/24 (VSD+ heat/mechanically assisted cervical dislocation)	$94,395	11/17/2022	

10/21/2022	10/22/2022	Virginia	Southampton 01	Backyard	WOAH NP	170	Depopulation completed 10/22 (CO2)	$5,600	11/6/2022	
10/21/2022	10/25/2022	Florida	Indian River 04	Backyard	Poultry	50	Depopulation completed 10/24 (CO2)	$1,375	11/8/2022	
10/21/2022	10/25/2022	Florida	Martin 02	Backyard	WOAH NP	34	No depopulation occurred. All birds died.	$0	11/11/2022	Owner is not pursuing indemnity.
10/21/2022	10/22/2022	New Jersey	Ocean 01	Animal Rescue / Rehabilitation	WOAH NP	93	Depopulation completed 10/25 (CO2)	$3,865	11/9/2022	
10/21/2022	10/25/2022	Florida	Indian River 03	Backyard	WOAH NP	33	No depopulation planned.	$0	11/16/2022	Owner is not pursuing indemnity.
10/20/2022	10/21/2022	Minnesota	Itasca 01	Backyard	WOAH NP	40	Depopulation completed 10/21 (CO2/mechanically assisted cervical dislocation)	$1,145	11/5/2022	
10/21/2022	10/25/2022	Florida	Lake 01	Backyard	WOAH NP	9	Depopulation completed 10/22 (CO2)	$270	11/8/2022	
10/20/2022	10/21/2022	Oklahoma	Cleveland 01	Backyard	WOAH NP	32	Depopulation completed 10/21 CO2)	$550	11/5/2022	
Sample sent directly to NVSL	10/20/2022	Virginia	Virginia Beach 01	Backyard	WOAH NP	10	No depopulation planned.	$0	11/4/2022	Owner is not pursuing indemnity.
10/19/2022	10/21/2022	North Carolina	Wake 01	Backyard	WOAH NP	172	Depopulation completed 10/21 (Cervical dislocation/long netting)	$2,980	11/8/2022	
10/19/2022	10/19/2022	Texas	Rockwall 01	Backyard	WOAH NP	46	Depopulation completed 10/20 (CO2)	$825	11/15/2022	
10/20/2022	10/20/2022	Rhode Island	Newport 01	Backyard	WOAH NP	62	Depopulation completed 10/21 (CO2)	$645	11/4/2022	
10/18/2022	10/20/2022	Oklahoma	Creek 01	Backyard	WOAH NP	47	Depopulation completed 10/17 (CO2)	$0	11/2/2022	Owner is not pursuing indemnity.
10/19/2022	10/20/2022	Idaho	Valley 01	Backyard	WOAH NP	21	Depopulation completed 10/21 (CO2)	$565	11/7/2022	
10/19/2022	10/20/2022	Montana	Hill 01	Backyard	WOAH NP	56	Depopulation completed 10/20 (Cervical dislocation)	$2,100	11/5/2022	
10/18/2022	10/20/2022	South Dakota	Spink 04	Backyard	WOAH NP	159	Depopulation completed 10/20 (CO2/cervical dislocation/gunshot)	$2,140	11/8/2022	

10/18/2022	10/20/2022	Florida	Okeechobee 01	Backyard	WOAH NP	2	Depopulation completed 10/19 (CO_2)	$0	11/2/2022	Owner is not pursuing indemnity.
10/18/2022	10/20/2022	Florida	Indian River 02	Backyard	WOAH NP	56	Depopulation completed 10/20 (CO_2)	$1,150	11/8/2022	
10/18/2022	10/20/2022	Colorado	Jefferson 02	Backyard	Poultry	50	Depopulation completed 10/19 (CO_2)	$1,205	11/3/2022	
10/18/2022	10/19/2022	Kansas	Shawnee 01	Backyard	Poultry	121	Depopulation completed 10/20 (CO_2)	$1,715	11/8/2022	
10/17/2022	10/19/2022	South Dakota	Roberts 01	Commercial Breeder Operation	Goose	3,588	Depopulation completed 10/20 (Humane/controlled slaughter)	$139,700	12/9/2022	
10/18/2022	10/19/2022	Utah	Sanpete 17	Commercial Turkey Meat Bird	Turkey	9,372	Depopulation completed 10/14 (Foam)	$229,165	11/15/2022	
Sample sent directly to NVSL	10/17/2022	Nevada	Nye 01	Backyard	Poultry	1,600	No depopulation planned.	$3,750	7/14/2023	
10/14/2022	10/18/2022	Missouri	Jackson 01	Backyard	WOAH NP	39	No depopulation planned.	$560	11/8/2022	
10/17/2022	10/18/2022	Florida	Saint Lucie 01	Backyard	WOAH NP	6	Depopulation completed 10/20 (CO_2)	$0	11/18/2022	Owner is not pursuing indemnity.
10/15/2022	10/18/2022	Nebraska	York 02	Commercial Upland Gamebird Producer	Pheasant	34,890	Depopulation completed 10/20 (CO_2/foam)	$348,900	11/16/2022	
10/15/2022	10/18/2022	Florida	Indian River 01	Backyard	WOAH NP	300	Depopulation completed 10/19 (CO_2)	$0	11/22/2022	Owner is not pursuing indemnity.
10/15/2022	10/18/2022	North Dakota	Traill 01	Backyard	WOAH NP	94	Depopulation completed 10/16 (CO_2/gunshot)	$2,680	10/31/2022	
10/14/2022	10/18/2022	Utah	Sanpete 16	Commercial Turkey Meat Bird	Turkey	100,755	Depopulation completed 10/17 (Foam)	$2,289,935	12/26/2022	
10/13/2022	10/14/2022	Arkansas	Pope 01	Pet Bird	WOAH NP	468	Depopulation completed 10/14 (CO_2)	$10,050	10/28/2022	
10/14/2022	10/14/2022	Oklahoma	Tulsa 01	Backyard	WOAH NP	31	Depopulation completed 10/17 (CO_2)	$630	11/1/2022	
10/14/2022	10/17/2022	Pennsylvania	Adams 02	Commercial Turkey Meat Bird	Turkey	15,064	Depopulation completed 10/14 (Foam)	$870,230	11/8/2022	

10/13/2022	10/14/2022	Colorado	Larimer 01	Backyard	WOAH NP	23	Depopulation completed 10/13 (CO2)	$1,100	11/4/2022	
10/13/2022	10/18/2022	Florida	Osceola 02	Backyard	WOAH NP	16	Depopulation completed 10/14 (CO2)	$415	11/8/2022	
10/12/2022	10/13/2022	Utah	Sanpete 15	Commercial Turkey Meat Bird	Turkey	99,960	Depopulation completed 10/16 (Foam)	$1,691,790	11/27/2022	
10/12/2022	10/14/2022	Colorado	La Plata 02	Backyard	WOAH NP	35	No depopulation occurred. All birds died.	$0	11/17/2022	Owner is not pursuing indemnity.
10/13/2022	10/14/2022	Florida	Broward 02	Backyard	Poultry	302	Depopulation completed 10/17 (CO2)	$7,705	11/8/2022	
10/12/2022	10/13/2022	Wisconsin	St. Croix 01	Backyard	WOAH NP	24	Depopulation completed 10/14 (CO2)	$180	11/7/2022	
10/12/2022	10/14/2022	Alaska	Matanuska-Susitna 04	Backyard	Poultry	1,015	Depopulation completed 10/13 (CO2)	$21,545	10/27/2022	
10/12/2022	10/13/2022	New Jersey	Warren 01	Backyard	WOAH NP	6	Depopulation completed 10/14 (CO2)	$125	11/3/2022	
10/11/2022	10/12/2022	Utah	Sanpete 14	Commercial Turkey Meat Bird	Turkey	52,808	Depopulation completed 10/14 (Foam)	$834,735	12/25/2022	
10/7/2022	10/11/2022	Michigan	Lapeer 02	Backyard	Poultry	44	Depopulation completed 10/13 (CO2)	$3,010	10/28/2022	
10/11/2022	10/12/2022	Colorado	Weld 05	Backyard	WOAH NP	40	Depopulation completed 10/13 (CO2)	$935	11/3/2022	
10/11/2022	10/12/2022	Wyoming	Fremont 02	Backyard	WOAH NP	26	No depopulation occurred. All birds died.	$0	11/3/2022	Owner is not pursuing indemnity.
10/10/2022	10/13/2022	California	Stanislaus 02	Commercial Turkey Meat Bird	Turkey	54,931	Depopulation completed 10/12 (Foam)	$732,230	10/31/2022	
10/10/2022	10/11/2022	Pennsylvania	Adams 01	Backyard	Poultry	2,844	Depopulation completed 10/11 (Foam)	$53,100	10/31/2022	
10/10/2022	10/11/2022	Kentucky	Logan 01	Backyard	WOAH NP	187	Depopulation completed 10/12 (CO2/long netting)	$4,455	11/2/2022	
10/10/2022	10/12/2022	Michigan	Genesee 01	Backyard	WOAH NP	23	Depopulation completed 10/11 (Injectable)	$115	10/26/2022	
10/6/2022	10/11/2022	South Dakota	Gregory 01	Commercial Upland Gamebird Producer	Pheasant	200	Depopulation completed 10/7 (Cervical dislocation)	$1,130	11/16/2022	

10/8/2022	10/12/2022	South Dakota	Beadle 05	Commercial Turkey Meat Bird	Turkey	71,413	Depopulation completed 10/10 (VSD+ heat/foam)	$1,611,615	11/8/2022	This is a previously affected premises (Beadle 04).
10/7/2022	10/12/2022	South Dakota	Hamlin 02	Backyard	WOAH NP	21	Depopulation completed 10/8 (Cervical dislocation)	$455	11/4/2022	
10/7/2022	10/11/2022	Florida	Martin 01	Backyard	WOAH NP	51	Depopulation completed (CO2)	$415	11/2/2022	
10/6/2022	10/7/2022	Arkansas	Madison 01	Commercial Broiler Breeder Pullets	Chicken	56,000	Depopulation completed 10/7 (Foam)	$890,910	11/4/2022	
10/7/2022	10/11/2022	Florida	Broward 01	Backyard	WOAH NP	35	Depopulation completed 10/7 (CO2)	$0	10/28/2022	Owner is not pursuing indemnity.
10/5/2022	10/6/2022	Kentucky	Fayette 01	Backyard	Poultry	148	Depopulation completed 10/8 (CO2)	$5,410	10/22/2022	
10/6/2022	10/7/2022	Utah	Sanpete 13	Commercial Turkey Meat Bird	Turkey	38,906	Depopulation completed 10/7 (Foam)	$367,660	11/5/2022	
10/6/2022	10/7/2022	Utah	Sanpete 12	Commercial Turkey Meat Bird	Turkey	26,658	Depopulation completed 10/7 (Foam)	$887,615	12/27/2022	
10/5/2022	10/7/2022	Colorado	Boulder 01	Backyard	Poultry	600	Depopulation completed 10/11 (CO2)	$4,675	10/28/2022	
10/5/2022	10/6/2022	Virginia	Hampton 01	Petting Zoo/Exhibition Farm	WOAH NP	39	No depopulation planned.	$0	11/3/2022	Owner is not pursuing indemnity.
10/5/2022	10/7/2022	Minnesota	Pope 01	Backyard	WOAH NP	28	Depopulation completed 10/5 (CO2)	$50	10/21/2022	
10/5/2022	10/7/2022	Michigan	Lapeer 01	Backyard	Poultry	100	Depopulation completed 10/4 (Barbiturate/cervical dislocation)	$3,010	10/28/2022	
10/4/2022	10/6/2022	South Dakota	Brule 02	Commercial Upland Gamebird Producer	Pheasant	500	Depopulation completed 10/7 (Cervical dislocation)	$2,925	10/27/2022	
10/4/2022	10/5/2022	Pennsylvania	Monroe 01	Backyard	WOAH NP	37	Depopulation completed 10/3 (CO2)	$1,260	10/26/2022	
10/4/2022	10/7/2022	Kansas	Neosho 01	Backyard	Poultry	2,000	Depopulation completed 10/6 (CO2)	$32,060	10/21/2022	

Sample sent directly to NVSL	10/4/2022	New Mexico	Bernalillo 01	Backyard	WOAH NP	37	Depopulation completed 10/5 (VSD+ heat/foam/CO2)	$0	11/9/2022	Owner is not pursuing indemnity.
10/4/2022	10/7/2022	Kansas	Johnson 01	Backyard	Poultry	6	Depopulation completed 10/5 (Injectable)	$2,850	11/8/2022	
10/3/2022	10/5/2022	Pennsylvania	Berks 11	Backyard	WOAH NP	14	Depopulation completed 10/5 (Injectable)	$555	10/26/2022	
10/4/2022	10/5/2022	Connecticut	New Haven 01	Backyard	WOAH NP	52	Depopulation completed 10/5 (CO2)	$1,310	10/28/2022	
10/4/2022	10/5/2022	Montana	Glacier 02	Backyard	WOAH NP	1,050	Depopulation completed 10/5 (Stunning/exsanguination)	$16,440	11/6/2022	
10/3/2022	10/4/2022	Pennsylvania	Westmoreland 01	Backyard	WOAH NP	158	Depopulation completed 10/5 (CO2)	$2,295	10/24/2022	
10/3/2022	10/4/2022	Nebraska	Box Butte 01	Backyard	WOAH NP	104	Depopulation completed 10/4 (Barbiturate/cervical dislocation)	$1,235	10/20/2022	
10/1/2022	10/4/2022	California	Del Norte 01	Backyard	Poultry	43,000	Depopulation completed 10/7 (Cervical dislocation)	$200,710	10/25/2022	
10/1/2022	10/5/2022	Alaska	Matanuska-Susitna 03	Backyard	Poultry	11	Depopulation completed 10/3 (CO2)	$1,000	10/22/2022	
10/1/2022	10/4/2022	California	Monterey 01	Commercial Duck Breeder	Duck	15,069	Depopulation completed 10/6 (CO2)	$517,280	10/30/2022	
9/30/2022	10/4/2022	Nebraska	York 01	Commercial Upland Gamebird Producer	Pheasant	139,490	Depopulation completed 10/5 (VSD+ heat/foam/CO2)	$1,269,140	11/16/2022	
9/30/2022	10/3/2022	Utah	Cache 03	Pet Bird	WOAH NP	322	No depopulation planned.	$0	11/3/2022	Owner is not pursuing indemnity.
9/29/2022	10/3/2022	California	Stanislaus 01	Commercial Turkey Meat Bird	Turkey	50,976	Depopulation completed 10/2 (Foam)	$679,550	10/21/2022	
9/29/2022	9/29/2022	Colorado	Mesa 01	Backyard	WOAH NP	35	Depopulation completed 9/30 (CO2)	$1,480	10/20/2022	
9/29/2022	10/3/2022	Michigan	Tuscola 01	Backyard	Poultry	140	Depopulation completed 9/30 (CO2)	$755	10/15/2022	
9/29/2022	10/5/2022	Alaska	Matanuska-Susitna 02	Backyard	WOAH NP	40	Depopulation completed 9/30 (CO2)	$700	10/20/2022	

9/29/2022	10/3/2022	Ohio	Portage 01	Backyard	WOAH NP	46	Depopulation completed 10/3 (Cervical dislocation)	$1,210	10/18/2022	
9/29/2022	9/30/2022	North Dakota	Nelson 01	Backyard	WOAH NP	54	Depopulation completed 9/29 (CO2)	$60	10/18/2022	
9/29/2022	9/29/2022	Utah	Sanpete 11	Commercial Turkey Meat Bird	Turkey	19,874	Depopulation completed 10/1 (Foam/cervical dislocation)	$353,160	11/17/2022	
9/29/2022	9/29/2022	Utah	Sanpete 10	Commercial Turkey Meat Bird	Turkey	19,316	Depopulation completed 10/1 (Foam/cervical dislocation)	$475,560	10/30/2022	
9/29/2022	9/30/2022	Oregon	Douglas 01	Backyard	WOAH NP	31	Depopulation completed 9/30 (CO2/firearm)	$500	10/15/2022	
9/28/2022	9/29/2022	Pennsylvania	York 01	Commercial Turkey Meat Bird	Turkey	25,931	Depopulation completed 9/29 (Foam)	$544,875	10/26/2022	
9/27/2022	9/29/2022	Wisconsin	Dunn 02	Commercial Turkey Meat Bird	Turkey	79,500	Depopulation completed 9/28 (Foam)	$1,510,355	10/20/2022	
9/27/2022	9/29/2022	Pennsylvania	Lancaster 09	Backyard	WOAH NP	178	Depopulation completed 9/29 (Cervical dislocation)	$345	10/17/2022	
9/27/2022	9/28/2022	Utah	Sanpete 09	Commercial Turkey Meat Bird	Turkey	24,376	Depopulation completed 9/29 (Foam/cervical dislocation)	$423,060	10/21/2022	
9/26/2022	9/27/2022	Utah	Sanpete 08	Commercial Turkey Meat Bird	Turkey	36,700	Depopulation completed 9/29 (VSD+ heat/cervical dislocation)	$1,185,485	10/29/2022	
9/26/2022	9/27/2022	Utah	Sanpete 07	Commercial Turkey Meat Bird	Turkey	127,187	Depopulation completed 9/30 (VSD+ heat)	$3,608,220	11/11/2022	
9/27/2022	10/3/2022	California	Calaveras 01	Backyard	WOAH NP	23	Depopulation completed 9/27 (CO2)	$420	10/12/2022	
9/26/2022	9/28/2022	Wisconsin	Racine 02	Commercial Duck Meat Bird	Duck	10,000	Depopulation completed 9/29 (Foam/cervical dislocation)	$34,320	11/10/2022	
9/26/2022	9/28/2022	Oregon	Tillamook 02	Backyard	WOAH NP	36	Depopulation completed 9/28 (CO2/gunshot)	$1,060	10/14/2022	
9/26/2022	9/28/2022	Oregon	Tillamook 01	Backyard	WOAH NP	10	Depopulation completed 9/28 (CO2/gunshot)	$40	10/14/2022	
9/25/2022	9/27/2022	North Dakota	Ransom 01	Commercial Turkey Meat Bird	Turkey	69,090	Depopulation completed 9/27 (VSD+ heat/foam)	$1,529,475	10/27/2022	

9/25/2022	9/27/2022	Minnesota	Goodhue 01	Backyard	WOAH NP	78	Depopulation completed 9/26 (CO2)	$1,790	10/12/2022	
9/22/2022	9/24/2022	Texas	Dallas 01	Backyard	WOAH NP	63	Depopulation completed 10/2 (CO2)	$9,060	10/20/2022	
Sample sent directly to NVSL	9/23/2022	New Hampshire	Merrimack 01	Backyard	Poultry	245	Depopulation completed 9/26 (Cervical dislocation)	$1,930	10/8/2022	
9/23/2022	9/24/2022	Delaware	Kent 03	Backyard	WOAH NP	26	Depopulation completed 9/23 (CO2/captive bolt/cervical dislocation)	$520	10/11/2022	
9/22/2022	9/24/2022	South Dakota	McPherson 05	Commercial Turkey Meat Bird	Turkey	56,919	Depopulation completed 9/25 (VSD+ heat/foam)	$1,279,305	10/12/2022	
9/23/2022	9/24/2022	Delaware	Kent 02	Backyard	WOAH NP	11	Depopulation completed 9/23 (CO2/captive bolt/cervical dislocation)	$160	10/11/2022	
Sample sent directly to NVSL	9/21/2022	Idaho	Gooding 03	Commercial Upland Gamebird Producer	Pheasant	9,000	Depopulation completed 9/25 (CO2)	$54,890	10/14/2022	
9/20/2022	9/21/2022	Utah	Sanpete 06	Commercial Turkey Meat Bird	Turkey	11,063	Depopulation completed 9/22 (VSD+ heat)	$642,980	10/17/2022	
9/20/2022	9/21/2022	Colorado	Weld 04	Commercial Table Egg Layer	Chicken	1,150,000	Depopulation completed 10/10 (VSD+ heat/CO2)	$4,359,243	11/4/2022	
9/21/2022	9/22/2022	North Dakota	Ward 01	Backyard	Poultry	56	Depopulation completed 9/21(Owner depopulated)	$555	10/5/2022	
9/20/2022	9/22/2022	South Dakota	Kingsbury 03	Backyard	WOAH NP	253	Depopulation completed 9/22 (Cervical dislocation)	$3,380	10/12/2022	
9/21/2022	9/22/2022	Maryland	Anne Arundel 01	Backyard	WOAH NP	4	Depopulation completed 9/24 (CO2)	$85	10/9/2022	
	No Testing - Dangerous Contact	Pennsylvania	Washington 02	Backyard	Poultry	56	Depopulation completed 9/21 (CO2)	$3,210		Dangerous contact for Washington 01.
	No Testing - Dangerous Contact	Pennsylvania	Washington 03	Backyard	Poultry	25	Depopulation completed 9/21 (CO2)	$320		Dangerous contact for Washington 01.
9/20/2022	9/22/2022	Minnesota	Todd 07	Backyard	WOAH NP	47	Depopulation completed 9/21 (CO2/cervical dislocation)	$1,120	10/6/2022	

9/20/2022	9/21/2022	Minnesota	Roseau 01	Commercial Turkey Meat Bird	Turkey	115,982	Depopulation completed 9/21 (Foam)	$1,854,645	10/29/2022	
9/19/2022	9/21/2022	Minnesota	Otter Tail 06	Commercial Turkey Meat Bird	Turkey	5,949	Depopulation completed 9/20 (Foam)	$180,600	10/12/2022	
9/19/2022	9/20/2022	Utah	Sanpete 05	Commercial Turkey Meat Bird	Turkey	73,152	Depopulation completed 9/19 (VSD+ heat)	$2,733,265	10/26/2022	
9/19/2022	9/20/2022	Pennsylvania	Washington 01	Backyard	Poultry	1,828	Depopulation completed 9/21 (CO2)	$2,930	10/7/2022	
9/17/2022	9/20/2022	South Dakota	Clark 05	Commercial Turkey Meat Bird	Turkey	23,866	Depopulation completed 9/19 (VSD+ heat/foam)	$385,769	10/18/2022	This is a previously affected premises (Clark 02).
9/17/2022	9/20/2022	South Dakota	Clark 04	Commercial Turkey Meat Bird	Turkey	41,711	Depopulation completed 9/18 (VSD+ heat/foam)	$892,900	10/11/2022	This is a previously affected premises (Clark 01).
9/17/2022	9/20/2022	Minnesota	Stearns 11	Commercial Turkey Meat Bird	Turkey	64,616	Depopulation completed 9/18 (Foam)	$1,944,940	10/13/2022	This is a previously affected premises (Stearns 06).
9/16/2022	9/20/2022	California	Fresno 06	Backyard	WOAH NP	95	Depopulation completed 9/23 (CO2/cervical dislocation)	$1,755	10/8/2022	
9/16/2022	9/20/2022	Minnesota	Brown 02	Commercial Turkey Meat Bird	Turkey	42,974	Depopulation completed 9/17 (Foam)	$827,810	10/8/2022	
9/16/2022	9/19/2022	Nebraska	Dawes 01	Backyard	WOAH NP	44	Depopulation completed 9/17 (Cervical dislocation)	$680	10/6/2022	
9/16/2022	9/19/2022	Michigan	Macomb 03	Backyard	WOAH NP	35	Depopulation completed 9/17 (CO2)	$820	10/2/2022	
9/14/2022	9/15/2022	Ohio	Summit 01	Backyard	WOAH NP	6	Depopulation completed 9/15 (Cervical dislocation)	$100	10/1/2022	
9/14/2022	9/16/2022	Washington	Pierce 05	Backyard	WOAH NP	26	Depopulation completed 9/15 (CO2)	$905	10/19/2022	
9/14/2022	9/15/2022	Massachusetts	Bristol 01	Backyard	WOAH NP	46	Depopulation completed 9/16 (CO2)	$1,140	9/30/2022	
9/14/2022	9/15/2022	Tennessee	Obion 01	Backyard	Poultry	132	Depopulation completed 9/15 (Cervical dislocation)	$0	9/30/2022	Producer is not pursuing indemnity.
9/14/2022	9/15/2022	California	Fresno 05	Commercial Broiler Breeder	Chicken	15,919	Depopulation completed 9/15 (Foam/cervical dislocation/long netting)	$82,940	10/15/2022	

9/13/2022	9/14/2022	Minnesota	Isanti 01	Backyard	WOAH NP	4	Depopulation completed 9/14 (CO2)	$95	9/29/2022	
9/13/2022	9/15/2022	Montana	Teton 01	Backyard	Poultry	2,800	Depopulation completed 9/14 (Cervical dislocation/stunning/exsanguination)	$37,325	10/14/2022	
9/12/2022	9/14/2022	Minnesota	Otter Tail 05	Commercial Turkey Breeder Hens	Turkey	7,850	Depopulation completed 9/13 (Foam)	$522,930	10/8/2022	
9/12/2022	9/14/2022	Ohio	Williams 01	Backyard	WOAH NP	63	Depopulation completed 9/14 (CO2/gunshot)	$2,015	9/30/2022	
9/12/2022	9/14/2022	Ohio	Allen 01	Backyard	WOAH NP	2	No depopulation occurred. All birds died.	$0	9/28/2022	Owner is not pursuing indemnity.
Sample sent directly to NVSL	9/12/2022	Idaho	Twin Falls 01	Backyard	WOAH NP	40	Depopulation completed 9/16 (CO2)	$1,020	10/18/2022	
9/10/2022	9/14/2022	Minnesota	Meeker 08	Commercial Turkey Meat Bird	Turkey	61,387	Depopulation completed 9/11 (Foam)	$1,847,750	10/6/2022	
9/12/2022	9/13/2022	Michigan	Ingham 01	Backyard	Poultry	20	Depopulation completed 9/13 (CO2)	$655	9/28/2022	
9/11/2022	9/13/2022	Minnesota	Brown 01	Commercial Turkey Meat Bird	Turkey	43,732	Depopulation completed 9/12 (Foam)	$855,050	9/30/2022	
9/12/2022	9/13/2022	Utah	Sanpete 04	Commercial Turkey Meat Bird	Turkey	5,777	Depopulation completed 9/13 (VSD+ heat)	$173,890	10/11/2022	
9/9/2022	9/13/2022	Minnesota	Freeborn 01	Backyard	WOAH NP	316	Depopulation completed 9/10 (Cervical dislocation)	$17,125	9/26/2022	
9/9/2022	9/13/2022	Minnesota	Stearns 10	Commercial Turkey Meat Bird	Turkey	69,361	Depopulation completed 9/10 (Foam)	$1,350,450	9/29/2022	
9/9/2022	9/9/2022	California	El Dorado 01	Backyard	WOAH NP	154	Depopulation completed 9/15 (CO2/long netting)	$5,290	10/1/2022	
9/7/2022	9/9/2022	California	Fresno 04	Commercial Broiler Breeder	Chicken	23,889	Depopulation completed 9/11 (Foam)	$145,110	10/14/2022	
9/7/2022	9/9/2022	Minnesota	Becker 03	Commercial Turkey Breeder Hens	Turkey	17,110	Depopulation completed 9/10 (VSD+ heat)	$1,491,940	9/30/2022	
9/6/2022	9/7/2022	Minnesota	Morrison 13	Commercial Turkey Meat Bird	Turkey	50,165	Depopulation completed 9/7 (VSD+ heat/foam)	$1,050,741	9/28/2022	

	No Testing - Dangerous Contact	Ohio	Ashland 02	Backyard	WOAH NP	7	Depopulation completed 9/12 (CO2)	$145		
9/2/2022	9/3/2022	Ohio	Ashland 01	Backyard	WOAH NP	635	Depopulation completed 9/12 (CO2/gunshot)	$13,865	9/27/2022	
9/3/2022	9/8/2022	Minnesota	Meeker 07	Commercial Turkey Meat Bird	Turkey	34,203	Depopulation completed 9/4 (Foam)	$981,412	9/30/2022	
9/2/2022	9/3/2022	Ohio	Defiance 01	Commercial Table Egg Layer	Chicken	3,748,544	Depopulation completed 9/13 (VSD+ heat/CO2/cervical dislocation)	$13,635,000	11/5/2022	
8/31/2022	9/2/2022	Wisconsin	Washington 01	Backyard	Poultry	115	Depopulation 9/2 (CO2)	$6,700	9/19/2022	
8/31/2022	9/1/2022	North Dakota	Cass 02	Backyard	WOAH NP	6	Depopulation completed 9/1 (Cervical dislocation)	$25	9/28/2022	
8/30/2022	9/1/2022	California	Sacramento 03	Backyard	WOAH NP	16	Depopulation completed 9/6 (Cervical Dislocation)	$0	9/22/2022	Owner is not pursuing indemnity.
8/30/2022	9/1/2022	California	Fresno 03	Commercial Broiler Breeder	Chicken	22,940	Depopulation completed 9/2 (Foam)	$120,485	10/14/2022	
8/31/2022	9/1/2022	California	Tuolumne 04	Commercial Turkey Meat Bird	Turkey	30,825	Depopulation completed 9/1 (Foam)	$553,055	10/12/2022	
8/30/2022	9/1/2022	Minnesota	Hennepin 01	Backyard	WOAH NP	13	Depopulation completed 8/31 (Cervical dislocation)	$280	9/15/2022	
8/30/2022	9/1/2022	Indiana	Elkhart 04	Backyard	Poultry	261	Depopulation completed 8/31 (CO2/Injectable)	$7,696	9/15/2022	
8/29/2022	8/30/2022	Minnesota	Meeker 06	Commercial Turkey Meat Bird	Turkey	51,958	Depopulation completed 8/30 (Foam)	$1,664,530	9/24/2022	This is a previously affected premises (Meeker 04).
8/27/2022	8/29/2022	Virginia	Caroline 01	Backyard	WOAH NP	114	Depopulation completed 8/28 (CO2)	$4,525	10/4/2022	
8/28/2022	8/30/2022	Minnesota	Meeker 05	Commercial Turkey Meat Bird	Turkey	126,407	Depopulation completed 8/29 (Foam)	$2,989,040	9/24/2022	Epi-link to Meeker 01.
8/26/2022	8/30/2022	California	Tuolumne 03	Commercial Turkey Meat Bird	Turkey	91,000	Depopulation completed 8/30 (Foam)	$1,900,340	10/12/2022	
8/26/2022	8/30/2022	California	Tuolumne 02	Commercial Turkey Meat Bird	Turkey	7,383	Depopulation completed 8/28 (Cervical dislocation)	$162,955	9/22/2022	

8/26/2022	8/30/2022	California	Sacramento 02	Commercial Turkey Meat Bird	Turkey	97,000	Depopulation completed 8/29 (CO2)	$1,724,990	9/22/2022	
8/26/2022	8/26/2022	California	Tuolumne 01	Commercial Turkey Meat Bird	Turkey	161,740	Depopulation completed 8/29 (Foam)	$5,654,335	10/12/2022	
8/25/2022	8/26/2022	California	Fresno 02	Commercial Broiler Breeder	Chicken	34,835	Depopulation completed 8/27 (Foam)	$181,490	9/24/2022	
8/24/2022	8/26/2022	Washington	Cowlitz 01	Backyard	WOAH NP	16	Depopulation completed 8/25 (CO2)	$480	9/14/2022	
8/23/2022	8/25/2022	Washington	Pierce 04	Backyard	WOAH NP	72	Depopulation completed 8/25 (CO2/long netting/cervical dislocation)	$3,325	9/15/2022	
8/23/2022	8/24/2022	Utah	Weber 01	Backyard	WOAH NP	16	Depopulation completed 8/24 (Injectable)	$0	9/9/2022	
8/20/2022	8/22/2022	California	Fresno 01	Commercial Broiler Breeder	Chicken	33,908	Depopulation completed 8/26 (VSD+ heat/foam)	$176,660	9/24/2022	
8/19/2022	8/23/2022	California	Contra Costa 01	Backyard	WOAH NP	62	Depopulation completed 8/22 (CO2)	$2,210	9/8/2022	
8/19/2022	8/22/2022	Georgia	Henry 01	Animal Rescue / Rehabilitation	WOAH NP	119	Depopulation completed 8/26 (CO2)	$6,430	9/10/2022	
8/17/2022	8/19/2022	Washington	Kitsap 02	Backyard	WOAH NP	12	Depopulation completed 8/19 (CO2)	$385	9/9/2022	
8/13/2022	8/16/2022	California	Butte 01	Backyard	WOAH NP	1,080	Depopulation completed 8/19 (CO2)	$10,095	9/8/2022	
8/11/2022	8/16/2022	Florida	Osceola 01	Sentinel Flock	WOAH NP	10	Depopulation completed 8/11 (Injectable)	$0	8/26/2022	Producer is not pursuing indemnity.
8/12/2022	8/18/2022	Washington	Walla Walla 01	Backyard	WOAH NP	2	Depopulation completed 8/16 (CO2)	$40	9/7/2022	
8/8/2022	8/10/2022	California	Sacramento 01	Backyard	WOAH NP	44	Depopulation completed 8/9 (Cervical dislocation)	$0	9/4/2022	Producer is not pursuing indemnity.
8/9/2022	8/11/2022	Pennsylvania	Northampton 01	Backyard	Poultry	88	Depopulation completed 8/11 (CO2)	$1,940	9/2/2022	
8/1/2022	8/2/2022	Alaska	Bethel 01	Backyard	WOAH NP	15	No depopulation occurred. All birds died.	$0	8/29/2022	Owner is not pursuing indemnity.
7/26/2022	7/28/2022	Oregon	Coos 01	Backyard	WOAH NP	23	Depopulation completed 7/27 (Gunshot)	$170	9/21/2022	

7/24/2022	7/26/2022	Utah	Sanpete 03	Commercial Turkey Meat Bird	Turkey	12,689	Depopulation completed 7/26 (VSD+ heat/CO2/cervical dislocation)	$312,400	8/24/2022	
7/22/2022	7/26/2022	Washington	Jefferson 02	Backyard	WOAH NP	78	Depopulation completed 7/23 (CO2)	$1,095	8/26/2022	
7/22/2022	7/26/2022	Montana	Flathead 01	Backyard	WOAH NP	7	Depopulation completed 7/23 (Cervical dislocation)	$150	8/7/2022	
7/21/2022	7/22/2022	Oregon	Deschutes 05	Backyard	WOAH NP	121	Depopulation completed 7/26 (CO2)	$5,400	10/3/2022	
7/21/2022	7/22/2022	Oregon	Deschutes 06	Backyard	WOAH NP	89	Depopulation completed 8/8 (CO2)	$3,025	8/15/2022	
7/20/2022	7/21/2022	Florida	Seminole 01	Backyard	WOAH NP	163	Depopulation completed 7/22 (CO2)	$1,700	8/8/2022	
7/19/2022	7/21/2022	Oregon	Deschutes 04	Backyard	Poultry	55	Depopulation completed 7/20 (CO2)	$1,485	8/15/2022	
7/18/2022	7/19/2022	Utah	Sanpete 02	Commercial Turkey Meat Bird	Turkey	23,327	Depopulation completed 7/19 (VSD+ heat/CO2)	$919,895	9/6/2022	
7/16/2022	7/19/2022	Washington	Snohomish 06	Backyard	Poultry	340	Depopulation completed 7/18 (CO2)	$7,020	8/11/2022	
7/14/2022	7/19/2022	Oregon	Deschutes 03	Backyard	Poultry	101	Depopulation completed 7/15 (CO2)	$2,455	8/15/2022	
7/13/2022	7/14/2022	Utah	Sanpete 01	Commercial Turkey Meat Bird	Turkey	15,524	Depopulation completed 7/13 (VSD+ heat)	$458,845	8/24/2022	
7/12/2022	7/15/2022	Oregon	Deschutes 02	Backyard	Poultry	63	Depopulation completed 7/14 (CO2)	$1,545	8/15/2022	
7/8/2022	7/12/2022	Oregon	Deschutes 01	Backyard	Poultry	99	Depopulation completed 7/11 (CO2)	$2,675	8/15/2022	
Sample sent directly to NVSL	7/7/2022	Nevada	Carson City 01	Backyard	WOAH NP	35	Depopulation completed 7/8 (CO2/cervical dislocation)	$0	7/29/2022	
7/1/2022	7/6/2022	Oregon	Linn 02	Backyard	WOAH NP	38	Depopulation completed 7/2 (CO2)	$785	8/8/2022	
7/1/2022	7/6/2022	Washington	Jefferson 01	Backyard	WOAH NP	23	Depopulation completed 7/2 (CO2)	$650	8/26/2022	
6/30/2022	7/1/2022	Utah	Salt Lake 03	Backyard	WOAH NP	24	Depopulation completed 7/1 (CO2)	$0	7/20/2022	Owner is not pursuing indemnity.

6/29/2022	7/1/2022	Washington	King 07	Backyard	Poultry	215	Depopulation completed 7/2 (CO2)	$11,930	8/1/2022	
6/29/2022	7/1/2022	Washington	Kitsap 01	Backyard	WOAH NP	6	Depopulation completed 6/30 (CO2)	$235	8/4/2022	
6/28/2022	6/29/2022	Maine	Cumberland 02	Backyard	WOAH NP	15	Depopulation completed 6/29 (Injectable)	$460	7/14/2022	
6/27/2022	6/29/2022	Washington	Yakima 04	Backyard	WOAH NP	6	Depopulation completed 6/28 (CO2)	$240	7/28/2022	
6/22/2022	6/24/2022	Washington	Pierce 03	Backyard	WOAH NP	46	Depopulation completed 6/23 (CO2/long range shot)	$2,490	8/10/2022	
6/18/2022	6/22/2022	Washington	Snohomish 05	Backyard	WOAH NP	201	Depopulation completed 6/20 (CO2/firearm)	$8,540	8/1/2022	
6/18/2022	6/22/2022	Washington	King 06	Backyard	WOAH NP	3	Depopulation completed 6/18 (Cervical dislocation)	$0	8/4/2022	Owner is not pursuing indemnity.
6/17/2022	6/22/2022	Washington	Yakima 03	Backyard	WOAH NP	92	Depopulation completed 6/18 (CO2)	$1,430	7/28/2022	
6/17/2022	6/22/2022	Washington	King 05	Backyard	WOAH NP	9	Depopulation completed 6/18 (CO2)	$185	8/4/2022	
6/10/2022	6/13/2022	Utah	Salt Lake 02	Petting Zoo/Exhibition Farm	WOAH NP	68	Depopulation completed 6/11 (CO2)	$1,500	7/6/2022	
6/10/2022	6/14/2022	Washington	Yakima 02	Backyard	WOAH NP	32	Depopulation completed 6/11 (CO2)	$305	7/1/2022	
6/8/2022	6/9/2022	Colorado	Weld 03	Commercial Table Egg Pullets	Chicken	205,019	Depopulation completed 6/10 (CO2)	$637,610	7/18/2022	
Sample sent directly to NVSL	6/8/2022	Oregon	Polk 01	Backyard	WOAH NP	87	Depopulation completed 6/9 (CO2)	$1,090	7/5/2022	
6/7/2022	6/9/2022	Washington	Yakima 01	Backyard	WOAH NP	70	Depopulation completed 6/10 (CO2)	$1,515	7/1/2022	
6/7/2022	6/9/2022	Washington	Snohomish 04	Backyard	WOAH NP	9	Depopulation completed 6/8 (CO2)	$335	7/13/2022	
6/7/2022	6/9/2022	Washington	Snohomish 03	Backyard	WOAH NP	28	Depopulation completed 6/8 (CO2)	$1,520	7/13/2022	
6/6/2022	6/7/2022	Colorado	Weld 02	Commercial Table Egg Layer	Chicken	1,936,776	Depopulation completed 6/24 (VSD+ heat/CO2)	$6,173,690	7/18/2022	

6/3/2022	6/6/2022	North Dakota	McHenry 01	Backyard	Poultry	27	Depopulation completed 6/6 (Cervical dislocation)	$360	6/17/2022	
6/5/2022	6/7/2022	Washington	King 04	Backyard	Poultry	47	Depopulation completed 6/6 (CO2)	$1,380	7/13/2022	
6/3/2022	6/8/2022	Indiana	Allen 03	Backyard	WOAH NP	41	Depopulation completed 6/3 (Injectable)	$1,390	6/18/2022	
6/2/2022	6/7/2022	Indiana	Allen 02	Backyard	Poultry	114	Depopulation completed 6/3 (Injectable)	$2,340	6/18/2022	
6/1/2022	6/2/2022	Pennsylvania	Berks 10	Commercial Duck Meat Bird	Duck	30,100	Depopulation completed 6/2 (VSD+ heat)	$26,422	7/2/2022	
5/31/2022	6/1/2022	Georgia	Toombs 01	Backyard	WOAH NP	488	Depopulation completed 6/2 (CO2)	$13,335	6/17/2022	
5/31/2022	6/2/2022	Washington	Snohomish 02	Backyard	WOAH NP	28	Depopulation completed 6/1 (long netting/CO2)	$1,265	6/16/2022	
Sample sent directly to NVSL	5/27/2022	Washington	Snohomish 01	Backyard	WOAH NP	38	Depopulation completed 5/28 (CO2)	$690	6/16/2022	
5/27/2022	5/31/2022	Minnesota	Becker 02	Backyard	WOAH NP	35	Depopulation completed 5/27 (CO2/mechanically assisted cervical dislocation)	$870	6/11/2022	
Sample sent directly to NVSL	5/25/2022	Washington	King 03	Backyard	WOAH NP	20	Depopulation completed 5/27 (CO2)	$1,005	6/14/2022	
Sample sent directly to NVSL	5/24/2022	Washington	King 01	Backyard	WOAH NP	7	Depopulation completed 5/27 (CO2)	$105	6/18/2022	
Sample sent directly to NVSL	5/24/2022	Idaho	Ada 11	Backyard	WOAH NP	28	Depopulation completed 5/27 (CO2)	$730	6/11/2022	
Sample sent directly to NVSL	5/24/2022	Washington	King 02	Backyard	WOAH NP	183	Depopulation completed 5/27 (CO2)	$4,335	6/18/2022	
5/20/2022	5/23/2022	Wisconsin	Bayfield 01	Backyard	WOAH NP	38	Depopulation completed 5/23 (CO2)	$870	6/8/2022	
5/20/2022	5/23/2022	Pennsylvania	Berks 09	Commercial Duck Breeder	Duck	4,651	Depopulation completed 5/22 (Foam)	$29,655	7/6/2022	

Sample sent directly to NVSL	5/20/2022	Idaho	Ada 10	Backyard	WOAH NP	7	Depopulation completed 5/26 (CO2)	$185	6/10/2022	
5/19/2022	5/21/2022	Minnesota	Dakota 01	Commercial Turkey Meat Bird	Turkey	54,443	Depopulation completed 5/20 (Foam/Mechanically assisted cervical dislocation)	$874,545	6/8/2022	
5/19/2022	5/20/2022	South Dakota	Codington 01	Backyard	Poultry	80	Depopulation completed 5/21 (Cervical dislocation)	$285	6/7/2022	
Sample sent directly to NVSL	5/19/2022	Idaho	Ada 09	Backyard	WOAH NP	25	Depopulation completed 5/23 (CO2)	$440	6/7/2022	
Sample sent directly to NVSL	5/19/2022	Idaho	Canyon 09	Backyard	WOAH NP	13	Depopulation completed 5/23 (CO2)	$20	6/9/2022	
Sample sent directly to NVSL	5/19/2022	Idaho	Canyon 10	Backyard	WOAH NP	14	Depopulation completed 5/24 (CO2)	$350	6/9/2022	
Sample sent directly to NVSL	5/19/2022	Idaho	Ada 08	Backyard	Poultry	65	All birds died 5/23. No depopulation occurred.	$0	6/9/2022	Owner is not pursuing indemnity.
Sample sent directly to NVSL	5/18/2022	Idaho	Canyon 08	Backyard	WOAH NP	12	All birds died 5/17. No depopulation occurred.	$100	6/2/2022	Producer is not pursuing indemnity.
Sample sent directly to NVSL	5/18/2022	Idaho	Ada 07	Backyard	WOAH NP	6	All birds died 5/18. No depopulation occurred.	$120	6/2/2022	
Sample sent directly to NVSL	5/18/2022	Idaho	Canyon 07	Backyard	Poultry	6	Depopulation completed 5/20 (CO2)	$0	6/4/2022	Owner is not pursuing indemnity.
5/18/2022	5/20/2022	Minnesota	Polk 01	Backyard	WOAH NP	46	Depopulation completed 5/19 (CO2)	$1,250	6/4/2022	
5/18/2022	5/20/2022	Wyoming	Campbell 01	Backyard	WOAH NP	34	Depopulation completed 5/21	$520	6/23/2022	
Sample sent directly to NVSL	5/18/2022	Idaho	Canyon 06	Backyard	Poultry	160	Depopulation completed 5/25 (CO2)	$7,055	6/16/2022	

Sample sent directly to NVSL	5/17/2022	Washington	Thurston 01	Backyard	WOAH NP	414	Depopulation completed 5/23 (CO2)	$16,100	6/10/2022	
5/17/2022	5/19/2022	Colorado	Jefferson 01	Animal Rescue / Rehabilitation	WOAH NP	10	Depopulation completed 5/18 (Injectable)	$0	6/2/2022	Producer is not pursuing indemnity.
5/16/2022	5/17/2022	Oregon	Lane 01	Backyard	Poultry	447	Depopulation completed 5/18 (CO2)	$15,355	6/6/2022	
5/14/2022	5/17/2022	Illinois	Boone 01	Backyard	WOAH NP	20	Depopulation completed 5/16 (Cervical dislocation)	$155	6/1/2022	
5/16/2022	5/17/2022	Utah	Salt Lake 01	Backyard	WOAH NP	27	Depopulation completed 5/17 (CO2)	$0	6/16/2022	Producer is not pursuing indemnity.
Sample sent directly to NVSL	5/17/2022	Idaho	Canyon 05	Backyard	Poultry	27	Depopulation completed 5/19 (CO2)	$945	6/3/2022	
Sample sent directly to NVSL	5/17/2022	Idaho	Canyon 04	Backyard	WOAH NP	50	Depopulation completed 5/19 (CO2)	$0	6/3/2022	
Sample sent directly to NVSL	5/17/2022	Idaho	Canyon 03	Backyard	Poultry	30	Depopulation completed 5/24 (CO2)	$580	6/9/2022	
Sample sent directly to NVSL	5/17/2022	Idaho	Ada 06	Backyard	Poultry	9	Depopulation completed 5/20 (CO2)	$45	6/4/2022	
Sample sent directly to NVSL	5/17/2022	Idaho	Ada 05	Backyard	WOAH NP	20	All birds died 5/17. No depopulation occurred.	$450	6/1/2022	
5/17/2022	5/18/2022	Minnesota	Kandiyohi 09	Commercial Turkey Breeder Toms	Turkey	4,705	Depopulation completed 5/18 (Foam)	$764,895	6/11/2022	
5/16/2022	5/18/2022	Pennsylvania	Berks 08	Commercial Duck Breeder	Duck	7,156	Depopulation completed 5/18 (Foam)	$40,155	6/25/2022	
5/16/2022	5/17/2022	North Dakota	Burke 01	Backyard	WOAH NP	45	Depopulation completed 5/17 (CO2)	$420	6/10/2022	
5/16/2022	5/17/2022	New Jersey	Monmouth 01	Backyard	Poultry	62	Depopulation completed 5/18 (CO2)	$2,590	6/16/2022	

5/14/2022	5/18/2022	Minnesota	Clay 01	Backyard	WOAH NP	24	Depopulation completed 5/16 (Mechanically assisted cervical dislocation)	$560	6/4/2022	
5/14/2022	5/17/2022	Pennsylvania	Berks 07	Commercial Table Egg Layer	Chicken	79,048	Depopulation completed 5/17 (VSD+ heat)	$128,675	6/25/2022	
5/13/2022	5/16/2022	Wisconsin	Dunn 01	Backyard	WOAH NP	150	Depopulation completed 5/16 (Producer)	$2,195	6/6/2022	
5/13/2022	5/14/2022	Pennsylvania	Berks 06	Commercial Table Egg Breeder	Chicken	83,729	Depopulation completed 5/15 (VSD+ heat)	$306,740	7/1/2022	
Sample sent directly to NVSL	5/13/2022	Idaho	Canyon 02	Backyard	WOAH NP	9	Depopulation completed 5/16 (CO2)	$0	5/31/2022	
Sample sent directly to NVSL	5/13/2022	Idaho	Ada 04	Backyard	WOAH NP	63	Depopulation completed 5/17 (CO2/injectable)	$3,685	6/1/2022	
5/12/2022	5/13/2022	Wisconsin	Marinette 01	Backyard	WOAH NP	92	Depopulation completed 5/14 (CO2)	$1,672	6/2/2022	
Sample sent directly to NVSL	5/12/2022	Washington	Okanogan 01	Backyard	WOAH NP	24	Depopulation completed 5/13 (CO2)	$835	6/1/2022	
Sample sent directly to NVSL	5/12/2022	Washington	Whatcom 01	Backyard	WOAH NP	77	Depopulation completed 5/13 (CO2)	$2,415	6/3/2022	
5/12/2022	5/19/2022	Indiana	Allen 01	Backyard	WOAH NP	7	Depopulation completed 5/13 (Injectable)	$0	6/4/2022	Producer is not pursuing indemnity.
5/12/2022	5/13/2022	Wyoming	Sheridan 02	Backyard	WOAH NP	24	All birds died 6/13. No depopulation occurred.	$195	6/28/2022	
5/12/2022	5/13/2022	Wyoming	Lincoln 01	Backyard	WOAH NP	44	Depopulation completed 5/12 (Cervical dislocation)	$235	6/1/2022	
5/11/2022	5/13/2022	Wisconsin	Barron 07	Commercial Turkey Meat Bird	Turkey	10,479	Depopulation completed 5/12 (Foam)	$257,995	6/6/2022	
Sample sent directly to NVSL	5/11/2022	Washington	Clallam 02	Backyard	WOAH NP	10	Depopulation completed 5/12 (CO2)	$205	6/4/2022	

Sample sent directly to NVSL	5/11/2022	Washington	Clallam 01	Backyard	WOAH NP	12	Depopulation completed 5/12 (CO2)	$850	6/4/2022	
5/11/2022	5/12/2022	Idaho	Ada 03	Backyard	WOAH NP	12	Depopulation completed 5/13 (CO2)	$670	5/28/2022	
5/10/2022	5/12/2022	Minnesota	Grant 01	Backyard	WOAH NP	309	Depopulation completed 5/11 (CO2)	$3,760	5/26/2022	
5/10/2022	5/12/2022	Minnesota	Chisago 05	Backyard	WOAH NP	37	Depopulation completed 5/11 (CO2)	$1,670	5/26/2022	
Sample sent directly to NVSL	5/10/2022	Washington	Pierce 02	Backyard	WOAH NP	20	Depopulation completed 5/10 (CO2)	$820	6/10/2022	
Sample sent directly to NVSL	5/10/2022	Washington	Pierce 01	Backyard	WOAH NP	24	Depopulation completed 5/14 (CO2/gunshot)	$2,080	6/10/2022	
5/9/2022	5/11/2022	Idaho	Ada 02	Backyard	WOAH NP	4	Depopulation completed 5/11 (CO2)	$0	5/26/2022	Producer is not pursuing indemnity.
5/9/2022	5/10/2022	Idaho	Ada 01	Backyard	Poultry	69	Depopulation completed 5/24 (CO2)	$280	6/8/2022	
5/10/2022	5/11/2022	Utah	Cache 02	Backyard	WOAH NP	16	Depopulation completed 5/11 (CO2)	$1,375	6/24/2022	
5/10/2022	5/11/2022	Minnesota	Chisago 04	Backyard	Poultry	155	Depopulation completed 5/11 (CO2)	$2,605	5/28/2022	
Sample sent directly to NVSL	5/9/2022	Idaho	Canyon 01	Backyard	Poultry	49	Depopulation completed 5/10 (CO2)	$1,475	5/25/2022	
5/9/2022	5/10/2022	Michigan	Muskegon 01	Commercial Turkey Meat Bird	Turkey	35,132	Depopulation completed 5/10 (VSD+ heat)	$648,695	6/7/2022	
5/8/2022	5/10/2022	Pennsylvania	Berks 05	Commercial Duck Meat Bird	Duck	37,320	Depopulation completed 5/10 (VSD+ heat)	$33,675	6/17/2022	
5/8/2022	5/10/2022	Minnesota	Crow Wing 01	Backyard	Poultry	145	Depopulation completed 5/9 (CO2)	$7,190	5/25/2022	
5/8/2022	5/10/2022	Minnesota	Todd 06	Backyard	WOAH NP	52	Depopulation completed 5/9 (CO2)	$1,010	5/26/2022	
5/7/2022	5/10/2022	Pennsylvania	Lancaster 08	Commercial Table Egg Layer	Chicken	72,315	Depopulation completed 5/9 (VSD+ heat)	$221,705	6/18/2022	

5/7/2022	5/10/2022	Pennsylvania	Berks 04	Commercial Duck Breeder	Duck	23,780	Depopulation completed 5/8 (Foam)	$154,430	6/30/2022	
Sample sent directly to NVSL	5/5/2022	Washington	Pacific 01	Backyard	Poultry	38	Depopulation completed 5/6 (CO2)	$425	6/4/2022	
5/6/2022	5/7/2022	Wisconsin	Barron 06	Commercial Turkey Meat Bird	Turkey	20,102	Depopulation completed 5/6 (VSD+ heat)	$605,070	6/6/2022	
5/5/2022	5/7/2022	Washington	Spokane 01	Backyard	WOAH NP	81	Depopulation completed 5/7 (CO2/gunshot)	$1,890	5/26/2022	
5/4/2022	5/5/2022	Wisconsin	Pierce 01	Backyard	WOAH NP	33	Depopulation completed 5/5 (CO2)	$1,340	5/23/2022	
5/4/2022	5/6/2022	Michigan	Oakland 01	Backyard	WOAH NP	46	Depopulation completed 5/6 (CO2)	$750	5/21/2022	
5/4/2022	5/6/2022	Minnesota	Anoka 01	Backyard	WOAH NP	10	Depopulation completed 5/5 (CO2)	$435	5/20/2022	
5/4/2022	5/6/2022	Montana	Fergus 02	Backyard	WOAH NP	19	Depopulation completed 5/4 (Producer)	$270	6/2/2022	
5/3/2022	5/5/2022	Oregon	Linn 01	Backyard	WOAH NP	90	Depopulation completed 5/6 (CO2/Captive bolt)	$3,930	6/1/2022	
5/2/2022	5/4/2022	South Dakota	Hamlin 01	Backyard	WOAH NP	17	All birds died 5/4. No depopulation occurred.	$0	6/15/2022	Owner is not pursuing indemnity.
5/2/2022	5/4/2022	South Dakota	Day 01	Backyard	Poultry	80	Depopulation completed 5/3 (Cervical dislocation)	$0	6/3/2022	Owner is not pursuing indemnity.
5/3/2022	5/4/2022	Minnesota	Carver 02	Backyard	WOAH NP	30	Depopulation completed 5/3 (CO2/Mechanically assisted cervical dislocation)	$440	5/20/2022	
5/3/2022	5/3/2022	Illinois	Kane 01	Backyard	WOAH NP	45	Depopulation completed 5/4 (Mechanically assisted cervical dislocation)	$500	6/1/2022	
5/2/2022	5/4/2022	Iowa	Bremer 02	Backyard	WOAH NP	46	Depopulation completed 5/3 (CO2)	$0	5/19/2022	Owner is not pursuing indemnity.
	No Testing - Dangerous Contact	Pennsylvania	Berks 03	Commercial Poultry Slaughter	Duck	191,430	Depopulation completed 5/8 (Foam)	$512,890		

5/2/2022	5/3/2022	Pennsylvania	Berks 02	Commercial Duck Meat Bird	Duck	74,271	Depopulation completed 5/4 (VSD+ heat/Mechanically assisted cervical dislocation)	$65,050	5/31/2022	
5/2/2022	5/3/2022	Pennsylvania	Berks 01	Commercial Duck Breeder	Duck	29,599	Depopulation completed 5/4 (Foam)	$144,370	5/31/2022	
4/30/2022	5/3/2022	South Dakota	Beadle 04	Commercial Turkey Meat Bird	Turkey	56,248	Depopulation completed 5/2 (VSD+ heat/foam)	$1,194,760	5/25/2022	
5/1/2022	5/3/2022	Wisconsin	Barron 05	Commercial Turkey Meat Bird	Turkey	46,667	Depopulation completed 5/2 (VSD+ heat/foam)	$1,404,675	5/27/2022	
5/1/2022	5/3/2022	Wisconsin	Sauk 01	Backyard	WOAH NP	39	Depopulation completed 5/3 (CO2)	$1,090	5/18/2022	
5/1/2022	5/3/2022	Minnesota	Lyon 01	Commercial Turkey Meat Bird	Turkey	120,303	Depopulation completed 5/3 (Foam)	$1,633,875	6/1/2022	
4/30/2022	5/3/2022	Minnesota	Chisago 03	Backyard	WOAH NP	30	Depopulation completed 5/2 (CO2)	$885	5/17/2022	
4/30/2022	5/3/2022	Wisconsin	Barron 04	Commercial Turkey Meat Bird	Turkey	19,319	Depopulation completed 5/1 (VSD+ heat)	$574,700	5/27/2022	
4/30/2022	5/3/2022	Wisconsin	Polk 03	Backyard	WOAH NP	122	Depopulation completed 5/3 (CO2)	$1,050	5/23/2022	
4/29/2022	4/30/2022	Oklahoma	Sequoyah 01	Commercial Broiler Breeder	Chicken	13,778	Depopulation completed 4/23 (Mechanically assisted cervical dislocation)	$67,175	5/19/2022	
Sample sent directly to NVSL	4/29/2022	Alaska	Matanuska-Susitna 01	Backyard	WOAH NP	27	Depopulation completed 4/23 (Foam)	$315	5/25/2022	
4/29/2022	4/30/2022	Minnesota	Chisago 01	Backyard	WOAH NP	58	Depopulation completed 4/30 (CO2)	$1,175	5/19/2022	
4/29/2022	4/30/2022	Minnesota	Chisago 02	Backyard	WOAH NP	30	Depopulation completed 5/1 (Mechanically assisted cervical dislocation/CO2)	$550	5/17/2022	
4/29/2022	4/29/2022	Colorado	Weld 01	Commercial Table Egg Layer	Chicken	1,366,236	Depopulation completed 5/9 (CO2/VSD+ heat)	$5,281,410	6/8/2022	
4/29/2022	4/30/2022	Minnesota	Morrison 12	Backyard	WOAH NP	54	Depopulation completed 4/30 (CO2)	$2,025	5/16/2022	
4/29/2022	4/30/2022	Michigan	Branch 01	Backyard	WOAH NP	18	Depopulation completed 4/30 (Injectable)	$65	5/15/2022	

4/28/2022	5/3/2022	Wisconsin	Fond du Lac 01	Backyard	WOAH NP	17	Depopulation completed 4/29 (Mechanically assisted cervical dislocation)	$395	5/20/2022	
4/28/2022	4/29/2022	Wisconsin	Oconto 01	Backyard	WOAH NP	222	Depopulation completed 4/29 (C02)	$23,005	5/16/2022	
4/28/2022	4/29/2022	Montana	Fergus 01	Backyard	WOAH NP	106	Depopulation completed 4/30 (Cervical dislocation)	$1,725	6/2/2022	
4/28/2022	4/29/2022	Montana	Gallatin 01	Backyard	WOAH NP	20	Depopulation completed 5/2 (Cervical dislocation)	$230	6/4/2022	
4/28/2022	4/28/2022	Vermont	Caledonia 01	Backyard	WOAH NP	31	Depopulation completed 4/27 (CO2)	$245	5/12/2022	
4/28/2022	4/29/2022	Pennsylvania	Lancaster 07	Commercial Duck Meat Bird	Duck	19,320	Depopulation completed 4/28 (VSD+ heat)	$17,385	5/20/2022	
4/28/2022	4/29/2022	Wisconsin	Barron 03	Backyard	WOAH NP	150	Depopulation completed 4/28 (Producer)	$0	5/27/2022	Producer is not pursuing indemnity.
4/27/2022	4/29/2022	Montana	Pondera 01	Backyard	WOAH NP	2,000	Depopulation completed 4/29 (Cervical dislocation)	$4,425	6/5/2022	
4/27/2022	4/29/2022	Nebraska	Washington 01	Backyard	WOAH NP	52	Depopulation completed 4/28 (Injectable barbiturate/cervical dislocation)	$1,040	5/12/2022	
4/27/2022	4/29/2022	Minnesota	Stearns 09	Commercial Turkey Breeder Hens	Turkey	9,558	Depopulation completed 4/28 (Foam)	$680,150	6/2/2022	
4/27/2022	4/28/2022	Michigan	Wexford 01	Backyard	Poultry	65	Depopulation completed 4/28 (CO2)	$1,865	5/13/2022	
4/27/2022	4/28/2022	Wisconsin	Barron 02	Commercial Turkey Meat Bird	Turkey	110,731	Depopulation completed 4/27 (Foam)	$2,195,560	5/27/2022	
4/26/2022	4/27/2022	Kansas	Republic 01	Backyard	Poultry	112	Depopulation completed 4/28 (CO2)	$3,250	6/1/2022	
4/26/2022	4/27/2022	Nebraska	Knox 01	Commercial Table Egg Layer	Chicken	2,117,993	Depopulation completed 5/15 (VSD+ heat/CO2)	$6,788,440	6/17/2022	
4/26/2022	4/28/2022	Minnesota	Swift 04	Backyard	WOAH NP	16	Depopulation completed 4/27 (CO2)	$415	5/12/2022	
4/26/2022	4/27/2022	Pennsylvania	Lancaster 06	Commercial Broiler Breeder Pullets	Chicken	18,133	Depopulation completed 4/26 (Foam)	$94,475	5/19/2022	

4/26/2022	4/28/2022	Minnesota	Carver 01	Backyard	WOAH NP	21	Depopulation completed 4/28 (Mechanically assisted cervical dislocation)	$790	5/13/2022	
4/26/2022	4/27/2022	Michigan	Saginaw 01	Backyard	WOAH NP	75	Depopulation completed 4/27 (CO2)	$1,220	5/12/2022	
4/25/2022	4/26/2022	North Dakota	Richland 02	Backyard	Poultry	15	Depopulation completed 4/26 (Cervical dislocation)	$605	5/16/2022	
4/25/2022	4/26/2022	Pennsylvania	Lancaster 05	Commercial Table Egg Layer	Chicken	307,363	Depopulation completed 4/28 (VSD+ heat)	$682,750	7/14/2022	
4/25/2022	4/28/2022	Indiana	Johnson 01	Backyard	WOAH NP	43	Depopulation completed 4/27 (Euthanasia/cervical dislocation/CO2)	$1,345	5/12/2022	
4/23/2022	4/23/2022	Wisconsin	Polk 02	Backyard	WOAH NP	40	Depopulation completed 4/24 (CO2)	$490	5/13/2022	
4/23/2022	4/25/2022	Michigan	Menominee 04	Backyard	WOAH NP	35	Depopulation completed 4/25 (CO2)	$1,105	5/10/2022	
4/22/2022	4/26/2022	Iowa	Kossuth 01	Backyard	WOAH NP	4	Depopulation completed 4/23 (CO2)	$0	5/8/2022	Owner is not pursuing indemnity.
4/22/2022	4/26/2022	Montana	Missoula 01	Backyard	WOAH NP	74	Depopulation completed 4/25 (Cervical dislocation)	$2,120	6/2/2022	
4/22/2022	4/25/2022	Minnesota	Rice 01	Backyard	WOAH NP	87	Depopulation completed 4/23 (Mechanically assisted cervical dislocation)	$5,139	5/11/2022	
4/22/2022	4/25/2022	Minnesota	Stearns 08	Commercial Turkey Meat Bird	Turkey	14,580	Depopulation completed 4/23 (Foam)	$272,210	5/25/2022	
4/21/2022	4/25/2022	Utah	Cache 01	Commercial Table Egg Layer	Chicken	1,501,181	Depopulation completed 5/13 (VSD+ heat/CO2)	$4,861,585	6/30/2022	
4/20/2022	4/22/2022	Minnesota	Yellow Medicine 02	Commercial Turkey Meat Bird	Turkey	49,501	Depopulation completed 4/22 (Foam)	$580,080	5/19/2022	
4/20/2022	4/21/2022	North Dakota	Renville 01	Backyard	WOAH NP	73	Depopulation completed 4/21 (Cervical dislocation)	$785	5/6/2022	
4/20/2022	4/21/2022	North Dakota	Richland 01	Commercial Turkey Meat Bird	Turkey	27,500	Depopulation completed 4/22 (VSD+ heat/Foam)	$592,130	5/16/2022	
Sample sent directly to NVSL	4/20/2022	Idaho	Madison 01	Backyard	Poultry	201	Depopulation completed 4/21 (CO2)	$5,870	5/6/2022	

4/20/2022	4/21/2022	Iowa	Bremer 01	Commercial Turkey Meat Bird	Turkey	29,150	Depopulation completed 4/21 (Foam)	$517,900	5/19/2022	
4/20/2022	4/22/2022	Pennsylvania	Lancaster 04	Commercial Broiler Production	Chicken	50,319	Depopulation completed 4/21 (VSD+ heat)	$105,750	5/12/2022	
4/20/2022	4/22/2022	Minnesota	Swift 03	Commercial Turkey Meat Bird	Turkey	127,389	Depopulation completed 4/23 (VSD+ heat)	$3,834,410	5/24/2022	
4/20/2022	4/22/2022	Minnesota	Otter Tail 04	Commercial Turkey Meat Bird	Turkey	27,274	Depopulation completed 4/21 (VSD+ heat)	$820,945	5/15/2022	
4/19/2022	4/21/2022	Minnesota	Todd 05	Commercial Turkey Meat Bird	Turkey	37,038	Depopulation completed 4/20 (Foam)	$492,985	6/2/2022	
4/19/2022	4/20/2022	Montana	Glacier 01	Backyard	Poultry	54,200	Depopulation completed 4/27 (CO2)	$211,055	6/9/2022	
4/19/2022	4/19/2022	North Dakota	Stutsman 02	Backyard	Poultry	19	Depopulation completed 4/17 (Producer)	$0	5/4/2022	Owner is not pursuing indemnity.
4/19/2022	4/21/2022	Minnesota	Morrison 11	Commercial Turkey Meat Bird	Turkey	40,863	Depopulation completed 4/20 (Foam/KEDS)	$574,925	5/17/2022	
Sample sent directly to NVSL	4/19/2022	Idaho	Gooding 02	Backyard	WOAH NP	78	Depopulation completed 4/20 (CO2)	$1,215	5/5/2022	
4/19/2022	4/21/2022	Minnesota	Todd 04	Commercial Turkey Meat Bird	Turkey	19,650	Depopulation completed 4/20 (Foam)	$262,230	5/22/2022	
4/19/2022	4/20/2022	Pennsylvania	Lancaster 03	Commercial Table Egg Layer	Chicken	879,428	Depopulation completed 4/24 (VSD+ heat)	$4,418,670	7/1/2022	
4/19/2022	4/20/2022	Pennsylvania	Lancaster 02	Commercial Table Egg Layer	Chicken	1,127,661	Depopulation completed 4/23 (VSD+ heat)	$2,038,435	6/25/2022	
4/18/2022	4/20/2022	Minnesota	Todd 03	Commercial Turkey Meat Bird	Turkey	30,286	Depopulation completed 4/19 (Foam)	$403,710	5/22/2022	
4/18/2022	4/20/2022	Minnesota	Todd 02	Commercial Turkey Meat Bird	Turkey	30,104	Depopulation completed 4/19 (Foam)	$401,285	5/22/2022	
4/18/2022	4/20/2022	Minnesota	Stearns 07	Commercial Turkey Meat Bird	Turkey	71,956	Depopulation completed 4/19 (Foam)	$1,343,420	5/19/2022	
4/18/2022	4/20/2022	Indiana	Elkhart 03	Commercial Duck Meat Bird	Duck	6,600	Depopulation completed 4/19 (Mechanically assisted cervical dislocation)	$5,895	5/5/2022	

4/17/2022	4/19/2022	Minnesota	Stearns 06	Commercial Turkey Meat Bird	Turkey	62,816	Depopulation completed 4/19 (VSD+ heat/Captive bolt)	$1,890,760	5/24/2022	
4/17/2022	4/19/2022	Minnesota	Morrison 10	Commercial Turkey Meat Bird	Turkey	47,716	Depopulation completed 4/18 (Foam)	$636,055	5/26/2022	
4/16/2022	4/19/2022	Colorado	La Plata 01	Backyard	Poultry	37	Depopulation completed 4/17 (CO2)	$735	5/3/2022	
4/16/2022	4/19/2022	Colorado	Montrose 01	Commercial Broiler Breeder	Chicken	58,044	Depopulation completed 4/22 (CO2)	$315,360	5/27/2022	
4/16/2022	4/19/2022	Minnesota	Swift 02	Commercial Turkey Meat Bird	Turkey	31,602	Depopulation completed 4/17 (Foam)	$2,248,800	5/20/2022	
4/16/2022	4/19/2022	Minnesota	Otter Tail 03	Commercial Turkey Meat Bird	Turkey	29,661	Depopulation completed 4/17 (VSD+ heat/Mechanically assisted cervical dislocation)	$892,795	5/24/2022	
4/15/2022	4/19/2022	Minnesota	Meeker 04	Commercial Turkey Meat Bird	Turkey	53,026	Depopulation completed 4/16 (Foam/Mechanically assisted cervical dislocation)	$1,596,085	5/24/2022	
4/14/2022	4/15/2022	Utah	Utah 01	Backyard	WOAH NP	7	Depopulation completed 4/14 (Injectable)	$0	5/18/2022	Owner is not pursuing indemnity.
4/15/2022	4/19/2022	Minnesota	Morrison 08	Commercial Turkey Meat Bird	Turkey	58,132	Depopulation completed 4/16 (Foam)	$549,345	5/24/2022	
4/15/2022	4/19/2022	Minnesota	Morrison 09	Commercial Turkey Meat Bird	Turkey	17,590	Depopulation completed 4/16 (Foam)	$234,475	5/21/2022	
4/15/2022	4/19/2022	Minnesota	Kandiyohi 08	Commercial Turkey Meat Bird	Turkey	35,659	Depopulation completed 4/16 (Foam/Mechanically assisted cervical dislocation)	$336,980	5/22/2022	
4/15/2022	4/16/2022	Michigan	Menominee 03	Backyard	WOAH NP	25	Depopulation completed 4/18 (Injectable)	$400	5/5/2022	
4/13/2022	4/15/2022	Wisconsin	Polk 01	Commercial Turkey Meat Bird	Turkey	22,000	Depopulation completed 4/14 (Foam)	$496,460	4/30/2022	
4/14/2022	4/15/2022	Michigan	Livingston 01	Backyard	WOAH NP	20	Depopulation completed 4/15 (CO2)	$240	5/5/2022	
4/13/2022	4/14/2022	Wisconsin	Columbia 01	Backyard	WOAH NP	65	Depopulation completed 4/14 (CO2/gunshot)	$1,005	4/29/2022	
4/13/2022	4/14/2022	Wisconsin	Sheboygan 01	Backyard	WOAH NP	16	Depopulation completed 4/14 (CO2)	$480	4/29/2022	

4/14/2022	4/19/2022	Minnesota	Kandiyohi 07	Commercial Turkey Meat Bird	Turkey	38,723	Depopulation completed 4/15 (Foam)	$953,360	5/23/2022	Same owner as Kandi 01 and 02.
4/14/2022	4/14/2022	Idaho	Gooding 01	Backyard	Poultry	20	Depopulation completed 4/15 (CO2)	$495	4/30/2022	
4/14/2022	4/14/2022	Idaho	Caribou 01	Backyard	WOAH NP	7	All birds died 4/15. No depopulation occurred.	$0	4/30/2022	Owner is not pursuing indemnity.
4/14/2022	4/15/2022	Pennsylvania	Lancaster 01	Commercial Table Egg Layer	Chicken	1,380,461	Depopulation completed 4/17 (VSD+ heat)	$4,918,430	6/1/2022	
4/13/2022	4/15/2022	Michigan	Macomb 02	Backyard	WOAH NP	42	Depopulation completed 4/14 (CO2)	$570	5/2/2022	
4/13/2022	4/14/2022	North Dakota	LaMoure 03	Backyard	WOAH NP	12	All birds died 4/15. No depopulation occurred.	$245	5/6/2022	
4/13/2022	4/14/2022	Minnesota	Blue Earth 02	Commercial Turkey Meat Bird	Turkey	26,385	Depopulation completed 4/13 (Foam)	$249,340	5/16/2022	
4/12/2022	4/14/2022	Minnesota	Meeker 03	Commercial Turkey Meat Bird	Turkey	73,201	Depopulation completed 4/13 (Foam)	$691,750	5/23/2022	
4/12/2022	4/13/2022	Minnesota	Otter Tail 02	Commercial Turkey Meat Bird	Turkey	35,723	Depopulation completed 4/13 (Foam)	$476,190	6/4/2022	
4/12/2022	4/14/2022	Indiana	Elkhart 02	Commercial Duck Breeder	Duck	6,379	Depopulation completed 4/13 (Mechanically assisted cervical dislocation/Mechanically assisted cervical dislocation)	$36,745	5/5/2022	
4/12/2022	4/13/2022	South Dakota	Deuel 01	Commercial Upland Gamebird Producer	Pheasant	1,392	Depopulation completed 4/14 (CO2/cervical dislocation)	$36,045	4/30/2022	
4/13/2022	4/13/2022	North Dakota	Barnes 01	Backyard	Poultry	90	Depopulation completed 4/12 (Cervical dislocation)	$0	5/12/2022	Owner is not pursuing indemnity.
4/12/2022	4/13/2022	Michigan	Menominee 02	Backyard	Poultry	351	Depopulation completed 4/13 (CO2)	$9,880	5/6/2022	Epi-link to Menominee 01.
4/11/2022	4/12/2022	North Carolina	Wayne 06	Commercial Broiler Production	Chicken	89,702	Depopulation completed 4/12 (Foam)	$278,973	5/13/2022	
4/11/2022	4/12/2022	Nebraska	Dixon 01	Commercial Table Egg Layer	Chicken	1,746,863	Depopulation completed 4/22 (VSD+ heat/CO2)	$5,548,615	5/9/2022	

4/11/2022	4/13/2022	Minnesota	Benton 02	Backyard	WOAH NP	7	Depopulation completed 4/12 (Mechanically assisted cervical dislocation)	$345	4/27/2022	
4/11/2022	4/12/2022	North Dakota	Sheridan 01	Backyard	WOAH NP	30	All birds died 4/16. No depopulation occurred.	$660	5/4/2022	
4/11/2022	4/13/2022	Minnesota	Waseca 03	Commercial Turkey Meat Bird	Turkey	20,338	Depopulation completed 4/12 (VSD+ heat/cervical dislocation)	$612,175	5/21/2022	
4/11/2022	4/13/2022	Minnesota	Blue Earth 01	Commercial Turkey Meat Bird	Turkey	22,727	Depopulation completed 4/12 (Foam)	$214,770	5/16/2022	
4/7/2022	4/8/2022	Colorado	Pitkin 01	Backyard	Poultry	34	Depopulation completed 4/8 (Cervical dislocation)	$0	4/22/2022	Owner is not pursuing indemnity.
4/10/2022	4/12/2022	Minnesota	Morrison 07	Commercial Table Egg Layer	Chicken	216,236	Depopulation completed 4/15 (VSD+ heat/CO2)	$653,765	6/16/2022	
4/10/2022	4/12/2022	Minnesota	Morrison 06	Commercial Broiler Production	Chicken	42,905	Depopulation completed 4/11 (VSD+ heat)	$169,905	5/13/2022	
	No Testing - Dangerous Contact	Minnesota	Todd 01	Commercial Turkey Meat Bird	Turkey	7,425	Depopulation completed 4/10 (Foam)	$138,625		Dangerous Contact for Morrison 05.
4/9/2022	4/13/2022	Kansas	McPherson 01	Commercial Turkey Breeder Hens	Turkey	6,861	Depopulation completed 4/11 (Foam)	$644,865	5/4/2022	
4/9/2022	4/12/2022	Montana	Toole 01	Backyard	WOAH NP	21	Depopulation completed 4/11 (Cervical dislocation)	$0	5/6/2022	Owner is not pursuing indemnity.
4/9/2022	4/12/2022	Minnesota	Yellow Medicine 01	Commercial Turkey Meat Bird	Turkey	44,750	Depopulation completed 4/12 (VSD+ heat)	$835,485	5/19/2022	
4/9/2022	4/12/2022	Minnesota	Morrison 05	Commercial Turkey Meat Bird	Turkey	37,642	Depopulation completed 4/10 (Foam)	$702,775	5/26/2022	
4/9/2022	4/12/2022	Minnesota	Kandiyohi 06	Commercial Turkey Meat Bird	Turkey	37,115	Depopulation completed 4/11 (Foam)	$1,117,160	5/21/2022	
4/8/2022	4/9/2022	South Dakota	Yankton 01	Commercial Turkey Meat Bird	Turkey	76,050	Depopulation completed 4/9 (VSD+heat/foam)	$1,580,245	4/27/2022	
4/9/2022	4/12/2022	Minnesota	Benton 01	Backyard	WOAH NP	126	Depopulation completed 4/12 (Gunshot)	$1,860	4/27/2022	
4/8/2022	4/12/2022	Wisconsin	Barron 01	Commercial Turkey Meat Bird	Turkey	46,507	Depopulation completed 4/10 (Foam)	$1,395,015	6/6/2022	

4/8/2022	4/11/2022	Michigan	Menominee 01	Backyard	WOAH NP	67	Depopulation completed 4/12 (CO2)	$1,700	4/27/2022	
4/7/2022	4/9/2022	Minnesota	Le Sueur 02	Commercial Turkey Meat Bird	Turkey	37,839	Depopulation completed 4/8 (Foam)	$1,138,955	5/7/2022	
4/7/2022	4/8/2022	Indiana	Elkhart 01	Commercial Duck Breeder	Duck	4,724	Depopulation completed 4/8 (Mechanically assisted cervical dislocation)	$26,755	5/5/2022	
4/7/2022	4/8/2022	South Dakota	Edmunds 04	Commercial Turkey Meat Bird	Turkey	36,377	Depopulation completed 4/8 (VSD+ heat/foam)	$809,455	5/5/2022	
4/6/2022	4/7/2022	Montana	Cascade 01	Backyard	Poultry	22,000	Depopulation completed 4/9 (CO2)	$86,345	5/19/2022	
4/7/2022	4/9/2022	Minnesota	Swift 01	Commercial Turkey Meat Bird	Turkey	130,929	Depopulation completed 4/5 (VSD+ heat)	$3,223,470	5/24/2022	
4/7/2022	4/9/2022	Minnesota	Stearns 05	Commercial Turkey Meat Bird	Turkey	26,145	Depopulation completed 4/8 (Foam)	$643,690	5/19/2022	
4/7/2022	4/8/2022	North Dakota	Stutsman 01	Backyard	Poultry	38	Depopulation completed 4/8 (CO2)	$2,235	5/12/2022	
4/6/2022	4/8/2022	Minnesota	Renville 01	Commercial Turkey Meat Bird	Turkey	76,381	Depopulation completed 4/8 (Foam)	$642,460	5/11/2022	
4/7/2022	4/8/2022	Minnesota	Otter Tail 01	Commercial Turkey Meat Bird	Turkey	29,873	Depopulation completed 4/7 (Foam)	$735,475	5/24/2022	
4/5/2022	4/6/2022	Missouri	Dade 01	Commercial Turkey Meat Bird	Turkey	14,000	Depopulation completed 4/7 (Foam)	$340,960	4/28/2022	
4/6/2022	4/7/2022	North Carolina	Wayne 05	Commercial Turkey Meat Bird	Turkey	18,546	Depopulation completed 4/7 (Foam)	$558,235	5/5/2022	
Sample sent directly to NVSL	4/6/2022	New York	Fulton 01	Backyard	WOAH NP	4	Depopulation completed 4/8 (Mechanically assisted cervical dislocation)	$105	4/29/2022	
Sample sent directly to NVSL	4/7/2022	Wisconsin	Racine 01	Backyard	Poultry	218	Depopulation completed 4/7 (Mechanically assisted cervical dislocation)	$7,690	4/22/2022	
4/6/2022	4/8/2022	South Dakota	Clark 03	Commercial Turkey Meat Bird	Turkey	38,678	Depopulation completed 4/7 (VSD+ heat/foam)	$835,550	4/28/2022	
4/4/2022	4/6/2022	Wyoming	Park 02	Backyard	WOAH NP	100	Depopulation completed 4/7 (CO2)	$620	4/25/2022	
4/5/2022	4/7/2022	Montana	Judith Basin 01	Backyard	WOAH NP	15	Depopulation completed 4/9 (Cervical dislocation)	$0	5/6/2022	Owner is not pursuing indemnity.

4/5/2022	4/6/2022	South Dakota	McPherson 04	Commercial Turkey Meat Bird	Turkey	45,900	Depopulation completed 4/7 (VSD+ heat)	$821,325	5/9/2022	
Sample sent directly to NVSL	4/5/2022	New York	Orleans 01	Backyard	Poultry	31	Depopulation completed 4/7 (CO2/cervical dislocation)	$90	5/10/2022	
4/5/2022	4/7/2022	Minnesota	Kandiyohi 05	Commercial Turkey Breeder Hens	Turkey	20,808	Depopulation completed 4/8 (Foam/cervical dislocation)	$1,951,380	5/16/2022	
4/4/2022	4/5/2022	Missouri	Lawrence 02	Commercial Turkey Meat Bird	Turkey	27,633	Depopulation completed 4/6 (Foam)	$829,855	5/6/2022	
Sample sent directly to NVSL	4/5/2022	Maine	Waldo 01	Backyard	WOAH NP	47	Depopulation completed 4/6 (CO2)	$2,270	4/20/2022	
4/5/2022	4/6/2022	Minnesota	Waseca 02	Commercial Turkey Meat Bird	Turkey	17,895	Depopulation completed 4/6 (Foam/Captive Bolt)	$538,640	5/21/2022	
4/4/2022	4/5/2022	South Dakota	Beadle 03	Commercial Turkey Meat Bird	Turkey	55,571	Depopulation completed 4/7 (VSD+ heat/foam)	$1,141,190	4/23/2022	
4/4/2022	4/5/2022	Minnesota	Big Stone 01	Commercial Turkey Meat Bird	Turkey	49,501	Depopulation completed 4/6/ (VSD+ heat/foam)	$1,050,665	5/11/2022	
4/4/2022	4/5/2022	Minnesota	Morrison 03	Commercial Turkey Meat Bird	Turkey	26,210	Depopulation completed 4/5 (Foam)	$360,695	5/23/2022	
4/4/2022	4/5/2022	Minnesota	Meeker 02	Commercial Turkey Meat Bird	Turkey	128,488	Depopulation completed 4/8 (VSD+ heat)	$3,867,490	5/23/2022	
4/4/2022	4/6/2022	North Carolina	Wayne 03	Commercial Broiler Production	Chicken	216,049	Depopulation completed 4/6 (Foam)	$671,912	4/29/2022	
4/4/2022	4/5/2022	Minnesota	Morrison 04	Commercial Turkey Meat Bird	Turkey	105,335	Depopulation completed 4/6 (Foam)	$1,186,755	5/25/2022	
4/4/2022	4/5/2022	Minnesota	Waseca 01	Commercial Turkey Meat Bird	Turkey	25,463	Depopulation completed 4/5 (Foam/captive bolt)	$766,435	5/20/2022	
4/4/2022	4/6/2022	North Carolina	Wayne 04	Commercial Broiler Production	Chicken	65,601	Depopulation completed 4/6 (Foam)	$341,781	5/9/2022	
4/4/2022	4/5/2022	Minnesota	Stearns 04	Commercial Turkey Meat Bird	Turkey	40,794	Depopulation completed 4/7 (VSD+ heat/foam)	$816,180	5/19/2022	
4/4/2022	4/5/2022	Iowa	Hardin 01	Commercial Turkey Meat Bird	Turkey	46,000	Depopulation completed 4/6 (VSD+ heat/foam)	$1,190,060	5/17/2022	

4/4/2022	4/5/2022	South Dakota	Clark 02	Commercial Turkey Meat Bird	Turkey	24,044	Depopulation completed 4/6 (VSD+ heat/foam)	$475,675	5/5/2022	
4/4/2022	4/5/2022	South Dakota	Spink 03	Commercial Turkey Meat Bird	Turkey	26,262	Depopulation completed 4/6 (VSD+ heat/foam)	$570,520	4/28/2022	
4/1/2022	4/5/2022	Wyoming	Sheridan 01	Backyard	WOAH NP	45	Depopulation completed 4/5 (CO2)	$0	4/25/2022	Owner is not pursuing indemnity.
4/3/2022	4/4/2022	Minnesota	Kandiyohi 04	Commercial Turkey Breeder Hens	Turkey	20,369	Depopulation completed 4/5 (Foam)	$1,824,040	5/17/2022	
4/3/2022	4/4/2022	South Dakota	McPherson 03	Commercial Turkey Meat Bird	Turkey	61,043	Depopulation completed 4/5 (VSD+ heat)	$1,292,880	4/26/2022	
4/3/2022	4/5/2022	North Carolina	Wayne 02	Commercial Turkey Meat Bird	Turkey	14,175	Depopulation completed 4/4 (Foam)	$426,668	5/9/2022	
4/3/2022	4/5/2022	South Dakota	Faulk 01	Commercial Turkey Meat Bird	Turkey	48,005	Depopulation completed 4/6 (VSD+ heat)	$1,102,825	4/29/2022	
4/3/2022	4/4/2022	Minnesota	Morrison 02	Commercial Turkey Meat Bird	Turkey	32,370	Depopulation completed 4/5 (Foam)	$642,675	5/20/2022	
4/3/2022	4/3/2022	Minnesota	Kandiyohi 03	Commercial Turkey Breeder Hens	Turkey	48,505	Depopulation completed 4/4 (Foam/Cervical dislocation)	$4,142,810	5/19/2022	
4/3/2022	4/3/2022	Minnesota	Dodge 01	Commercial Turkey Meat Bird	Turkey	19,971	Depopulation completed 4/3 (Foam)	$601,125	5/7/2022	
4/3/2022	4/3/2022	Minnesota	Becker 01	Commercial Turkey Meat Bird	Turkey	44,681	Depopulation completed 4/6 (Foam)	$968,470	6/3/2022	
4/2/2022	4/3/2022	Minnesota	Le Sueur 01	Commercial Turkey Meat Bird	Turkey	19,563	Depopulation completed 4/3 (foam)	$481,640	5/8/2022	
4/2/2022	4/5/2022	North Dakota	Cass 01	Backyard	WOAH NP	105	Depopulation completed 4/6 (CO2)	$1,850	5/4/2022	
4/2/2022	4/4/2022	North Dakota	LaMoure 02	Commercial Turkey Meat Bird	Turkey	50,850	Depopulation completed 4/5 (VSD+ heat/foam)	$1,089,400	5/6/2022	
4/2/2022	4/3/2022	Minnesota	Kandiyohi 02	Commercial Turkey Meat Bird	Turkey	38,525	Depopulation completed 4/4 (Foam/Captive bolt)	$948,485	5/23/2022	
4/1/2022	4/2/2022	Texas	Erath 01	Commercial Upland Gamebird Producer	Pheasant	1,689	Depopulation completed 4/2 (CO2)	$18,300	4/27/2022	

Sample sent directly to NVSL	4/2/2022	Iowa	Sac 01	Commercial Turkey Meat Bird	Turkey	37,240	Depopulation completed 4/3 (VSD+ heat)	$1,117,070	4/27/2022	
4/2/2022	4/2/2022	North Carolina	Wayne 01	Commercial Turkey Meat Bird	Turkey	16,924	Depopulation completed 4/2 (Foam)	$513,837	5/6/2022	
4/1/2022	4/2/2022	Minnesota	Stearns 03	Commercial Turkey Meat Bird	Turkey	27,556	Depopulation completed 4/2 (VSD+ heat/Foam)	$514,470	5/18/2022	
4/1/2022	4/5/2022	Nebraska	Scotts Bluff 01	Backyard	WOAH NP	33	Depopulation completed 4/3 (Injectable)	$635	4/21/2022	
4/1/2022	4/2/2022	South Dakota	Spink 02	Commercial Turkey Meat Bird	Turkey	41,773	Depopulation completed 4/4 (VSD+ heat/foam)	$933,905	4/28/2022	
4/1/2022	4/2/2022	Iowa	Humboldt 01	Commercial Table Egg Breeder	Chicken	15,268	Depopulation completed 4/3 (VSD+ heat)	$110,350	4/27/2022	
4/1/2022	4/2/2022	South Dakota	Lake 01	Commercial Turkey Meat Bird	Turkey	59,049	Depopulation completed 4/4 (VSD+, heat/foam)	$1,257,025	4/28/2022	
Sample sent directly to NVSL	4/1/2022	Wisconsin	Rock 01	Backyard	WOAH NP	22	Depopulation completed 4/2 (CO_2)	$200	4/20/2022	
4/1/2022	4/2/2022	North Dakota	LaMoure 01	Commercial Turkey Meat Bird	Turkey	27,400	Depopulation completed 4/4 (VSD+ heat/Foam)	$586,655	5/6/2022	
3/31/2022	4/2/2022	North Carolina	Johnston 02	Commercial Turkey Meat Bird	Turkey	9,546	Depopulation completed 4/2 (Foam)	$287,335	4/29/2022	
3/31/2022	4/2/2022	North Carolina	Johnston 03	Commercial Turkey Meat Bird	Turkey	18,888	Depopulation completed 4/1 (Foam)	$565,970	4/29/2022	
4/1/2022	4/1/2022	South Dakota	Charles Mix 04	Commercial Turkey Meat Bird	Turkey	81,534	Depopulation completed 4/2 (VSD+ heat/foam)	$1,210,990	4/22/2022	
3/31/2022	4/1/2022	Missouri	Jasper 02	Commercial Turkey Meat Bird	Turkey	35,321	Depopulation completed 4/1 (Foam)	$1,063,160	4/29/2022	
3/31/2022	4/4/2022	Iowa	Hamilton 02	Commercial Turkey Poult Supplier	Turkey	8,086	Depopulation completed 4/2 (VSD+ heat)	$76,410	4/20/2022	
Sample sent directly to NVSL	3/31/2022	Iowa	Osceola 01	Commercial Table Egg Layer	Chicken	5,011,726	Depopulation completed 4/16 (VSD+ heat/CO_2)	$14,739,450	6/9/2022	

Sample sent directly to NVSL	3/31/2022	Iowa	Cherokee 01	Commercial Turkey Meat Bird	Turkey	88,039	Depopulation completed 4/1 (VSD+ heat)	$2,620,930	5/8/2022	
Sample sent directly to NVSL	3/31/2022	Illinois	Carroll 01	Backyard	WOAH NP	38	Depopulation completed 4/4 (Cervical dislocation)	$255	5/2/2022	
Sample sent directly to NVSL	3/31/2022	North Dakota	Dickey 01	Backyard	Poultry	30	Depopulation completed 4/1 (CO2)	$925	5/4/2022	
3/31/2022	4/1/2022	North Dakota	Dickey 02	Commercial Turkey Meat Bird	Turkey	60,736	Depopulation completed 4/1(CO2)	$761,780	5/4/2022	
3/31/2022	3/31/2022	Minnesota	Morrison 01	Commercial Turkey Meat Bird	Turkey	52,623	Depopulation completed 3/31 (Foam)	$1,113,760	5/13/2022	
3/31/2022	4/1/2022	South Dakota	Edmunds 03	Commercial Turkey Breeder Replacement Hens	Turkey	30,569	Depopulation completed 4/2 (foam)	$3,074,920	4/25/2022	
3/30/2022	4/1/2022	Wyoming	Fremont 01	Backyard	WOAH NP	50	Depopulation completed 4/5 (CO2)	$350	4/22/2022	
3/28/2022	3/30/2022	Wyoming	Park 01	Backyard	WOAH NP	40	Depopulation completed 3/28 (Mechanically assisted cervical dislocation)	$0	4/18/2022	Owner is not pursuing indemnity.
3/30/2022	3/31/2022	South Dakota	McPherson 02	Commercial Turkey Breeder Hens	Turkey	26,048	Depopulation completed 4/1 (CO2)	$2,327,175	4/25/2022	
3/30/2022	3/31/2022	Minnesota	Stearns 02	Backyard	WOAH NP	146	Depopulation completed 3/31 (Cervical dislocation/captive bolt)	$4,700	5/7/2022	
3/29/2022	3/30/2022	Maine	Lincoln 03	Backyard	WOAH NP	3	Depopulation completed 3/30 (Injectable)	$100	4/13/2022	
3/28/2022	3/29/2022	Ohio	Franklin 01	Backyard	WOAH NP	8	Depopulation completed 3/20 (CO2)	$245	4/22/2022	
3/29/2022	3/30/2022	Iowa	Buena Vista 04	Commercial Turkey Meat Bird	Turkey	35,638	Depopulated completed 3/30 (VSD+ heat)	$698,940	5/1/2022	
Sample sent directly to NVSL	3/29/2022	Wyoming	Johnson 01	Backyard	WOAH NP	38	Depopulation completed 3/29 (Euthanasia)	$0	4/20/2022	

3/29/2022	3/29/2022	New York	Monroe 01	Backyard	Poultry	400	Depopulation completed 4/1 (CO2)	$6,880	5/5/2022	
3/29/2022	3/30/2022	South Dakota	Brule 01	Commercial Turkey Meat Bird	Turkey	55,066	Depopulation completed 3/31 (VSD+ heat/foam)	$1,253,535	4/21/2022	
3/28/2022	3/29/2022	Minnesota	Lac Qui Parle 01	Commercial Turkey Meat Bird	Turkey	22,507	Depopulation completed 3/30 (VSD+ heat/captive bolt)	$677,460	5/15/2022	
3/28/2022	3/29/2022	North Dakota	Kidder 01	Backyard	Poultry	27	Depopulation completed 4/8 (CO2)	$635	5/4/2022	
3/27/2022	3/29/2022	North Carolina	Johnston 01	Commercial Turkey Meat Bird	Turkey	32,144	Depopulation completed 3/28 (Foam)	$967,535	4/29/2022	
3/28/2022	3/29/2022	South Dakota	Edmunds 02	Commercial Turkey Breeder Hens	Turkey	21,924	Depopulation completed 3/31 (VSD+heat)	$2,069,030	5/5/2022	
3/28/2022	3/29/2022	Maine	Knox 04	Backyard	WOAH NP	43	Depopulation completed 3/29 (CO2)	$1,070	4/12/2022	
Sample sent directly to NVSL	3/28/2022	Iowa	Guthrie 01	Commercial Table Egg Layer	Chicken	1,460,030	Depopulation completed 4/6 (VSD+ heat/CO2)	$3,767,210	6/10/2022	
Sample sent directly to NVSL	3/28/2022	Iowa	Hamilton 01	Commercial Turkey Meat Bird	Turkey	25,805	Depopulation completed 3/29 (Foam)	$491,330	4/24/2022	
3/28/2022	3/29/2022	Minnesota	Kandiyohi 01	Commercial Turkey Meat Bird	Turkey	39,465	Depopulation completed 3/30 (VSD+ heat)	$1,187,895	5/22/2022	
3/27/2022	3/29/2022	South Dakota	Spink 01	Commercial Turkey Meat Bird	Turkey	45,865	Depopulation completed 3/30 (VSD+ heat/foam)	$779,930	4/21/2022	
3/27/2022	3/29/2022	South Dakota	Bon Homme 02	Commercial Turkey Meat Bird	Turkey	48,191	Depopulation completed 3/30 (VSD+ heat/foam)	$1,021,245	4/19/2022	
3/25/2022	3/29/2022	Massachusetts	Berkshire 01	Backyard	WOAH NP	256	Depopulation completed 3/30 (CO2)	$9,780	4/14/2022	
3/26/2022	3/27/2022	South Dakota	Clark 01	Commercial Turkey Meat Bird	Turkey	43,136	Depopulation completed 3/28 (VSD+ heat/foam)	$913,405	4/21/2022	
3/26/2022	3/27/2022	South Dakota	McPherson 01	Commercial Turkey Meat Bird	Turkey	43,937	Depopulation completed 3/29 (VSD+ heat/foam)	$834,605	4/23/2022	
3/26/2022	3/27/2022	South Dakota	Hutchinson 03	Commercial Turkey Meat Bird	Turkey	39,218	Depopulation completed 3/28 (VSD+ heat/foam)	$851,205	4/19/2022	

Sample sent directly to NVSL	3/26/2022	Maine	Washington 01	Backyard	WOAH NP	18	Depopulation completed 3/28 (CO2)	$850	4/11/2022	
3/26/2022	3/26/2022	Minnesota	Stearns 01	Commercial Turkey Meat Bird	Turkey	23,461	Depopulation completed 3/27 (Foam/Mechanically assisted cervical dislocation/captive bolt)	$434,940	5/9/2022	
3/25/2022	3/25/2022	New York	Suffolk 03	Backyard	Poultry	285	Depopulation completed 3/29 (CO2)	$1,260	4/28/2022	
3/25/2022	3/26/2022	Missouri	Gentry 01	Backyard	WOAH NP	143	Depopulation completed 3/27 (CO2)	$3,990	4/11/2022	
3/25/2022	3/25/2022	Minnesota	Mower 01	Backyard	WOAH NP	20	Depopulation completed 3/26 (Mechanically assisted cervical dislocation)	$290	5/8/2022	
3/25/2022	3/26/2022	South Dakota	Bon Homme 01	Backyard	WOAH NP	226	Depopulation completed 3/27 (CO2)	$4,625	4/29/2022	
Sample sent directly to NVSL	3/25/2022	Iowa	Franklin 01	Commercial Table Egg Pullets	Chicken	250,239	Depopulation completed 3/27 (VSD+ heat)	$778,240	4/19/2022	
3/25/2022	3/25/2022	Minnesota	Meeker 01	Commercial Turkey Meat Bird	Turkey	287,507	Depopulation completed 3/28 (VSD+ heat/foam)	$8,653,960	5/18/2022	
3/25/2022	3/26/2022	Nebraska	Holt 01	Backyard	WOAH NP	53	Depopulation completed 3/26 (Injectable/ barbiturate/ cervical dislocation)	$430	4/13/2022	
3/24/2022	3/25/2022	Kansas	Mitchell 01	Backyard	WOAH NP	20	Depopulation completed 3/26 (CO2)	$440	4/11/2022	
3/24/2022	3/25/2022	South Dakota	Hutchinson 02	Commercial Turkey Meat Bird	Turkey	45,778	Depopulation completed 3/26 (VSD+ heat/foam)	$911,640	4/19/2022	
Sample sent directly to NVSL	3/23/2022	New York	Suffolk 02	Commercial Upland Gamebird Producer	Pheasant	8,507	Depopulation completed 3/30 (CO2/Mechanically assisted cervical dislocation/cervical dislocation)	$132,960	4/28/2022	
3/23/2022	3/24/2022	Nebraska	Butler 02	Commercial Broiler Production	Chicken	418,478	Depopulation completed 3/24 (VSD+ heat)	$1,657,175	4/13/2022	

3/23/2022	3/24/2022	South Dakota	Jerauld 01	Commercial Turkey Meat Bird	Turkey	44,033	Depopulation completed 3/26 (VSD+ heat/foam)	$892,745	4/15/2022	
3/23/2022	3/24/2022	Iowa	Buena Vista 03	Commercial Turkey Meat Bird	Turkey	53,804	Depopulation completed 3/24 (Foam/Mechanically assisted cervical dislocation)	$1,028,290	5/12/2022	
3/23/2022	3/24/2022	Michigan	Macomb 01	Backyard	WOAH NP	66	Depopulation completed 3/25 (injectable)	$1,880	4/21/2022	
3/22/2022	3/23/2022	South Dakota	Beadle 02	Commercial Turkey Meat Bird	Turkey	49,892	Depopulation completed 3/24 (VSD+ heat)	$1,117,430	4/20/2022	
3/22/2022	3/23/2022	South Dakota	Kingsbury 02	Commercial Table Egg Layer	Chicken	120,293	Depopulation completed 3/24 (CO2)	$310,595	4/20/2022	
3/22/2022	3/23/2022	South Dakota	Edmunds 01	Commercial Turkey Breeder Hens	Turkey	30,756	Depopulation completed 3/23 (VSD+ heat)	$2,151,080	5/5/2022	
Sample sent directly to NVSL	3/22/2022	Maine	Cumberland 01	Backyard	Poultry	247	Depopulation completed 3/23 (CO2)	$6,750	4/7/2022	
3/21/2022	3/22/2022	South Dakota	Beadle 01	Commercial Turkey Meat Bird	Turkey	29,413	Depopulation completed 3/24 (VSD+ heat)	$616,570	4/20/2022	
3/21/2022	3/22/2022	South Dakota	Hanson 02	Commercial Turkey Meat Bird	Turkey	94,585	Depopulation completed 3/24 (VSD+ heat/foam)	$2,052,873	4/14/2022	
3/21/2022	3/22/2022	South Dakota	Charles Mix 03	Commercial Turkey Meat Bird	Turkey	53,763	Depopulation completed 3/24 (VSD+ heat/foam)	$1,022,190	4/14/2022	
3/21/2022	3/22/2022	Nebraska	Butler 01	Commercial Broiler Production	Chicken	570,000	Depopulation completed 3/22 (VSD+)	$1,900,500	4/11/2022	
3/19/2022	3/20/2022	South Dakota	Hutchinson 01	Commercial Turkey Meat Bird	Turkey	50,350	Depopulation completed 3/23 (VSD+ heat/foam)	$1,392,360	4/19/2022	
3/19/2022	3/20/2022	Iowa	Warren 01	Backyard	WOAH NP	10	Depopulation completed 3/21 (CO2)	$95	4/5/2022	
Sample sent directly to NVSL	3/19/2022	Maine	Knox 03	Backyard	WOAH NP	19	Depopulation completed 3/21 (CO2)	$500	4/4/2022	
3/18/2022	3/19/2022	Maine	York 02	Backyard	WOAH NP	65	Depopulation completed 3/19 (CO2)	$1,650	4/2/2022	
3/17/2022	3/18/2022	South Dakota	Kingsbury 01	Commercial Turkey Meat Bird	Turkey	39,156	Depopulation completed 3/19 (VSD+ heat)	$1,095,835	4/20/2022	

3/17/2022	3/17/2022	Maine	Lincoln 02	Backyard	WOAH NP	29	Depopulation completed 3/18 (CO2)	$630	4/2/2022	
3/17/2022	3/18/2022	South Dakota	Hanson 01	Commercial Turkey Meat Bird	Turkey	20,233	Depopulation completed 3/19 (VSD+ heat/foam)	$427,620	4/19/2022	
3/17/2022	3/18/2022	Kansas	Dickinson 01	Backyard	Poultry	247	Depopulation completed 3/19 (CO2)	$8,365	4/4/2022	
Sample sent directly to NVSL	3/17/2022	Iowa	Buena Vista 02	Commercial Table Egg Layer	Chicken	5,347,511	Depopulation completed 3/24 (VSD+ heat/CO2)	$17,311,050	5/17/2022	
3/17/2022	3/17/2022	Kansas	Sedgwick 01	Backyard	WOAH NP	6	Depopulation completed 3/18 (Cervical dislocation)	$70	4/4/2022	
3/17/2022	3/18/2022	Maryland	Cecil 04	Commercial Table Egg Pullets	Chicken	315,399	Depopulation completed 3/20 (VSD+ heat/cervical dislocation)	$980,890	4/29/2022	
3/17/2022	3/16/2022	New Hampshire	Rockingham 01	Backyard	WOAH NP	151	Depopulation completed 3/18 (CO2/cervical dislocation)	$4,320	4/6/2022	
3/16/2022	3/17/2022	Delaware	Kent 01	Commercial Broiler Production	Chicken	147,753	Depopulated completed 3/17 (VSD+ heat/foam)	$585,100	4/13/2022	
3/15/2022	3/15/2022	Nebraska	Merrick 01	Backyard	WOAH NP	90	Depopulation completed 3/17 (CO2/Barbiturate)	$1,930	4/1/2022	
3/14/2022	3/15/2022	Missouri	Ralls 01	Backyard	WOAH NP	104	Depopulation completed 3/16 (CO2)	$3,140	3/31/2022	
3/14/2022	3/14/2022	Maine	York 01	Backyard	WOAH NP	184	Depopulation completed 3/16 (CO2)	$4,270	4/2/2022	
3/13/2022	3/14/2022	Wisconsin	Jefferson 01	Commercial Table Egg Layer	Chicken	2,750,706	Depopulation completed 3/30 (VSD+ heat/CO2)	$10,521,125	5/13/2022	
3/11/2022	3/12/2022	South Dakota	Charles Mix 02	Commercial Turkey Meat Bird	Turkey	37,485	Depopulation completed 3/13 (VSD+ heat/foam)	$785,535	4/13/2022	
3/11/2022	3/12/2022	Maine	Lincoln 01	Backyard	WOAH NP	98	Depopulation completed 3/13 (CO2)	$2,570	4/2/2022	
3/10/2022	3/11/2022	Illinois	McLean 01	Backyard	WOAH NP	35	Depopulation completed 3/12 (CO)	$760	3/28/2022	
3/10/2022	3/11/2022	Kansas	Franklin 01	Backyard	Poultry	264	Depopulation completed 3/12 (CO2)	$4,770	3/27/2022	

Sample sent directly to NVSL	3/10/2022	Iowa	Taylor 01	Commercial Table Egg Layer	Chicken	915,925	Depopulation completed 3/14 (VSD+ heat/CO2/cervical dislocation)	$3,622,800	5/13/2022	
3/8/2022	3/9/2022	Missouri	Lawrence 01	Commercial Turkey Breeder Replacement Hens	Turkey	37,770	Depopulation completed 3/9 (foam)	$3,171,445	4/5/2022	
3/7/2022	3/8/2022	Maryland	Queen Anne's 01	Commercial Broiler Production	Chicken	139,327	Depopulation completed 3/9 (VSD+ heat)	$725,895	4/1/2022	
3/7/2022	3/8/2022	Delaware	New Castle 02	Commercial Table Egg Pullets	Chicken	243,900	Depopulation completed 3/11 (VSD+ heat/CO2)	$943,895	4/29/2022	
3/7/2022	3/8/2022	Missouri	Jasper 01	Commercial Turkey Meat Bird	Turkey	25,042	Depopulation completed 3/9 (foam)	$753,765	4/12/2022	
3/6/2022	3/6/2022	Iowa	Buena Vista 01	Commercial Turkey Meat Bird	Turkey	49,816	Depopulation completed 3/7 (VSD+ heat)	$1,475,440	4/1/2022	
	No Testing - Dangerous Contact	Maryland	Cecil 03	Commercial Table Egg Processor	Chicken	0		$1,068,120		
3/4/2022	3/5/2022	South Dakota	Charles Mix 01	Commercial Turkey Meat Bird	Turkey	44,123	Depopulation completed 3/7 (VSD+ heat/foam)	$993,855	4/13/2022	
Sample sent directly to NVSL	3/4/2022	Missouri	Bates 01	Backyard	WOAH NP	51	Depopulation completed 3/5 (CO2)	$1,205	3/23/2022	
3/9/2022	3/10/2022	Maryland	Cecil 02	Commercial Table Egg Layer	Chicken	663,406	Depopulation completed 3/13 (VSD+/CO2)	$2,474,160	4/29/2022	
3/3/2022	3/4/2022	Maryland	Cecil 01	Commercial Table Egg Layer	Chicken	644,004	Depopulated completed 3/8 (VSD+ heat/CO2)	$1,658,110	4/29/2022	
3/3/2022	3/4/2022	Missouri	Stoddard 01	Commercial Broiler Production	Chicken	294,818	Depopulated completed 3/5 (VSD+ heat/foam)	$1,186,305	4/1/2022	
3/1/2022	3/2/2022	Indiana	Dubois 04	Commercial Turkey Meat Bird	Turkey	16,494	Depopulation completed 3/1 (foam)	$304,260	3/23/2022	
2/28/2022	3/1/2022	Iowa	Pottawattamie 01	Backyard	WOAH NP	42	Depopulation completed 3/1 (Mechanically assisted cervical dislocation)	$210	3/17/2022	

2/28/2022	3/1/2022	Connecticut	New London 01	Backyard	WOAH NP	162	Depopulation completed 3/1 (CO2/CED)	$4,810	3/18/2022	
2/23/2022	2/24/2022	New York	Ulster 01	Backyard	WOAH NP	65	Depopulation completed 2/28 (Mechanically assisted cervical dislocation)	$1,950	4/1/2022	
2/23/2022	2/24/2022	New York	Dutchess 01	Backyard	WOAH NP	195	Depopulation completed 2/26 (Cervical Dislocation)	$680	4/5/2022	
2/22/2022	2/23/2022	Michigan	Kalamazoo 01	Backyard	WOAH NP	34	Depopulation completed 2/24 (CO2/ Hunting)	$975	3/28/2022	
2/22/2022	2/24/2022	Indiana	Dubois 03	Commercial Turkey Meat Bird	Turkey	35,988	Depopulation completed 2/23 (Foam)	$1,080,830	3/23/2022	
2/22/2022	2/23/2022	Maine	Knox 02	Backyard	WOAH NP	96	Depopulation completed 2/23 (CO2)	$2,340	3/11/2022	
2/21/2022	2/22/2022	Delaware	New Castle 01	Commercial Table Egg Layer	Chicken	1,046,937	Depopulation completed 3/1 (CO2/VSD+)	$3,685,430	4/29/2022	
2/18/2022	2/23/2022	Indiana	Greene 02	Commercial Turkey Meat Bird	Turkey	15,400	Depopulation completed 2/19 (Foam)	$460,320	3/15/2022	
2/18/2022	2/19/2022	Maine	Knox 01	Backyard	WOAH NP	27	Depopulation completed 2/19 (CO2)	$410	3/11/2022	
2/18/2022	2/18/2022	New York	Suffolk 01	Backyard	WOAH NP	8	Depopulation completed 2/20 (Cervical dislocation)	$160	4/6/2022	
2/17/2022	2/23/2022	Indiana	Greene 01	Commercial Turkey Meat Bird	Turkey	48,211	Depopulation completed 2/18 (Foam)	$603,190	3/15/2022	
2/14/2022	2/16/2022	Indiana	Dubois 02	Commercial Turkey Meat Bird	Turkey	26,625	Depopulation completed 2/15 (Foam)	$651,765	3/23/2022	
2/13/2022	2/15/2022	Kentucky	Webster 01	Commercial Turkey Meat Bird	Turkey	53,286	Depopulation completed 2/15 (Foam/VSD+ heat)	$1,603,910	3/12/2022	
2/11/2022	2/12/2022	Kentucky	Fulton 01	Commercial Broiler Production	Chicken	231,398	Depopulation completed 2/15 (VSD+ heat/Foam/cervical dislocation)	$916,340	3/15/2022	
2/11/2022	2/12/2022	Virginia	Fauquier 01	Backyard	WOAH NP	90	Depopulation completed 2/12 (CO2)	$2,380	2/26/2022	
2/7/2022	2/8/2022	Indiana	Dubois 01	Commercial Turkey Meat Bird	Turkey	29,015	Depopulation completed 2/9 (Foam)	$870,340	3/23/2022	

Privacy Impact Assessment
Emergency Management Response Services 2.0

Technology, Planning, Architecture, & E-Government

- Version: 1.9
- Date: March 23 2023
- Prepared for: Marketing and Regulatory Programs

United States Department of Agriculture

Privacy Impact Assessment for the

Emergency Management Response Services 2.0 (EMRS2)

March 2023

<u>Contact Point</u>
Jonathan Zack, DVM
APHIS Veterinary Services
United States Department of Agriculture
(301) 851-3460

<u>Reviewing Official</u>
Tonya Woods
Director, Freedom of Information and Privacy Act Staff
United States Department of Agriculture
(301) 734-8296

Abstract

The Emergency Management Response Services 2.0 (EMRS2) is a Major Application used by the APHIS Veterinary Services (VS) to manage and investigate animal disease outbreaks in the United States. The system is used by Federal, State, Tribal, and local animal health officials and human health officials. This Privacy Impact Assessment (PIA) is being completed following the Privacy Threshold Analysis (PTA) conclusion requiring a PIA for EMRS2 to meet federal privacy compliance requirements.

Overview

The EMRS2 is an incident management data collection system is utilized by Veterinary Services to manage and investigate animal disease outbreaks and instances of foreign animal disease (FAD) in the United States. The EMRS2 business requirement has three main process domains: Investigation management, Lab Submission management, and Resource management.

EMRS2 is custom built within the Microsoft 365 platform and is accessed by approved users via Microsoft Internet Explorer. Primary users of EMRS2 are Federal and State veterinary medical officers, animal health technicians, and various disease specialists and epidemiologists from APHIS and from State cooperators.

There are two extensions to EMRS2 also in use which serve as alternate user interfaces but enforce all applicable security thru the Dynamics API:

1) *Gateway*
2) *EMRS2GO*

The Gateway allows producers to request and manage movement permits. Users do not receive EMRS2 accounts and cannot interact directly.

EMRS2GO is a Windows Presentation Foundation application that runs on the users' laptop. It exposes the Incident Contact Reports (ICR) and other forms to the user in an effort to minimize their entry errors. The EMRS2GO application, like the Gateway, leverages the Dynamics API.

Section 1.0 Characterization of the Information

The following questions are intended to define the scope of the information requested and/or collected as well as reasons for its collection as part of the program, system, rule, or technology being developed.

1.1 What information is collected, used, disseminated, or maintained in the system?

The system collects, uses and maintains information such as:

Owner or operator of the premises where the animals subject to investigation are located; the system includes the following information, such as, but not limited to, the name; address (including city, county, State, postal code, and latitude/longitude coordinates); premises identification number; and telephone number.

Referring contact information, which includes name and telephone number.

Case coordinator of the premises investigation; the system includes name, telephone number, and email address.

APHIS employees; the system includes the following information, such as, but not limited to the name; agency, program, and group; current duty assignment; encrypted employee identification number; grade, series, and step; duty city and State; home address, including latitude/longitude coordinates; home telephone number; home email address; emergency contact information; work and field addresses, email addresses and telephone numbers; and supervisor contact information.

1.2 What are the sources of the information in the system?

The sources of the information are customers defined as, owner or operator of the premises where the animal(s) subject to investigation are located; and from APHIS VS employees, referring contact, and case coordinator. Such information may be supplemented by other sources of information such as, an address-validation database, by APHIS personnel during an on-site investigation, by State and Tribal veterinarian offices and State laboratories, or by APHIS' National Veterinary Services Laboratories. Information may also be sourced from the Financial Management Modernization Incentive for payment status. Employee information is obtained primarily from the employee. Additionally, employee information may be obtained from the U.S. Department of Agriculture's (USDA's) National Finance Center, AgLearn database, and Federal Occupational Health, U.S. Department of Health and Human Services.

1.3 Why is the information being collected, used, disseminated, or maintained?

Data is used by VS to manage and investigate animal disease outbreaks in the United States. This information is required to process numerous documents such as those needed for indemnity to owners/growers, reimbursement for cleaning and disinfection, and record keeping for surveillance, testing. The system is used by Federal, State, Tribal, and local animal health officials (and human health officials) for:

- Routine reporting of Foreign Animal Disease (FAD) investigations
- Surveillance and control programs
- State-specific disease outbreaks
- National animal health emergency responses

1.4 How is the information collected?

Information is provided to APHIS, as well as State and Tribal officials to enter this information into the main EMRS2 application, or the Gateway (by producers/owners and growers) and EMRS2GO extensions when appropriate. State and local Veterinary Officers and various disease program laboratories provide data for use in EMRS2, depending upon the geographic extent of the animal disease outbreak, and dependent upon if an appropriate data sharing Memorandum of Understanding (MOU) is in place with USDA. The mapping module occasionally utilizes public data from the U.S. Geological Survey and other Federal resources available to the public.

1.5 How will the information be checked for accuracy?

Authorized federal, state, or EMRS2 personnel that collect and enter the data are responsible for the review and accuracy of the data. Information is obtained from either a customer or an employee and is often supplemented during an investigation by on-site visits, USPS database, or other address-validation databases. There are also limited data entry constraints to ensure entry completeness. APHIS employees also have access to the EMRS2 Administrative module where they may edit and maintain their own employee profiles. EMRS2 updates Employee Profiles via records in the Emergency Qualification System (EQS) that is shared using a flat file each quarter. When an employee profile changes, EMRS2 receives the updated information via the next scheduled EQS file share. (EQS gets their data from the National Finance Center (NFC) bi-weekly).

1.6 What specific legal authorities, arrangements, and/or agreements defined the collection of information?

APHIS is an emergency response organization whose mission is to protect the health and value of U.S. agricultural, natural and other resources.

The Animal Health Protection Act (AHPA) (7 U.S.C.8301 et seq.) provides the authority for the Secretary to prevent, detect, control, and eradicate diseases, and pests of birds and other livestock to protect animal health, the health and welfare of people, economic interests of livestock and related industries, the environment, and interstate and foreign commerce in birds, other livestock, and other articles. EMRS2 is the system used to act on this authority.

Any additional authority comes from the specific state under which investigation is occurring.

1.7 <u>Privacy Impact Analysis</u>: Given the amount and type of data collected, discuss the privacy risks identified and how they were mitigated.

Unauthorized access, inaccurate data and unauthorized use are privacy risks associated with the amount and type of data in EMRS2.

Unauthorized access risks are mitigated by the following means:

- Users accessing EMRS 2 must successfully authenticate using their e-Authentication PIV or e-Authentication username/password credential and be authorized with specific EMRS role(s).
- The application limits access to relevant information and prevents access to unauthorized information.
- Devices running the EMRS2GO extension must have a government approved encryption in place or the application will not run.
- Users of EMRS2GO must have an authorized account in the EMRS2 web application.

Data is secured by means of encryption and access control. Access is controlled by:

- User ID and password or PIV card
- e-Authentication
- Access Control list
- Read and write authorization permissions that are specific to individual EMRS2 electronic forms
- Microsoft Dynamics 365 role-based access control.

The accuracy of the customer information is confirmed with the customer prior to submission into the EMRS2. This helps to mitigate the risk of inaccurate information at the point of collection.

Section 2.0 Uses of the Information

The following questions are intended to delineate clearly the use of information and the accuracy of the data being used.

2.1 Describe all the uses of information.

Data is used by VS to manage and investigate animal disease outbreaks in the United States. The system is used by Federal, State, Tribal, and local animal health officials (and human health officials) for:

- Routine reporting of Foreign Animal Disease (FAD) investigations
- Animal disease surveillance and control programs
- State-specific animal disease outbreaks
- National animal health emergency responses

When other Federal and State emergency response agencies assist USDA with an emergency disease outbreak, they may be allowed limited access to the data in

EMRS2. The access will depend upon the MOU in place and the need to know of the other agency. Data will be used for:

- Routine reporting of FAD investigations
- Surveillance and control programs
- State-specific disease outbreaks
- National animal health emergency responses

2.2 What types of tools are used to analyze data and what type of data may be produced?

Microsoft Dynamics 365 includes customizations to allow users to visualize and understand data using GIS mapping to support situational awareness needs.

Dynamics 365 also includes features to allow users to analyze data in various ways. The most basic analysis tool is the view. Users may customize views to display data sorted by specific field and display only the data in selected fields. Users may only view the data to which they have access based on their role, as defined in Dynamics 365. Users may also create charts and graphs to show trends and statistical information. Users can create dashboards to display information that is customized to their needs.

2.3 If the system uses commercial or publicly available data please explain why and how it is used.

EMRS2 uses Bing Maps for imagery only and utilizes no other Bing Map services. These maps are needed to assess the geographical location of infected premises and at-risk premises within the control area and/or surveillance zone. Field responders utilize geographical coordinates to conduct surveillance activities in the affected area.

2.4 <u>Privacy Impact Analysis</u>: Describe any types of controls that may be in place to ensure that information is handled in accordance with the above described uses.

Data is secured by means of encryption and access control. Access is controlled by:

- User ID and password or PIV card
- e-Authentication
- Access Control list
- Read and write authorization permissions that are specific to individual EMRS2 electronic forms
- Microsoft Dynamics 365 role-based access control.

The VS management team and National Preparedness and Incident Coordination Center management will determine when data needs to be consolidated and ensure date is protected from unauthorized access.

Section 3.0 Retention

The following questions are intended to outline how long information will be retained after the initial collection.

3.1 How long is information retained?

As of today, records are retained permanently in accordance with unscheduled records management policy. Once scheduled and approved by NARA, all EMRS records will be retained for 50 years.

3.2 Has the retention period been approved by the component records officer and the National Archives and Records Administration (NARA)?

APHIS VS has developed record retention schedules, but until they are approved by NARA, electronic systems are classified as permanent in accordance with unscheduled records management policy.

3.3 Privacy Impact Analysis: Please discuss the risks associated with the length of time data is retained and how those risks are mitigated.

Risks associated with data retention are minimal and include the possibility of the data being accessed by unauthorized personnel. EMRS2 uses role-based access to mitigate this risk. The VS Leadership team and National Preparedness and Incident Coordination Center staff, State Veterinarians and EMRS2 team members and authorized users are all responsible for protecting the privacy rights of the customers and employees affected by the interface. The login interface reminds users of their responsibility every time they log in.

On mobile devices the mitigation above holds true: the EMRS2GO extension uses the user's EMRS2 account for authentication and authorization, such that they cannot gain any further access than they already have. Additionally, the mobile extension will only run on devices with an approved encryption and there are controls on the concurrency of the data related to last access of the application such that if the application is not used for a government-determined period, the application will be forced to synchronize, and in the event the EMRS account is no longer valid the sync will not return data and existing reference data will be wiped.

Disclosure or disposal of the data poses additional risks to this data, and this is mitigated by ensuring the implementation of technical controls such as auditing, access control and system communications; the implementation of operational controls like configuration management, contingency planning, system and security integrity, and the implementation of management controls such as annual risk assessments, planning and security assessment and authorization, are in place and operating as expected. These controls are explained in NIST Special Publication 800-53.

Section 4.0 Internal Sharing and Disclosure

The following questions are intended to define the scope of sharing within the United States Department of Agriculture.

4.1 With which internal organization(s) is the information shared, what information is shared and for what purpose?

EMRS2 does not share data with any internal organizations outside the parameters of standard user access.

4.2 How is the information transmitted or disclosed?

N/A

4.3 Privacy Impact Analysis: Considering the extent of internal information sharing, discuss the privacy risks associated with the sharing and how they were mitigated.

N/A

Section 5.0 External Sharing and Disclosure

The following questions are intended to define the content, scope, and authority for information sharing external to USDA which includes Federal, state and local government, and the private sector.

5.1 With which external organization(s) is the information shared, what information is shared, and for what purpose?

(1) To certain Federal, State, and Tribal animal health officials to identify premises before an event to allow for faster response, monitor the status of an animal disease investigation, document actions taken relating to an animal disease investigation, track the status of animals susceptible to foreign animal diseases, determine the costs of an animal disease investigation, monitor the use and availability of assets and personnel relating to animal disease investigations, or perform epidemiological and geospatial analyses of such investigations;

(2) To Federal, State, and Tribal animal health officials within the system to obtain feedback regarding the EMRS system and emergency preparedness guidelines, and to educate and involve them in program development, program requirements, and standards of conduct;

(3) When a record on its face, or in conjunction with other records, indicates a violation or potential violation of law, whether civil, criminal, or regulatory in nature, and whether arising by general statute or particular program, statute, or by regulation, rule, or order issued pursuant thereto, disclosure may be made to the appropriate agency, whether Federal, foreign, State, Tribal, local, or other public authority

responsible for enforcing, investigating, or prosecuting such violation or charged with enforcing or implementing the statute, rule, regulation, or order issued pursuant thereto, if the information disclosed is relevant to any enforcement, regulatory, investigative, or prosecutive responsibility of the receiving entity;

(4) To the Department of Justice when: (a) USDA or any component thereof; or (b) any employee of USDA in his or her official capacity, where the Department of Justice has agreed to represent the employee; or (c) the United States Government, is a party to litigation or has an interest in such litigation, and USDA determines that the records are relevant and necessary to the litigation and the use of such records by the Department of Justice is therefore deemed by USDA to be for a purpose that is compatible with the purpose for which USDA collected the records;

(5) In an appropriate proceeding before a court, grand jury, or administrative or adjudicative body or official, when USDA or other Agency representing USDA determines that the records are relevant and necessary to the proceeding; or in an appropriate proceeding before an administrative or adjudicative body when the adjudicator determines the records to be relevant to the proceeding; ;

(6) To appropriate agencies, entities, and persons when: (a) USDA suspects or has confirmed that the security or confidentiality of information in the system of records has been compromised; (b) USDA has determined that as a result of the suspected or confirmed breach there is a risk of harm to individuals, USDA (including its information systems, programs, and operations), the Federal Government, or national security; and (c) the disclosure made to such agencies, entities, and persons is reasonably necessary to assist in connection with USDA's efforts to respond to the suspected or confirmed compromise and prevent, minimize, or remedy such harm;

(7) To another Federal agency or Federal entity, when information from this system of records is reasonably necessary to assist the recipient agency or entity in (a) responding to a suspected or confirmed breach or (b) preventing, minimizing, or remedying the risk of harm to individuals, the agency (including its information systems, programs, and operations), the Federal Government, or national security;

(8) To contractors and their agents, grantees, experts, consultants, and others performing or working on a contract, service, grant, cooperative agreement, or other assignment for the USDA, when necessary to accomplish an agency function related to this system of records;);

(9) To Congressional office staff in response to an inquiry from that Congressional office made at the written request of the individual about whom the record pertains; and

(10) APHIS may disclose information in this system of records to the National Archives and Records Administration or to the General Services Administration for records management inspections being conducted under 44 U.S.C. 2904 and 2906.

5.2 Is the sharing of personally identifiable information outside the Department compatible with the original collection? If so, is it covered by an appropriate routine use in a SORN? If so, please

describe. If not, please describe under what legal mechanism the program or system is allowed to share the personally identifiable information outside of USDA.

Yes. The sharing of personally identifiable information outside the Department is compatible with the original collection.

System of Records Notice APHIS-11 Emergency Management Response System describes the applicable routine use that covers this external sharing of personally identifiable information.

5.3 How is the information shared outside the Department and what security measures safeguard its transmission?

Information shared outside the Department falls within the disclosures outlined in section 5.1. The data are extracted per the requested parameters and is then transmitted to the requesting internal point of contact using secure protocols and connections. The actual sharing to the external source is done by the USDA APHIS Privacy Act Office in the Legislative and Public Affairs (LPA) Branch.

5.4 Privacy Impact Analysis: Given the external sharing, explain the privacy risks identified and describe how they were mitigated.

Information shared outside the Department falls within the disclosures outlined in section 5.1. The data is extracted or transferred per specific parameters and is then transmitted to the requesting partner organizational system using secure protocols and connections. Where the external sharing is the result of a Freedom of Information Act request, the actual sharing to the external source is done by the USDA APHIS Privacy Act Office in the Legislative and Public Affairs (LPA) Branch.

Section 6.0 Notice

The following questions are directed at notice to the individual of the scope of information collected, the right to consent to uses of said information, and the right to decline to provide information.

6.1 Was notice provided to the individual prior to collection of information?

Yes.

6.2 Do individuals have the opportunity and/or right to decline to provide information?

Yes. There is no penalty at the federal level if user refuses to provide information. Any consequences are enforced at the state level.

6.3 Do individuals have the right to consent to particular uses of the information? If so, how does the individual exercise the right?

Yes. Information is collected only for specified circumstances or investigation, and this information is not utilized for any other purpose other than for those collected. Use of data is limited to the use for which it was collected and EMRS2 staff does not release information unless there is an overriding reason as stated under 5.1. Individuals do not access records in EMRS2. Freedom of Information Act requests must be in writing and should be submitted to the APHIS Privacy Act Officer, 4700 River Road Unit 50, Riverdale, MD 20737; or by facsimile (301) 734-5941; or by email APHISPrivacy@usda.gov. In accordance with 7 CFR 1.112 (Procedures for requests pertaining to individual records in a record system), the request must include the full name of the individual making the request; the name of the system of records; and preference of inspection, in person or by mail. In accordance with 7 CFR 1.113, prior to inspection of the records, the requester shall present sufficient identification to establish that the requester is the individual to whom the records pertain. In addition, if an individual submitting a request for access wishes to be supplied with copies of the records by mail, the requester must include with his or her request sufficient data for the agency to verify the requester's identity.

6.4 <u>Privacy Impact Analysis</u>: Describe how notice is provided to individuals, and how the risks associated with individuals being unaware of the collection are mitigated.

Notice is provided via the publicly available System of Record Notice, the Privacy Impact Assessment (this document) and Memorandum of Understanding with other organizations. No information is collected without the awareness of an individual. Permission is requested of the premise or animal owner to collect information. In the case of a disease outbreak, the federal government has jurisdiction/authority to collect animal health information relevant to an infected premise or premise in a control area. The Privacy Act Notice is posted on the EMRS2 Home Page as a point of reference and additional notification to the individuals.

Section 7.0 Access, Redress and Correction

The following questions are directed at an individual's ability to ensure the accuracy of the information collected about them.

7.1 What are the procedures that allow individuals to gain access to their information?

Individuals do not access records in EMRS2. Freedom of Information Act requests must be in writing and should be submitted to the APHIS Privacy Act Officer, 4700 River Road Unit 50, Riverdale, MD 20737; or by facsimile (301) 734-5941; or by email APHISPrivacy@usda.gov. In accordance with 7 CFR 1.112 (Procedures for requests pertaining to individual records in a record system), the request must include

the full name of the individual making the request; the name of the system of records; and preference of inspection, in person or by mail. In accordance with 7 CFR 1.113, prior to inspection of the records, the requester shall present sufficient identification to establish that the requester is the individual to whom the records pertain. In addition, if an individual submitting a request for access wishes to be supplied with copies of the records by mail, the requester must include with his or her request sufficient data for the agency to verify the requester's identity.

7.2 What are the procedures for correcting inaccurate or erroneous information?

Individuals seeking to contest or amend records maintained in this system of records must direct their request to the address indicated above in the "RECORD ACCESS PROCEDURES" paragraph and must follow the procedures set forth in 7 CFR 1.116 (Request for correction or amendment to record). All requests must state clearly and concisely what record is being contested, the reasons for contesting it, and the proposed amendment to the record.

If an individual experiences a change in contact information, they may reach out to their state or federal point of contact and request a correction. The state or federal contact, may then correct the information in the EMRS2 or elevate the request directly to the VS Emergency Management Coordinator.

7.3 How are individuals notified of the procedures for correcting their information?

Individuals may be notified if a record in this system of records pertains to them when the individuals request information utilizing the same procedures as those identified in the "RECORD ACCESS PROCEDURES" paragraph above.

7.4 If no formal redress is provided, what alternatives are available to the individual?

N/A. The formal redress process is described under section 7.1 above.

7.5 <u>Privacy Impact Analysis</u>: Please discuss the privacy risks associated with the redress available to individuals and how those risks are mitigated.

No privacy risk has been identified.

Section 8.0 Technical Access and Security

The following questions are intended to describe technical safeguards and security measures.

8.1 What procedures are in place to determine which users may access the system and are they documented?

Access to EMRS2 is based on the need to conduct business within USDA and is approved by an authorized APHIS VS official. Criteria, procedures, and controls are documented. Access must be requested in writing and approved by the supervisor and an EMRS2 account manager.

8.2 Will Department contractors have access to the system?

No

8.3 Describe what privacy training is provided to users either generally or specifically relevant to the program or system?

All USDA APHIS VS employees are required to complete annual Information Security Awareness Training and sign Rules of Behavior. This general training allows organizational users with access to personally identifiable information to receive privacy-related training.

8.4 Has Certification & Accreditation been completed for the system or systems supporting the program?

Yes. The USDA APHIS VS EMRS2 received a renewed Authority to Operate (ATO) that expires on May 8, 2023.

8.5 What auditing measures and technical safeguards are in place to prevent misuse of data?

Some of the technical safeguards for EMRS 2.0 using Dynamics CRM is a security model that includes auditing, role-based views, field-level security, and division of security. This means changes to records are tracked. Even the audit history on individual record and/or audit history summary is also tightly controlled with separate security settings to protect the integrity of the log. The security model only provides users with access only to the appropriate levels of information based on their role(s). Furthermore, views and field-level are role-based as well; preventing users from seeing, accessing, and/or making changes to individual fields or records they do not have access to. Finally, access control is a combination of eAuthentication (user credential and authentication) and authorization (EMRS2 roles).

The EMRS2GO mobile Application uses 3 controls to protect downloaded data.

- Assures the hard drive has Bit Locker or a similar data encryption application or the app will shut down before a download of any data.
- After 30 days, if the reference data have not been uploaded to the EMRS system, EMRS2Go performs a full data upload regardless of the options the user has selected.

- After 60 days, if the reference data have not been uploaded, EMRS2Go deletes the local repository and performs a full data upload regardless of the option the user selected. If user no longer has access to EMRS, no data is downloaded after local data is deleted.

8.6 <u>Privacy Impact Analysis</u>: Given the sensitivity and scope of the information collected, as well as any information sharing conducted on the system, what privacy risks were identified and how do the security controls mitigate them?

As to technical safeguards:

- The EMRS2 is continuously monitored to ensure changes to the data is logged and reviewed. Auditing is performed to also alert the database administrators to privileged action taken against the database objects. The logs are correlated and saved at an Enterprise level to aid forensic investigation if the need arises.
- Access control technical measures are in place and operating to ensure only users with approval can access the data, and the concept of least privileged is enforced to ensure only the minimum access and privileges are granted to enable users to perform the job function. User access is audited on a continual basis.
- Operational technical safeguards to prevent data misuse begin with access control. EMRS2 employs TLS encryption to protect data during transmission and enforces multifactor authentication for user access. Password controls, procedures, responsibilities, and policies follow USDA departmental standards. APHIS employees must use LincPass to access their computer and the APHIS network, including the VPN. There is no action that can be performed within the EMRS2 without identification and authentication.
- At the USDA and APHIS Enterprise level, intrusion detection and intrusion prevention, firewalls and antivirus measures are employed on a continuous basis.

Section 9.0 Technology

The following questions are directed at critically analyzing the selection process for any technologies utilized by the system, including system hardware and other technology.

9.1 What type of project is the program or system?

Animal Health/Incident Response Management.

9.2 Does the project employ technology which may raise privacy concerns? If so please discuss their implementation.

This application does not employ technology which may raise privacy concerns.

Section 10.0 Third Party Websites/Applications

The following questions are directed at critically analyzing the privacy impact of using third party websites and/or applications.

10.1 Has the System Owner (SO) and/or Information Systems Security Program Manager (ISSPM) reviewed Office of Management and Budget (OMB) memorandums M-10-22 "Guidance for Online Use of Web Measurement and Customization Technology" and M-10-23 "Guidance for Agency Use of Third-Party Websites and Applications"?

Yes.

10.2 What is the specific purpose of the agency's use of 3rd party websites and/or applications?

EMRS2 uses Bing Maps for imagery only and utilizes no other Bing Map services. These maps are needed to assess the geographical location of infected premises and at-risk premises within the control area and/or surveillance zone. Field responders utilize geographical coordinates to conduct surveillance activities in the affected area.

10.3 What personally identifiable information (PII) will become available through the agency's use of 3rd party websites and/or applications.

EMRS2 does not receive any personally identifiable information from third party websites or applications.

10.4 How will the PII that becomes available through the agency's use of 3rd party websites and/or applications be used?

N/A

10.5 How will the PII that becomes available through the agency's use of 3rd party websites and/or applications be maintained and secured?

N/A

10.6 Is the PII that becomes available through the agency's use of 3rd party websites and/or applications purged periodically?

N/A

10.7 Who will have access to PII that becomes available through the agency's use of 3rd party websites and/or applications?

N/A

10.8 With whom will the PII that becomes available through the agency's use of 3rd party websites and/or applications be shared - either internally or externally?

N/A

10.9 Will the activities involving the PII that becomes available through the agency's use of 3rd party websites and/or applications require either the creation or modification of a system of records notice (SORN)?

N/A

10.10 Does the system use web measurement and customization technology?

EMRS2 does not use web measurement and customization technology.

10.11 Does the system allow users to either decline to opt-in or decide to opt-out of all uses of web measurement and customization technology?

N/A

10.12 <u>Privacy Impact Analysis</u>: Given the amount and type of PII that becomes available through the agency's use of 3rd party websites and/or applications, discuss the privacy risks identified and how they were mitigated.

N/A

Responsible Officials

JONATHAN ZACK Digitally signed by JONATHAN ZACK Date: 2023.03.31 15:34:19 -04'00'

Jonathan T. Zack
EMRS2 System Owner
United States Department of Agriculture

Approval Signature

Janelle J. Jordan
APHIS Privacy Act Officer
United States Department of Agriculture

Angela Cole
Chief Privacy Officer/Deputy Assistant Chief Information Security Officer
Marketing and Regulatory Programs
United States Department of Agriculture

Privacy Threshold Analysis
Emergency Management Response Services 2.0

Policy and Directives

- Version: 4.1
- Date: March 23, 2022
- Prepared for: Marketing and Regulatory Programs (MRP)

United States Department of Agriculture

DOCUMENT ADMINISTRATION

Document Revision and History

Revision	Date	Author and Title	Office	Comments
3.7	11/19/2019	Elinor Gallelli	APHIS MRP	Added EMRS2 Specific content to template
3.8	12/14/20 20	Elinor Gallelli	APHIS MRP	Annual Review of PII
3.9	02/11/2021	Elinor Gallelli	APHIS MRP	Annual Review of PII
4.0	05/05/2022	Elinor Gallelli	APHIS MRP	Annual Review of PII
4.1	3/23/2023	Josh Luterman	APHIS MRP	Annual Review/Update

DOCUMENT REVIEW

Reviewer	Title	Date	Update: Y/N	If systemic, please provide comments
Elinor Gallelli	ISSM	2/23/2023	Y	Added front matter before Appendix A
Josh Luterman	ISSM	5/31/2022	N	Assumed ISSO duties for system.

Table of Contents

INTRODUCTION 1
WHAT IS A PTA? 1
THE DIFFERENCE BETWEEN A PTA AND PIA 2
COMPLETING A PTA 2
PTA REVIEW PROCESS 2
APPENDIX A. PRIVACY THRESHOLD ANALYSIS 4
AGENCY RESPONSIBLE OFFICIALS 10
AGENCY APPROVAL SIGNATURE 10
APPENDIX B. ACRONYMS 11
APPENDIX C. DEFINITIONS: 12
APPENDIX D. NIST SP 800-53 REVISION 4 APPENDIX J 13

Introduction

The United States Department of Agriculture (USDA) is committed to preserve and enhance privacy protections for all individuals, to promote transparency of USDA operations, and to serve as a leader in the federal privacy community. The Privacy Threshold Analysis (PTA) is one step in fulfilling this commitment. The purpose of the PTA is to help program managers and system owners determine whether a Privacy Impact Assessment (PIA) is required under section 208 of the E-Government Act of 2002. A properly completed and reviewed PTA provides documentation that a system owner has assessed whether or not a full PIA is required. To appropriately protect the confidentiality of PII, organizations should use a risk-based approach, see the National Institute of Standards and Technology (NIST) Special Publication (SP) 800-122: *Guide to Protecting the Confidentiality of Personally Identifiable Information, (PII)*: http://csrc.nist.gov/publications/nistpubs/800-122/sp800-122.pdf

In anticipation of NIST SP 800-53 revision 4, July 2012 or later, this PTA template is being revised to compliment and incorporate these changes. See NIST SP 800-53 rev 4: http://nvlpubs.nist.gov/nistpubs/SpecialPublications/NIST.SP.800-53r4.pdf

Additional reference material can be found at USDA Privacy Council webpage:

The Privacy Council webpage

What is a PTA?

Privacy Threshold Assessments or PTAs are currently incorporated into the security assessment and authorization (A&A) process, formerly known as certification & accreditation (C&A) process. A&A is the process by which the Department assures its systems meet appropriate security and operating standards. Through the A&A process, the system owner completes the PTA and reviews it with the Agency Official for Privacy (AOP).

For all systems within USDA, a PTA must be conducted in order to determine if a full Privacy Impact Assessment (PIA) is necessary. The PTA and PIA are tools used to identify and qualify the extent of security measures needed to protect privacy and personally identifiable information (PII). Some information systems will not require a full PIA. Information owners or stewards can be aided in making the determination of whether a full PIA is required by work closely with the system owner or program manager to first conduct the PTA. For example, an agency may submit a PTA on a system that does not collect PII. The system will have an official PTA on file documenting the determination that a PIA is not required.

Agencies are required to review their privacy documentation, PTA, PIA, and System of Record Notice, (SORN), at a minimum, annually. Agencies are required to review their PIA(s) and SORN(s) posted on the department's webpage on a reoccurring basis and immediately notify Privacy Office of any discrepancies.

The department's PIA(s) and SORN(s) are posted on the following webpages:

PIA: https://www.usda.gov/home/privacy-policy/privacy-impact-assessments

SORN: https://www.usda.gov/home/privacy-policy/system-records-notices

The USDA Privacy Office can be contacted at privacy@usda.gov if there are any questions or concerns regarding this guidance

The Difference between a PTA and PIA

A PTA is not a PIA. A PTA simply helps determine whether or not a PIA needs to be completed. A PTA does not fulfill the requirements of the E-Government Act of 2002 which requires USDA to conduct a PIA before developing or procuring IT systems; or initiating projects that collect, maintain, or disseminate PII from or about members of the public, or initiating, consistent with the Paperwork Reduction Act, a new electronic collection of PII.

Completing a PTA

The USDA has developed a PTA template to aid the Information Owner in determining whether or not a PIA needs to be completed, and for Departmental consistency and ease of use. The template includes questions to determine whether or not a PIA is required. These questions also consist of NIST 800-53 rev 4 privacy controls. The template is available on the Risk Management Framework 2.0 site within the Privacy Documents folder. However, if a non-PDF version is needed, contact the USDA Privacy Office at privacy@usda.gov.

All PTAs completed after the effective date of this guidance must conform with the guidance contained herein and in the format provided in the template. All questions in the PTA template must be completed; please do not delete or modify sections of the template.

PTA Review Process

- As an initial step, the project manager or system owner should review the PTA with the Agency Official for Privacy (AOP) to ensure that the PTA was completed correctly and accurately.
- The agency then submits the completed PTA to the USDA Privacy Office via email at privacy@usda.gov. The USDA Privacy Office reviews the completed document regardless of whether it originated from a component or headquarters.
- If the USDA Privacy Office is in agreement with the submitted PTA, the next step would be to complete the PIA if needed. If there is any disagreement, the USDA Privacy Office will meet with the information owner/steward, project manager, system owner, and AOP, as necessary to review the PTA and make any appropriate changes.

The agency can provide supplemental information to support their position which may consist of screen shots, data base field lists, etc.

- The approved PTA should be submitted during the initiation phase of the security assessment and authorization process.

Appendix A. Privacy Threshold Analysis

Summary Information	
**Date**	March 23, 2023
**Name of Project**	Emergency Management Response Services 2.0
**Name of Component:**	Veterinary Services (VS)
**Name of Information Owner/Steward:**	Jonathan Zack
**Phone of Information Owner/Steward:**	301-851-3460
**Email of Information Owner/Steward:**	Jonathan.T.Zack@.usda.gov
**Name of Project Manager:**	Steven Schafer
**Phone for Project Manager:**	970-286-5196
**Email for Project Manager:**	steven.schafer@usda.gov
**Name of System Owner:**	Jonathan Zack
**Phone for System Owner:**	301-851-3460
**Email for System Owner:**	Jonathan.T.Zack@.usda.gov

1. Describe the project and its purpose:

The Emergency Management Response Services 2.0 (EMRS2) records information from all foreign animal disease investigations and provides incident management. EMRS2 is used by Veterinary Services (VS) to manage and investigate animal disease outbreaks in the United States. EMRS2 is a Web-based task management system accessed by approved users using Microsoft Internet Explorer and includes Geographic Information System (GIS) mapping support for easy visualization of work areas.

2. Status of Project:

☐ This is a new development effort.

☒ This is an existing project.

Date first developed: 29 January 2014

Date last updated: 30 March 2022

A summary of the changes in the last release, fairly typical of updates:

1) A subscriber and message type were created so EMRS could electronically receive permit requests. Permit request and the resulting permit (if approved) are needed by producers to move product during a Foreign Animal Disease (FAD) outbreak.

3. Is the system in Cyber Security Assessment and Management (CSAM) C&A web?

☐ Unknown. (Please explain, in question 12)

☐ No.

☒ Yes.

Please list the system name and system identification number (must be the same as the system name/number in CSAM C&A web): Emergency Management Response Services 2.0; CSAM ID # 1962

4. Is this system classified in CSAM as: (please select one)

☒ **Parent**

☐ **Child**

(Please attach a copy of the data flow diagram or database schema)

EMRS Data flow

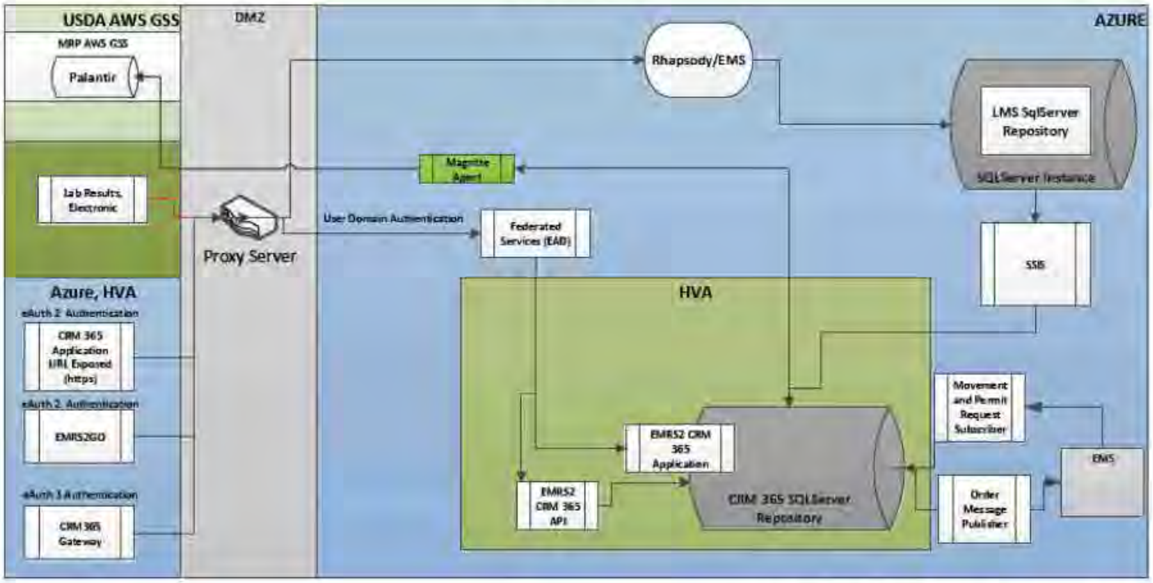

Notes:

1)Rhapsody receives electronic results either messaged by the lab or uploaded by spreadsheet loader(VS). Those messages are loaded to both Oracle and SQL Server repositories as shown. The results are imported to the EMRS2 CRM 365 from the SQLServer repository using SSIS.

2) EMRS2 CRM 365 is shown as a web application and a database, but this is an artificial separation: the application and underlying database are one- changes to the CRM database are made through the CRM application.

3) EMRS2GO uses eAuth 2 authentication against MSCRM 365 which then controls user authorization. All interaction of EMRS2GO is mediated through the MSCRM 365 API. Note it is a client desktop application not a web application.

4) Order Message Publisher uses existing account in EMRS with minimal privilege to pull only data needed and update a field to indicate it was published. Movement and Permit Request subscriber uses least privilege in retrieving messages and writing to appropriate EMRS entitie

Version	Document Date	Summary of Changes	Author
1.0	20 January 2016	Created	Todd Bleifuss
1.0	19 September 2016	Updated	Todd Bleifuss
1.1	9 November 2016	Updated	Todd Bleifuss
1.2	9 March 2017	Updated	Todd Bleifuss
1.3	7 August 2017	Updated	Todd Bleifuss
1.4	17 October 2017	CRM 365 branding	Bleifuss
1.5	23 Oct 2018	DIS	Bleifuss
1.5	14 Feb 2019	Azure Move	Bleifuss
1.6	2 Nov 2020	HVA Move	Bleifuss
1.7	27 July 2021	Added Publisher	Bleifuss
1.8	08 Sept 2021	Added EAD	E. Gallelli
1.9	27 Jan 2022	Added Mvmt sub	Bleifuss
2.0	17 Mar 2022	Added permit request sub	Bleifuss

5. Is this a cloud system?

☐ No.

☒ Yes.

6. Is this a contractor system?

☒ No.

☐ Yes.

If yes, to questions 5 or 6, please select appropriate box:

☐ Contractor (internal)

☒ Contractor (external)

☐ Federal providing contracted services

If any of the boxes are checked, please provide name of vendor and URL if applicable:__EMRS2 resides in the MRP Azure GSS_

7. **Does the system collect, process, generate or store PII information on: (Please check all that apply)**

☒ USDA employees.

☒ Contractors or other entities working on behalf of USDA.

☒ Non-USDA Federal Government employees.

☐ USDA Partner.

☒ The general public.

☒ Other.

If others, please list: State Agriculture Health Cooperators

8. **Does the system collect, process, generate or store any of the following information (that may be considered PII) on individuals: (Please check all that apply)**

☒ Name (full name).

☐ Date and/or place of birth.

☒ Address Information (street or email address).

☐ Personal identification number (e.g. social security number, tax identification number, passport number, driver's license number or a unique identification number, etc.)

☐ Financial data (credit card numbers, bank account numbers, etc.).

☐ Health data (including height, weight, blood pressure, etc.).

☐ Biometric data (fingerprints, iris scans, voice signature, facial geometry, DNA, etc.).

☐ Criminal history.

☐ Employment history.

☒ Miscellaneous identification numbers (Premise ID).

☐ Photographic image/identifying characteristics.

☐ Handwriting or an image of the signature.

☐ Other information that may be seen as personal (personal characteristics, etc.).

If so, please list:

9. Does the system use or collect Social Security Numbers (SSNs) or Tax Identification Numbers, (TINs)? (This includes truncated SSNs/TINs e.g. last 4 digits)?

☒ No.

☐ Yes.

If yes, why does the project collect SSNs or TINs? Provide the function of the SSN/TIN and the legal authority to do so:

9a. **Does the system utilize the following security controls?**

☒ Encryption.

☐ Masking of PII data.

☒ Controlled access.

☒ Timeout for remote access.

☒ System audit logs.

10. Does the system require the user to enter a user name and password in order to gain access to the system (e.g. e-Authentication)?

☐ No. (Please explain.)

☒ Yes.

If yes, please describe the authentication process:

Users accessing EMRS 2 or EMRS2GO must successfully authenticate using their e-Authentication PIV or e-authentication username/password credential and be authorized with specific EMRS role(s). Access control is a combination of eAuthentication Level 2 (user credential and authentication) and authorization (EMRS 2 roles).

11. Does the system connect, receive, or share PII[1] with any other USDA systems?

☐ No.

☒ Yes.

If yes, please list the other USDA systems:

Emergency Qualifications System (EQS) – view only, no direct interface; receives PII from Animal Disease Traceability Information System (ADTIS); sends PII to the Palantir component of the Marketing Regulatory Program General Support System – (MRP AWS GSS)

12. Does the system connect, receive, or share PII with any non-USDA systems?

☒ No.

[1] Personally Identifiable Information (PII) is information that can identify a person. This may include: name, address, phone number, social security number, image, as well as health information or a physical description.

☐ Yes.

If yes, please list the non-USDA systems:

13. Matching records via computer/automated process, performed by federal agency, whether the personal records used in the match are federal or non-federal PII. Reference DR 3450-001: http://www.ocio.usda.gov/directives/doc/DR3450-001.pdf

a. Are you comparing two or more PII records or system of records?

☒ No.

☐ Yes

b. Are you comparing any system of record with non -federal records?

☒ No.

☐ Yes

If yes, for question 13a or 13b, the efforts or purpose have to meet at least one of these conditions:

- Creating or checking eligibility or compliance with laws/regulations of applicants or recipients/beneficiaries of a federal program/grant.

  OR

- Recouping payments, delinquent debts or overpayments owed to government agencies from a federal benefit program.
  OR

- Two or more automated Federal personnel or payroll systems of records or a system of Federal Personnel of payroll records with non-federal records.

Exclusions for the conditions above: *Aggregate statistical, research or statistical project, enforcement of criminal laws, tax information, etc. Please see PL 100-503, Computer Matching Act for specific details.*

c. Based on the responses above, is a CMA required?

(Affirmative for 13a. or 13b and either of the options for efforts are met),

☒ No. (Skip to question 15)

☐ Yes. (Please respond to question 14, if "yes").

14. Do you have a Computer Matching Agreement?

☐ No.

☐ Yes. (Please list this agreement on the Privacy Council webpage posting)

15. Are there regular (e.g. periodic, recurring, etc.) PII data extractions from the system?

☐ No.

☒ Yes.

(Reference Memorandum – posted on website)

If yes, have proper controls and policies been developed to address the data logging requirements outlined in Office of Management and Budget (OMB) Memorandum
M-07-16, "*Safeguarding Against and Responding to the Breach of Personally Identifiable Information*"?

☐ No.

☒ Yes.

16. Does the system track or measure the browsing habits or preferences of the public or user? (refer to OMB Memoranda M-10-22 "*Guidance for Online Use of Web Measurement and Customization Technologies*" and M-10-23 "*Guidance for Agency Use of Third-Party Websites and Applications*")

☒ No.

☐ Yes.

If yes, have proper controls and policies been developed to meet all the requirements outlined in Office of Management and Budget (OMB) Memoranda M-10-22 "*Guidance for Online Use of Web Measurement and Customization Technologies*" and M-10-23 "*Guidance for Agency Use of Third-Party Websites and Applications.*"

☐ No.

☐ Yes

17. Is application/system mobile device compatible? (Y/N)

☐ No.

☒ Yes

If none of the boxes were checked for questions number 7 – 8 and **"NO"** was answered for questions 9, 11, and 12, **DO NOT** complete a PIA for this system.

If any box was checked for questions number 7 - 8, and any answers to questions 9 through 12 were "**YES**," A PIA **MUST** be completed for this system.

**PIA REQUIRED**	
**YES**	**NO**
X	

(Check one)

Privacy Office reserves the right to request additional information during the review of privacy documentation for the systems.

Signature authority and protocol differs by agency, we request at a minimum Project Manager/System Owner and ISSPM/CISO sign the document with review by the Privacy Officer.

Agency Responsible Officials

JONATHAN ZACK Digitally signed by JONATHAN ZACK Date: 2023.03.31 15:35:37 -04'00'

Jonathan T. Zack, DVM
EMRS2 System Owner
United States Department of Agriculture

Date

Agency Approval Signature

Tonya Woods
APHIS Privacy Officer
United States Department of Agriculture

Date

Angela Cole
Chief Privacy Officer/Deputy Assistant Chief Information Security Officer
Marketing and Regulatory Programs
United States Department of Agriculture

Date

Appendix B. Acronyms

Acronyms used in this document are listed below in alphabetical order.

Acronym	Description
A&A	Assessment and Authorization (formerly Certification & Accreditation)
AOP	Agency Official for Privacy
CMA	Computer Matching Agreement
CIO	Chief Information Officer
CISO	Chief Information Security Officer
CPO	Chief Privacy Officer
CSAM	Cyber Security Assessment and Management
EOM	End of Month
NIST	National Institute of Standards and Technology
OMB	Office of Management and Budget
PIA	Privacy Impact Assessment
PII	Personal Identifiable Information
PTA	Privacy Threshold Analysis
SAOP	Senior Agency Official for Privacy
SORN	System of Record Notice
SP	Special Publication
SSN	Social Security Number
SSP	System Security Plan
TIN	Tax Identification Number
USDA	United States Department of Agriculture (often referred as "Department")

Appendix C. DEFINITIONS:

Term	Definition
Computer Matching Agreement, CMA	The Computer Matching and Privacy Protection Act covers two kinds of matching programs: (1) matches involving Federal benefits programs; and (2) matches using automated records from Federal personnel or payroll systems of records.
Generate	Generate is defined as the creation of an item. For the purpose of privacy documentation, generate in terms of the system creating PII data.
Process	Process is defined as a method or action that results in a transformation or alteration of data. For the purpose of privacy documentation, system manipulate or change the PII data within the system.
Third party websites/applications	The term "third-party websites or applications" refers to web-based technologies that are not exclusively operated or controlled by a government entity, or web-based technologies that involve significant participation of a nongovernment entity. Often these technologies are located on a ".com" website or other location that is not part of an official government domain. However, third-party applications can also be embedded or incorporated on an agency's official website.
Store	Store is defined as a location in which data is retained. For the purpose of privacy documentation, system contain or maintain for future access PII data.

Appendix D. NIST SP 800-53 Revision 4 Appendix J

Privacy controls are the administrative, technical, and physical safeguards employed within organizations to protect and ensure the proper handling of PII. There are eight privacy control families with each family aligning with one of the Federal Information Processing Standards (FIPS.) The privacy control families can be implemented at the organization, department, agency, component, office, program, or information system level, under the leadership of the Senior Agency Official for Privacy (SAOP) or Chief Privacy Officer (CPO)[2] and in coordination with the Chief Information Security Officer (CISO), Chief Information Officer (CIO), program officials, and legal counsel. Table J-1 provides a summary of the privacy controls by family in the privacy control catalog

TABLE J-1: SUMMARY OF PRIVACY CONTROLS BY FAMILY

CNTL NO.	PRIVACY CONTROLS
**AP**	**Authority and Purpose**
AP-1	Authority to Collect
AP-2	Purpose Specification
**AR**	**Accountability, Audit, and Risk Management**
AR-1	Governance and Privacy Program
AR-2	Privacy Impact and Risk Assessment
AR-3	Privacy Requirements for Contractors and Service Providers
AR-4	Privacy Monitoring and Auditing
AR-5	Privacy Awareness and Training
AR-6	Privacy Reporting
AR-7	Privacy-Enhanced System Design and Development
AR-8	Accounting of Disclosures
**DI**	**Data Quality and Integrity**
DI-1	Data Quality
DI-2	Data Integrity and Data Integrity Board
**DM**	**Data Minimization and Retention**
DM-1	Minimization of Personally Identifiable Information
DM-2	Data Retention and Disposal
DM-3	Minimization of PII Used in Testing, Training, and Research
**IP**	**Individual Participation and Redress**
IP-1	Consent
IP-2	Individual Access
IP-3	Redress
IP-4	Complaint Management
**SE**	**Security**
SE-1	Inventory of Personally Identifiable Information

[2] All federal agencies and departments designate an *SAOP/CPO* as the senior organizational official with the overall organization-wide responsibility for information privacy issues. OMB Memorandum 05-08, provides guidance for the designation of SAOPs/CPOs.

CNTL NO.	PRIVACY CONTROLS
SE-2	Privacy Incident Response
**TR**	**Transparency**
TR-1	Privacy Notice
TR-2	System of Records Notices and Privacy Act Statements
TR-3	Dissemination of Privacy Program Information
**UL**	**Use Limitation**
UL-1	Internal Use
UL-2	Information Sharing with Third Parties

Source:
NIST Special Publication 800-53-Rev.4, *Security and Privacy Controls for Federal Information Systems and Organizations*

Notices

Federal Register
Vol. 86, No. 234
Thursday, December 9, 2021

This section of the FEDERAL REGISTER contains documents other than rules or proposed rules that are applicable to the public. Notices of hearings and investigations, committee meetings, agency decisions and rulings, delegations of authority, filing of petitions and applications and agency statements of organization and functions are examples of documents appearing in this section.

DEPARTMENT OF AGRICULTURE

Submission for OMB Review; Comment Request

December 6, 2021.

The Department of Agriculture will submit the following information collection requirement(s) to OMB for review and clearance under the Paperwork Reduction Act of 1995, Public Law 104–13 on or after the date of publication of this notice. Comments are requested regarding: (1) Whether the collection of information is necessary for the proper performance of the functions of the agency, including whether the information will have practical utility; (2) the accuracy of the agency's estimate of burden including the validity of the methodology and assumptions used; (3) ways to enhance the quality, utility and clarity of the information to be collected; and (4) ways to minimize the burden of the collection of information on those who are to respond, including through the use of appropriate automated, electronic, mechanical, or other technological collection techniques or other forms of information technology.

Comments regarding these information collections are best assured of having their full effect if received by January 10, 2022. Written comments and recommendations for the proposed information collection should be submitted within 30 days of the publication of this notice on the following website *www.reginfo.gov/public/do/PRAMain*. Find this particular information collection by selecting "Currently under 30-day Review—Open for Public Comments" or by using the search function.

An agency may not conduct or sponsor a collection of information unless the collection of information displays a currently valid OMB control number and the agency informs potential persons who are to respond to the collection of information that such persons are not required to respond to the collection of information unless it displays a currently valid OMB control number.

National Agricultural Statistics Service (NASS)

Title: Mink Survey.

OMB Control Number: 0535–0212.

Summary of Collection: The primary objective of the National Agricultural Statistics Service is to prepare and issue State and national estimates of crop and livestock production, prices, and disposition. The Mink Survey collects data on the number of mink pelts produced, the number of females bred, the value of pelts produced, and the number of mink farms. Mink estimates are used by the federal government to calculate total value of sales and total cash receipts, by State governments to administer fur farm programs and health regulations, and by universities in research projects.

Need and Use of the Information: NASS collects information on mink pelts produced by color, number of females bred to produce kits the following year, number of mink farms, average marketing price, and the value of pelts produced. The data is disseminated by NASS in the Mink Report and is used by the U.S. government and other groups.

Description of Respondents: Farms.

Number of Respondents: 253.

Frequency of Responses: Reporting: Annually.

Total Burden Hours: 87.

National Agricultural Statistics Service

Title: Cost of Pollination Survey.

OMB Control Number: 0535–0258.

Summary of Collection: The primary objective of the National Agricultural Statistics Service (NASS) is to prepare and issue state and national estimates of crop and livestock production, prices, and disposition; as Start Printed Page 53270 well as economic statistics, environmental statistics related to agriculture, and to conduct the Census of Agriculture. Pollinators (honeybees, bats, butterflies, hummingbirds, etc.) are vital to the agricultural industry for pollinating numerous food crops for the world's population. Concern for honeybee colony mortality has risen since the introduction of *Varroa* mites in the United States in the late 1980s and the appearance of Colony Collapse Disorder in the past decade.

These data will be collected under the authority of 7 U.S.C. 2204(a). Individually identifiable data collected under this authority are governed by Section 1770 of the Food Security Act of 1985 as amended, 7 U.S.C. 2276, which requires USDA to afford strict confidentiality to non-aggregated data provided by respondents. This Notice is submitted in accordance with the Paperwork Reduction Act of 1995 (Pub. L. 104–113) and the Office of Management and Budget regulations at 5 CFR part 1320. This survey is also conducted in accordance with the Confidential Information Protection and Statistical Efficiency Act of 2018, Title III of Public Law 115–435, codified in 44 U.S.C. Ch. 35.

Need and Use of the Information: NASS will collect economic data from crop farmers who rely on pollinators for their crops (fruits, nuts, vegetables, etc.). Data relating to the targeted crops are collected for the total number of acres that rely on honeybee pollination, the number of honeybee colonies that were used on those acres, and any cash fees associated with honeybee pollination. Crop Farmers are also asked if beekeepers who were hired to bring their bees to their farm were notified of pesticides used on the target acres, how many acres they were being hired to pollinate, and how much they were being paid to pollinate the targeted crops.

Description of Respondents: Farmers.

Number of Respondents: 18,000.

Frequency of Responses: Reporting: Once a year.

Total Burden Hours: 5,454.

Levi S. Harrell,

Departmental Information Collection Clearance Officer.

[FR Doc. 2021–26690 Filed 12–8–21; 8:45 am]

BILLING CODE 3410–20–P

DEPARTMENT OF AGRICULTURE

Animal and Plant Health Inspection Service

[Docket No. APHIS–2020–0015]

Privacy Act of 1974; System of Records

AGENCY: Animal and Plant Health Inspection Service, USDA.

ACTION: Notice of a modified system of records.

SUMMARY: Pursuant to the Privacy Act of 1974 and Office of Management and Budget Circular No. A–108, the U.S. Department of Agriculture (USDA) give notice that a component agency, the Animal and Plant Health Inspection Service (APHIS) proposes to modify an existing system of records notice titled Emergency Management Response System (EMRS), USDA/APHIS–11. This system, among other things, helps APHIS to manage and investigate incidents of foreign animal diseases within the United States.

DATES: In accordance with 5 U.S.C. 552a(e)(4) and (11), this notice is applicable upon publication, subject to a 30-day notice and comment period in which to comment on the routine uses described in the routine uses section of this system of records notice. Please submit any comments by January 10, 2022.

ADDRESSES: You may submit comments by either of the following methods:

• *Federal eRulemaking Portal:* Go to *http://www.regulations.gov.* Enter APHIS–2020–0015 in the Search field. Select the Documents tab, then select the comment button in the list of documents.

• *Postal Mail/Commercial Delivery:* Send your comment to Docket No. APHIS–2020–0015, Regulatory Analysis and Development, PPD, APHIS, Station 3A–03.8, 4700 River Road, Unit 118, Riverdale, MD 20737–1238.

Supporting documents and any comments we receive on this docket may be viewed at *http://www.regulations.gov* or in our reading room, which is located in Room 1620 of the USDA South Building, 14th Street and Independence Avenue SW, Washington, DC. Normal reading room hours are 8 a.m. to 4:30 p.m., Monday through Friday, except holidays. To be sure someone is there to help you, please call (202) 799–7039 before coming.

FOR FURTHER INFORMATION CONTACT: For general questions, please contact Dr. Fred G. Bourgeois, EMRS National Coordinator, Strategy and Policy, National Preparedness and Incident Command, VS, APHIS, Lake Charles, LA; (318) 288–4083; *fred.g.bourgeois@usda.gov.* For Privacy Act questions concerning this system of records notice, please contact Ms. Tonya Woods, Director, Freedom of Information and Privacy Act Staff, 4700 River Road, Unit 50, Riverdale, MD 20737; (301) 851–4076. For USDA Privacy Act questions, please contact the USDA Chief Privacy Officer, Information Security Center, Office of Chief Information Officer, USDA, Jamie L. Whitten Building, 1400 Independence Ave. SW, Washington, DC 20250; email: *USDAPrivacy@usda.gov.*

SUPPLEMENTARY INFORMATION: The U.S. Department of Agriculture (USDA) Animal and Plant Health Inspection Service (APHIS) is modifying an existing system of records notice for APHIS' Emergency Management Response System (EMRS), USDA/APHIS–11, which was last published on April 30, 2008, in its entirety in the **Federal Register** (73 FR 23409–23412, Docket No. APHIS–2008–0039).[1]

EMRS is used by APHIS' Veterinary Services (VS) to help manage, coordinate, report, and investigate activities such as incidents of foreign animal diseases in the United States (including disposal, cleaning and disinfection, and associated indemnity payments), surveillance and control programs, State-specific disease outbreaks, national animal health emergency responses (all-hazards), and allow for tracing of animal movement and records, as well as premises and activity mapping. If an animal disease were to be detected in the United States, VS would activate its Incident Command System (ICS). ICS team members are trained to control and eradicate foreign animal diseases. As necessary and appropriate for the specific incident, team members would, among other things, confirm the presence of the disease, inspect infected and exposed animals, appraise the value of animals that may have to be destroyed, conduct vaccination programs and epidemiological studies, dispose of animal carcasses, and clean and disinfect premises. Records of these activities would be maintained in EMRS.

APHIS is making the following changes to the system of records notice:

• Updating the system location and system manager;

• Updating the purpose of the system;

• Expanding the categories of individuals to identify the roles of the APHIS employees included in the system and to add responders and coordinators since these individuals will participate in activities associated with the system;

• Making minor editorial changes to the categories of records;

• Revising the record source categories to add reference to a database within EMRS and to add that information in the system may be obtained from the Financial Modernization Incentive for payment status;

• Updating the policies and practices for storage, retrievability, and retention and disposal of records in the system;

• Updating the system safeguards;

• Updating the notification, record access, and contesting record procedures; and

• Deleting, revising, redesignating, and establishing routine uses as follows:

○ Revising current routine uses 1 and 2 to add reference to Tribal animal health officials and, in routine use 1, adding that information may be shared to identify premises before an event to allow for faster response;

○ Deleting current routine use 3 because EMRS has never shared data or connected data to/from the Department of Homeland Security's (DHS) National Biosurveillance Integration System (now known as Biosurveillance Common Operating Network (BCON)) and APHIS' Offshore Pest Information System (OPIS). However, if this should change, information would be shared with DHS' BCON system as described in routine uses 1 and 2. A routine use for OPIS is not needed since it is a system that is internal to USDA;

○ Revising current routine use 4 and redesignating it as routine use 3. The changes are editorial and intended to more accurately describe the referral of records to appropriate law enforcement agencies, entities, and persons;

○ Revising current routine use 5 and redesignating it as routine use 4. The changes are editorial and conforming changes;

○ Revising current routine use 6 and redesignating it as routine use 5. The changes are editorial and intended to more accurately describe the disclosure of records to a court or adjudicative body;

○ Revising current routine use 7 and redesignating as routine use 6. The changes are editorial and intended to more accurately describe the disclosure of records to appropriate agencies;

○ Establishing new routine use 7 for disclosure to another Federal agency or entity of information reasonably necessary to assist in responding to a suspected or confirmed breach or to prevent, minimize, or remedy harm, in accordance with Office of Management and Budget (OMB) Memorandum M–17–12 (Preparing for and Responding to a Breach of Personally Identifiable Information);

○ Revising current routine use 8. The changes are editorial and intended to more accurately describe disclosure to USDA contractors and other parties assisting in administering the program, analyzing data, information

[1] To view the notice, go to *www.regulations.gov* and enter APHIS–2008–0039 in the Search field.

management systems, Freedom of Information Act requests, and audits;

○ Removing current routine use 9 since this routine use is included in revised routine use 8;

○ Establishing new routine use 9 to describe disclosure to Congressional offices in response to an inquiry made at the written request of the individual to whom the record pertains; and

○ Revising current routine use 10 to more accurately reflect where record management inspections may occur.

A report on the modified system of records, required by 5 U.S.C. 552a(r), as implemented by OMB Circular A–108, was sent to the Chairman, Committee on Homeland Security and Governmental Affairs, United States Senate; the Chairwoman, Committee on Oversight and Reform, House of Representatives; and the Administrator, Office of Information and Regulatory Affairs, OMB.

Done in Washington, DC, this 30th day of November 2021.

Jack Shere,

Acting Administrator, Animal and Plant Health Inspection Service.

SYSTEM NAME AND NUMBER:

USDA/APHIS–11, Emergency Management Response System (EMRS).

SECURITY CLASSIFICATION:

None.

SYSTEM LOCATIONS:

The Animal and Plant Health Inspection Service (APHIS) located at 4700 River Road, Riverdale, MD 20737, is responsible for the system. EMRS records are maintained in a Government-approved cloud server accessed through secure data centers in the continental United States. Paper files are held at various Veterinary Services (VS) national, district, and field offices. Due to the number of offices, specific addresses can be found at: *https://www.aphis.usda.gov/aphis/ourfocus/animalhealth/contact-us.* Cloud service providers are MS Azure Government (US Gov Virginia), 101 Herbert Dr., Boydton, VA 23917 (Eastern Region); and MS Azure Government (US Gov Texas), 5150 Rogers Road, San Antonio, TX 78251 (Western Region).

SYSTEM MANAGER:

EMRS National Coordinator, National Preparedness & Incident Coordination, Veterinary Services, APHIS, USDA, Lake Charles, LA; (318) 288–4083.

AUTHORITY FOR MAINTENANCE OF THE SYSTEM:

Animal Health Protection Act (7 U.S.C. 8301 *et seq.*).

PURPOSES OF THE SYSTEM:

APHIS' VS program uses EMRS to help manage, coordinate, report, and investigate activities such as incidents of foreign animal diseases in the United States (including disposal, cleaning and disinfection, and associated indemnity payments), surveillance and control programs, State-specific disease outbreaks, national animal health emergency responses (all-hazards), and allow for tracing of animal movement and records, as well as premises and activity mapping. To fulfill this purpose, EMRS allows for APHIS to use visualization software to build premises maps and epidemiological models. EMRS will also maintain information concerning APHIS employees who may be deployed as members of Incident Command System teams.

CATEGORIES OF INDIVIDUALS COVERED BY THE SYSTEM:

Categories of individuals covered by the system include, but are not limited to, customers, such as State animal health officials and industry, who obtain services under EMRS, including the owner or operator of the premises where the animals subject to investigation are located and the referring contact who provided initial premises information; APHIS employees involved in the diagnostic and investigation activities; and responders and cooperators.

CATEGORIES OF RECORDS IN THE SYSTEM:

Categories of records in the system include:

Owner or operator of the premises where the animals subject to investigation are located; the system includes the following information, such as, but not limited to, the name; address (including city, county, State, postal code, and latitude/longitude coordinates); premises identification number; and telephone number.

Referring contact information, which includes name and telephone number.

Case coordinator of the premises investigation. (The system includes name, telephone number, and email address.)

APHIS employees. (The system includes information such as, but not limited to, the name; agency, program, and group; current duty assignment; encrypted employee identification number; grade, series, and step; duty city and State; home address, including latitude/longitude coordinates; home telephone number; home email address; emergency contact information; work and field addresses, email addresses and telephone numbers; supervisor contact information; personal protective equipment type, size, and model; existing and desired skills, experience and training; position certifications; AgLearn training classes; medical clearance information; and a description of property or fleet vehicle assigned to the employee.)

The system will also include nicknames, titles, and organization for the entities above, as applicable.

RECORD SOURCE CATEGORIES:

Information in this system comes primarily from the customers, including the owner or operator of the premises where the animals subject to investigation are located, the referring contact who provided initial premises information, and case coordinator. Such information may be supplemented by information from an address-validation database, by APHIS personnel during an on-site investigation, by State and Tribal veterinary offices and State laboratories, or by APHIS' National Veterinary Services Laboratories. Information may also be obtained from the Financial Management Modernization Incentive for payment status. Employee information is obtained primarily from the employee. Additionally, employee information may be obtained from the U.S. Department of Agriculture's (USDA's) National Finance Center, AgLearn database, and Federal Occupational Health, U.S. Department of Health and Human Services.

ROUTINE USES OF RECORDS MAINTAINED IN THE SYSTEM, INCLUDING CATEGORIES OF USERS AND PURPOSES OF SUCH USES:

In addition to those disclosures generally permitted under 5 U.S.C. 552a(b) of the Privacy Act, records contained in the system may be disclosed outside USDA as a routine use under 5.U.S.C. 552a(b)(3), to the extent that such uses are compatible with the purposes for which the information was collected. Such permitted routine uses include the following:

(1) To certain Federal, State, and Tribal animal health officials to identify premises before an event to allow for faster response, monitor the status of an animal disease investigation, document actions taken relating to an animal disease investigation, track the status of animals susceptible to foreign animal diseases, determine the costs of an animal disease investigation, monitor the use and availability of assets and personnel relating to animal disease investigations, or perform epidemiological and geospatial analyses of such investigations;

(2) To Federal, State, and Tribal animal health officials within the system to obtain feedback regarding the

EMRS system and emergency preparedness guidelines, and to educate and involve them in program development, program requirements, and standards of conduct;

(3) When a record on its face, or in conjunction with other records, indicates a violation or potential violation of law, whether civil, criminal, or regulatory in nature, and whether arising by general statute or particular program, statute, or by regulation, rule, or order issued pursuant thereto, disclosure may be made to the appropriate agency, whether Federal, foreign, State, Tribal, local, or other public authority responsible for enforcing, investigating, or prosecuting such violation or charged with enforcing or implementing the statute, or rule, regulation, or order issued pursuant thereto, if the information disclosed is relevant to any enforcement, regulatory, investigative, or prosecutive responsibility of the receiving entity;

(4) To the Department of Justice when: (a) USDA or any component thereof; or (b) any employee of USDA in his or her official capacity, where the Department of Justice has agreed to represent the employee; or (c) the United States Government, is a party to litigation or has an interest in such litigation, and USDA determines that the records are both relevant and necessary to the litigation and the use of such records by the Department of Justice is for a purpose that is compatible with the purpose for which USDA collected the records;

(5) In an appropriate proceeding before a court, grand jury, or administrative or adjudicative body or official, when USDA or other Agency representing USDA determines that the records are relevant and necessary to the proceeding; or in an appropriate proceeding before an administrative or adjudicative body when the adjudicator determines the records to be relevant to the proceeding;

(6) To appropriate agencies, entities, and persons when: (a) USDA suspects or has confirmed that the security or confidentiality of information in the system of records has been compromised; (b) USDA has determined that as a result of the suspected or confirmed breach there is a risk of harm to individuals, USDA (including its information systems, programs, and operations), the Federal Government, or national security; and (c) the disclosure made to such agencies, entities, and persons is reasonably necessary to assist in connection with USDA's efforts to respond to the suspected or confirmed compromise and prevent, minimize, or remedy such harm;

(7) To another Federal agency or Federal entity, when information from this system of records is reasonably necessary to assist the recipient agency or entity in (a) responding to a suspected or confirmed breach or (b) preventing, minimizing, or remedying the risk of harm to individuals, the agency (including its information systems, programs, and operations), the Federal Government, or national security;

(8) To contractors and their agents, grantees, experts, consultants, and others performing or working on a contract, service, grant, cooperative agreement, or other assignment for the USDA, when necessary to accomplish an agency function related to this system of records;

(9) To a Congressional office in response to an inquiry from that Congressional office made at the written request of the individual about whom the record pertains; and

(10) To the National Archives and Records Administration (NARA) or other Federal Government agencies pursuant to records management inspections being conducted under 44 U.S.C. 2904 and 2906.

DISCLOSURE TO CONSUMER REPORTING AGENCIES:

None.

POLICIES AND PRACTICES FOR STORAGE OF RECORDS:

Electronic records are stored on servers located as indicated above under "System Locations". Paper files are held at various VS national, district, and field offices that are locked during non-business hours and require presentation of employee identification for admittance and access at all times.

POLICIES AND PRACTICES FOR RETRIEVAL OF RECORDS:

Data can be retrieved only by personnel who successfully authenticate using their eAuthentication PIV or eAuthentication username/password credential and are authorized with specific EMRS role(s). Data can be retrieved by premises identification number, reference control number, name, premises, incident group, or incident site. Data regarding an employee, cooperator, or responder can be retrieved by name, nickname, employee identification number, title, organization, property, or fleet vehicle.

POLICIES AND PRACTICES FOR RETENTION AND DISPOSAL OF RECORDS:

In accordance with NARA-approved records disposition authorities, paper records will be retained for the following periods of time: All incident-related premise record data associated with a foreign animal disease investigation will be retained for a period of 50 years. For the remaining records, APHIS is in the process of preparing a records disposition request from NARA, and these records will be retained until appropriate disposition authority is obtained from NARA.

ADMINISTRATIVE, TECHNICAL, AND PHYSICAL SAFEGUARDS:

The EMRS safeguards include management, operational, and technical controls to prevent misuse of data by system users. These controls include role-based access. State and Tribal entities have access limited to data from their State or area. Access to the restricted portions of the database system requires certain levels of authorization through USDA eAuthentication, which is a system that enables individuals to obtain user-identification accounts with password-protected access to certain USDA web-based applications and services through the internet. APHIS personnel who input data must have a high-level eAuthentication account.

RECORD ACCESS PROCEDURES:

All requests for access to records must be in writing and should be submitted to the APHIS Privacy Act Officer, 4700 River Road, Unit 50, Riverdale, MD 20737; or by facsimile (301) 734–5941; or by email *APHISPrivacy@usda.gov*. In accordance with 7 CFR 1.112 (Procedures for requests pertaining to individual records in a record system), the request must include the full name of the individual making the request; the name of the system of records; and preference of inspection, in person or by mail. In accordance with 7 CFR 1.113, prior to inspection of the records, the requester shall present sufficient identification (*e.g.*, driver's license, employee identification card, social security card, credit cards) to establish that the requester is the individual to whom the records pertain. In addition, if an individual submitting a request for access wishes to be supplied with copies of the records by mail, the requester must include with his or her request sufficient data for the agency to verify the requester's identity.

CONTESTING RECORD PROCEDURES:

Individuals seeking to contest or amend records maintained in this system of records must direct their request to the address indicated above in the "RECORD ACCESS PROCEDURES" paragraph and must follow the procedures set forth in 7 CFR 1.116 (Request for correction or

amendment to record). All requests must state clearly and concisely what record is being contested, the reasons for contesting it, and the proposed amendment to the record.

NOTIFICATION PROCEDURES:

Individuals may be notified if a record in this system of records pertains to them when the individuals request information utilizing the same procedures as those identified in the ''RECORD ACCESS PROCEDURES'' paragraph above.

EXEMPTIONS PROMULGATED FOR THE SYSTEM:

None.

HISTORY:

On April 30, 2008 (73 FR 23409–23412, Docket No. APHIS–2008–0039), USDA/APHIS–11, ''Emergency Management Response System'' was published as a new system of records and effective on June 9, 2008.

[FR Doc. 2021–26684 Filed 12–8–21; 8:45 am]

BILLING CODE 3410–34–P

DEPARTMENT OF AGRICULTURE

Rural Business-Cooperative Service

[Docket #: RBS–21–Business–0036]

Notice of Funding Opportunity for the Food Supply Chain Guaranteed Loan Program

AGENCY: Rural Business—Cooperative Service, USDA.

ACTION: Notice.

SUMMARY: The Rural Business—Cooperative Service (Agency), an agency of the United States Department of Agriculture (USDA) Rural Development mission area (RD) announces the availability of approximately $1,000,000,000 in loan guarantees, applicant and application requirements, and servicing requirements under the Food Supply Chain (FSC) Guaranteed Loan Program for fiscal year (FY) 2022. Loan guarantees will be made to lenders to facilitate financing to qualified borrowers and projects for the start-up or expansion of activities in the middle of the food supply chain, particularly the aggregation, processing, manufacturing, storage, transportation, wholesaling, or distribution of food, to increase capacity and help create a more resilient, diverse, and secure U.S. food supply chain.

DATES: Completed applications may be submitted beginning December 9, 2021. Awards will be made no earlier than February 7, 2022. Applications will be accepted until funds are exhausted.

ADDRESSES: You are encouraged to contact the Agency to discuss your project and ask any questions about the program or application process. Applications will only be accepted electronically by following the directions provided at *https://www.rd.usda.gov/foodsupplychainloans.*

Entities wishing to apply for assistance may download the application documents and requirements delineated in this notice from: *https://www.rd.usda.gov/foodsupplychainloans.*

FOR FURTHER INFORMATION CONTACT: Jeff Hudson, Rural Business—Cooperative Service, United States Department of Agriculture, 1400 Independence Avenue SW, Mail Stop 3201, Room 5801—South, Washington, DC 20250–3201; *rdfoodsupplychainloans@usda.gov,* or phone 715–345–7636.

SUPPLEMENTARY INFORMATION: All applicants are responsible for any expenses incurred in developing their applications.

The lender is responsible for assuring that all requirements for making, securing, servicing, and collecting the loan have been met.

Whether specifically stated or not, whenever Agency approval is required, it must be in writing. Copies of all forms and regulations referenced in this notice may be obtained from any Agency office and from the USDA RD website at *https://www.rd.usda.gov/foodsupplychainloans.*

Overview

Federal Agency Name: Rural Business—Cooperative Service.

Funding Opportunity Title: Food Supply Chain Guarantee Loan Program.

Announcement Type: Initial Notice.

Assistance Listing Number: 10.380.

Dates: Applications will be accepted beginning December 9, 2021. Application acceptance will continue until all funds are expended.

Administrative: Applicants are encouraged to consider projects that will advance the following key priorities (additional information on the key priorities is available at *https://www.rd.usda.gov/priority-points*):

- Assisting rural communities recover economically from the impacts of the COVID–19 pandemic, particularly disadvantaged communities;
- Ensuring all rural residents have equitable access to Rural Development (RD) programs and benefits from RD funded projects; and
- Reducing climate pollution and increasing resilience to the impacts of climate change through economic support to rural communities.

In addition, the Agency highlights the importance of strengthening resiliency of the broader food supply chain, including through addressing current supply chain related disruptions. The Agency will consider applications as they are submitted. If available funding is less than what is requested by applications under consideration, the Agency will score each eligible application based on the point system described herein. When applications on hand have the same priority score, the Agency will give preference to applications involving guaranteed loans from veterans.

Hemp Related Projects: Please note that no assistance or funding from this program can be provided to a hemp producer unless they have a valid license issued from an approved State, Tribal or Federal plan as per section 10113 of the Agriculture Improvement Act of 2018, Public Law 115–334. Verification of valid hemp licenses will occur at the time of award.

A. Program Description and Overview

(a) *Purpose of the program.* Food Supply Chain (FSC) guaranteed loans are available to qualified applicants and projects to facilitate financing for the start-up or expansion of activities in the middle of the food supply chain, particularly the aggregation, processing, manufacturing, storing, transporting, wholesaling, or distribution of food, to increase capacity and help create a more resilient, diverse, and secure U.S. food supply chain. As reflected in the public comments to AMS–TM–21–0034, *Supply Chains for the Production of Agricultural Commodities and Food Products,* 86 FR 20652 (April 21, 2021), financing for infrastructure as a strategy to strengthen the food supply chain was identified as a need not only for small and mid-sized meat and poultry processors, but across other stages of the food supply chain, including distribution and aggregation.

This program will expand access to financing for food systems infrastructure in the near term and will serve as a pilot program to inform the other programs authorized under Section 1001 of the American Rescue Plan Act of 2021 (American Rescue Plan Act). This program will facilitate access to affordable capital to address the ongoing need for food systems enterprises in America's rural and urban communities, as there are no geographic restrictions.

(b) *Statutory authority.* Section 1001(b)(4) of the American Rescue Plan Act authorizes the Secretary of Agriculture to ''. . . make loans and grants and provide other assistance to maintain and improve food and
