# Supplementary material for: Exploring Methods to Evaluate HPAI Transmission Risk in Iowa During Peak HPAI Incidence, February 2022–December 2023
Source: Int J Environ Res Public Health. 2025 Mar 10;22(3):400. doi: 10.3390/ijerph22030400 (PMC11942192; doi:10.3390/ijerph22030400)
Supplement: Supplementary file 1 [file ijerph-22-00400-s001.zip › File S3.pdf]

163.3C Foreign animal disease preparedness and response strategy.

1. The department shall develop and establish a foreign animal disease preparedness and response strategy for use by the department in order to prevent, control, or eradicate the transmission of foreign animal diseases among populations of animals. The strategy may be part of the department's veterinary emergency preparedness and response services as provided in section 163.3A. The strategy shall provide additional expertise and resources to increase biosecurity efforts that assist in the prevention of a foreign animal disease outbreak in this state. In developing and establishing the strategy, the department shall consult with interested persons including but not limited to the following:

a. The Iowa cattlemen's association.

b. The Iowa state dairy association.

c. The Iowa pork producers association.

d. The Iowa sheep producers industry association.

e. The Iowa turkey federation.

f. The Iowa poultry association.

g. The college of veterinary medicine at Iowa state university.

h. The livestock health advisory council created in section 267.2.

2. The department shall implement the foreign animal disease preparedness and response strategy if necessary to prevent, control, or eradicate the transmission and incidence of foreign animal diseases that may threaten or actually threaten animals in this state. In implementing the strategy, the department may utilize emergency response measures as otherwise required under section 163.3A. The department may but is not required to consult with interested persons when implementing the strategy.

3. *a.* In developing and establishing a foreign animal disease preparedness and response strategy, the department may collect, maintain, and use information related to the registration and identification of any premises where animals are kept. The information may include but is not limited to all of the following:

(1) The name, address, and contact information of an interested person.

(2) The location of the premises where the animals are kept.

(3) An identification number assigned to the premises where the animals are kept.

b. The information described in paragraph *"a"* is a confidential record as provided in section 22.7. Nothing in this subsection limits the department in acting as the lawful custodian of the confidential record from disclosing the record or any part of the record to another person if the department determines that such disclosure will assist in implementing, administering, or enforcing the foreign animal disease preparedness and response strategy.

2017 Acts, ch 168, §29; 2020 Acts, ch 1036, §4 – 6, 16; 2021 Acts, ch 149, §3

Referred to in **§22.7(39A)**, 163.3B, 163.3F
