# Supplementary material for: Exploring Methods to Evaluate HPAI Transmission Risk in Iowa During Peak HPAI Incidence, February 2022–December 2023
Source: Int J Environ Res Public Health. 2025 Mar 10;22(3):400. doi: 10.3390/ijerph22030400 (PMC11942192; doi:10.3390/ijerph22030400)
Supplement: Supplementary file 1 [file ijerph-22-00400-s001.zip › R code.pdf]

R code for Pearson's chi-square:

```
> table_data3 <- table(RCdata$CG.Total.GE.Mdn, RCdata$HPAI.Positive.Counties)
> chi_square_test3 <- chisq.test(table_data3)
> print(chi_square_test3)

        Pearson's Chi-squared test with Yates' continuity correction

data:  table_data3
X-squared = 4.2916, df = 1, p-value = 0.0383


table_data1 <- table(Rdata$Inland_water_veg_mean_cat, Rdata$HPAI.Positive.Cou
nties)
> chi_square_test1 <- chisq.test(table_data1)
> print(chi_square_test1)

        Pearson's Chi-squared test with Yates' continuity correction

data:  table_data1
X-squared = 0.13674, df = 1, p-value = 0.7115
```

```
R code for histograms

# Histogram with smoothing curve for Inland.Water.Surface.Area.km2
> ggplot(Rdata, aes(x = Inland.Water.Surface.Area.km2)) +
geom_histogram(aes(y = ..density..), binwidth = 10, fill = "blue", alpha = 0.
5) +
geom_density(color = "red", size = 1) +
labs(title = "Histogram with Smoothing Curve for Inland Water Surface Area",
 x = "Inland Water Surface Area (km²)",
 y = "Density") +
theme_minimal()
```

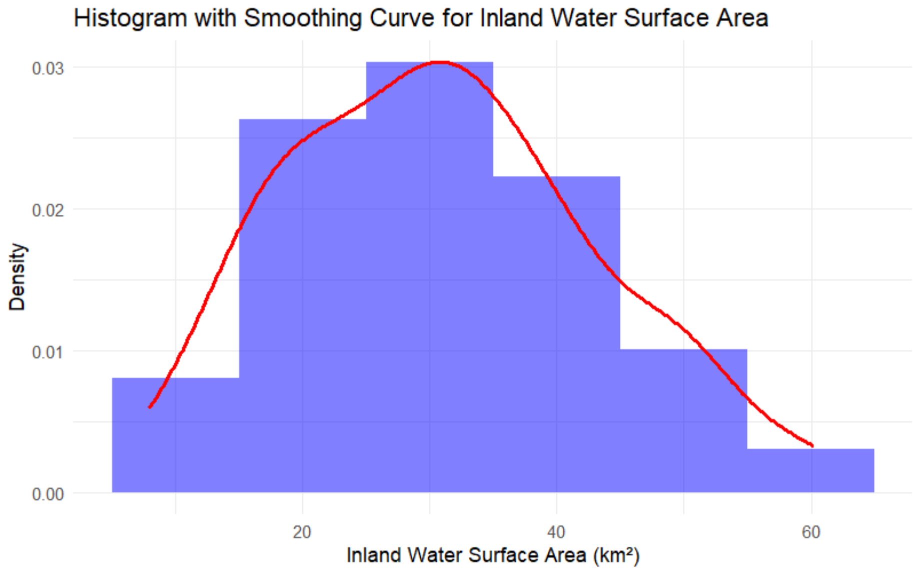

```
ggplot(Rdata, aes(x = Canada.Goose.Total.Counts)) +
geom_histogram(aes(y = ..density..), binwidth = 3500, fill = "green", alpha =
0.5) +
geom_density(color = "red", size = 1) +
labs(title = "Histogram with Smoothing Curve for Canada Goose Total Counts",
x = "Canada Goose Total Counts",
y = "Density") +
theme_minimal()
```

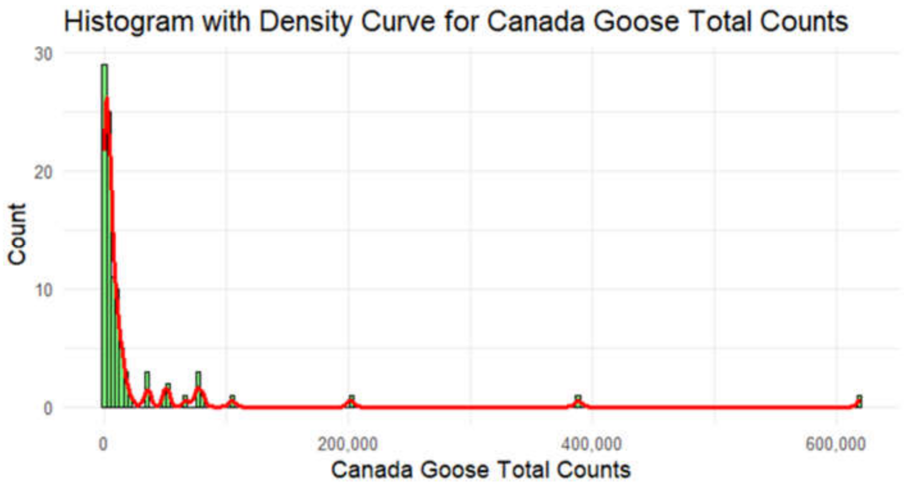


```
# Calculate median and mean for Canada.Goose.Total.Counts
> median_canada_goose <- median(Rdata$Canada.Goose.Total.Counts, na.rm = TRUE
)
> mean_canada_goose <- mean(Rdata$Canada.Goose.Total.Counts, na.rm = TRUE)
>
> # Calculate median and mean for Inland.Water.Surface.Area.km2
> median_inland_water <- median(Rdata$Inland.Water.Surface.Area.km2, na.rm =
TRUE)
> mean_inland_water <- mean(Rdata$Inland.Water.Surface.Area.km2, na.rm = TRUE
)
>
> # Print the results
> cat("Median of Canada Goose Total Counts:", median_canada_goose, "\n")
Median of Canada Goose Total Counts: 3947
> cat("Mean of Canada Goose Total Counts:", mean_canada_goose, "\n")
Mean of Canada Goose Total Counts: 23936.02
> cat("Median of Inland Water Surface Area (km²):", median_inland_water, "\n"
)
Median of Inland Water Surface Area (km²): 30.39
> cat("Mean of Inland Water Surface Area (km²):", mean_inland_water, "\n")
Mean of Inland Water Surface Area (km²): 30.93495
```
